# Hospital‐level compliance with the commission on cancer’s quality of care measures and the association with patient survival

**DOI:** 10.1002/cam4.3875

**Published:** 2021-05-04

**Authors:** Daniel P. Nussbaum, Christel N. Rushing, Zhifei Sun, Babatunde A. Yerokun, Mathias Worni, Robert S. Saunders, Mark B. McClellan, Donna Niedzwiecki, Rachel A. Greenup, Dan G. Blazer

**Affiliations:** ^1^ Department of Surgery Duke University Durham NC USA; ^2^ Department of Biostatistics and Bioinformatics Duke University Durham NC USA; ^3^ Department of Visceral Surgery, Clarunis University Centre for Gastrointestinal and Liver Diseases St. Clara Hospital and University Hospital Basel Switzerland; ^4^ Swiss Institute for Translational and Entrepreneurial Medicine Bern Switzerland; ^5^ Duke University Robert J. Margolis Center for Health Policy Durham NC USA; ^6^ Department of Surgery and Population Health Sciences Duke University Duke Cancer Institute Durham NC USA; ^7^ Department of Surgery Duke University Duke Cancer Institute Durham NC USA

**Keywords:** cancer, hospitals, process measures, quality of care

## Abstract

**Background:**

Quality measurement has become a priority for national healthcare reform, and valid measures are necessary to discriminate hospital performance and support value‐based healthcare delivery. The Commission on Cancer (CoC) is the largest cancer‐specific accreditor of hospital quality in the United States and has implemented Quality of Care Measures to evaluate cancer care delivery. However, none has been formally tested as a valid metric for assessing hospital performance based on actual patient outcomes.

**Methods:**

Eligibility and compliance with the Quality of Care Measures are reported within the National Cancer Database, which also captures data for robust patient‐level risk adjustment. Hospital‐level compliance was calculated for the core measures, and the association with patient survival was tested using Cox regression.

**Results:**

Seven hundred sixty‐eight thousand nine hundred sixty‐nine unique cancer cases were included from 1323 facilities. Increasing hospital‐level compliance was associated with improved survival for only two measures, including a 35% reduced risk of mortality for the gastric cancer measure G15RLN (HR 0.65, 95% CI 0.58–0.72) and a 19% reduced risk of mortality for the colon cancer measure 12RLN (HR 0.81, 95% CI 0.77–0.85). For the lung cancer measure LNoSurg, increasing compliance was paradoxically associated with an increased risk of mortality (HR 1.14, 95% CI 1.08–1.20). For the remaining measures, hospital‐level compliance demonstrated no consistent association with patient survival.

**Conclusion:**

Hospital‐level compliance with the CoC’s Quality of Care Measures is not uniformly aligned with patient survival. In their current form, these measures do not reliably discriminate hospital performance and are limited as a tool for value‐based healthcare delivery.

## Graphical abstract

The Commission on Cancer is the largest cancer‐specific accreditor of hospital quality in the United States and has implemented Quality of Care Measures to evaluate quality cancer care delivery. This study represents the first comprehensive evaluation of the Quality of Care Measures as valid metrics for assessing hospital performance based on actual patient outcomes.

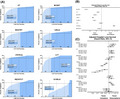

## 1 INTRODUCTION

Quality measurement has increasingly become a priority for national healthcare reform as the United States shifts from fee‐for‐service to value‐based reimbursement models. Over the past two decades, however, rapid proliferation of quality measures has been accompanied by a growing concern that current measurement systems increase the costs and complexity of healthcare delivery without providing the perceived benefits of resolving performance and facilitating value.^1^, ^2^ Studies evaluating measurement systems are inherently challenging, in part due to a lack of centralized data that can couple hospital‐level measure adherence with meaningful, risk‐adjusted patient outcomes; thus, few have been formally tested with regard to predicting hospital performance.^3^, ^4^, ^5^, ^6^, ^7^, ^8^, ^9^, ^10^

Oncology has been a particular target for contemporary measurement reform due to the wide variability in healthcare utilization, high costs of treatment, and patient‐centeredness that characterizes cancer care.^11^, ^12^, ^13^, ^14^ Process measures in cancer care are broadly being implemented into value‐based healthcare models. Moreover, hospital performance has increasingly become publicly available and tied to payments and reimbursements, and accreditation by reputable professional organizations hinges on compliance with certain metrics. While multiple organizations have proposed process‐driven measures to evaluate quality, however, none has been formally tested as a valid metric for assessing hospital performance based on actual patient outcomes.^11^, ^12^, ^13^, ^14^

The Commission on Cancer (CoC) is the largest cancer‐specific accreditor of hospital quality in the United States, and greater than 70% of all cancer patients receive care at approximately 1500 CoC‐accredited institutions.^15^, ^16^, ^17^ As part of the accreditation process, the CoC has implemented Quality of Care Measures; beyond accreditation, these process measures are intended for public reporting, payment incentive, and the selection of providers by consumers and payers.^11^, ^18^ In addition, data from CoC‐accredited institutions are maintained and reported by the National Cancer Database, including both compliance status with the Quality of Care Measures as well as comprehensive demographic, clinicopathologic, treatment, and survival data, providing a unique opportunity to test the discriminative ability of a quality measurement system with regard to risk‐adjusted, hospital‐level outcomes. The objective of this study was to evaluate whether adherence to the Quality of Care Measures discriminates hospital performance based on patient outcomes, using covariate‐adjusted overall survival as our primary endpoint.

## 2 METHODS

### 2.1 Data source and study population

The National Cancer Database (NCDB) is a nationwide oncology outcomes database with comprehensive demographic, clinicopathologic, treatment, and survival data.^19^ CoC‐accredited programs use the NCDB to collect, analyze, and report all patient data, including those used to define Quality of Care Measures eligibility and compliance.^20^ Precise coding algorithms for the Quality of Care Measures are publically available,^11^ and these were used to identify eligible and compliant patients within the 2015 Participant User File for patients diagnosed from 2010 to 2014 (most recent available survival data). Quality of Care Measures are categorized as Surveillance, Quality Improvement, or Accountability based on increasing levels of supporting evidence, in keeping with National Quality Forum (NQF) standards.^18^ The eight Quality Improvement and Accountability measures evaluated in this study were specifically selected as these are the ones currently utilized for CoC accreditation, and thus they each also have an established Expected Estimated Performance Rate, the standard set for each measure to maintain accrediation.^18^, ^21^

### 2.2 Patient‐level and hospital‐level compliance

Eligible cases for each measure were defined as compliant or noncompliant, with missing compliance status treated as noncompliant, in keeping with the CoC’s definitions. Hospital‐level compliance was calculated as the percentage of compliant cases among all eligible cases reported by each facility, and subsequently, facilities were categorized into performance quartiles. Facilities were also dichotomized by Expected Performance Rate—the standard set for each measure to maintain accreditation^18^, ^21^ —and were evaluated continuously by percent compliance. The primary hospital‐level compliance metric was compliance quartile, with Expected Performance Rate and percent compliance used as secondary metrics.

### 2.3 Survival analyses

Overall survival was defined as the time from diagnosis to death due to any cause. The Kaplan‐Meier method was used to calculate unadjusted survival probabilities with 95% confidence intervals (CIs). Unadjusted and adjusted hazard ratios (HRs) with 95% CIs were estimated using Cox regression. Multiple imputation, using five imputations, was performed for covariates with missing data.

The primary outcome was overall survival adjusted for patient demographic and clinicopathologic variables (Model 1). Two additional multivariable models were developed to also include hospital variables (Model 2) and hospital/treatment variables (Model 3), and HRs from these were considered secondary outcomes. For Model 3, if treatment variables were included in the compliance definition, they were excluded from the analysis. A complete list of covariates for each model can be found in the Supplementary Appendix. Statistical analyses were performed using SAS version 9.4 and R version 3.4. *p*‐values were adjusted for multiplicity using the Holm‐Bonferroni method. This study was granted exempt status by the Duke University Institutional Review Board.

## 3 RESULTS

**FIGURE 1:**
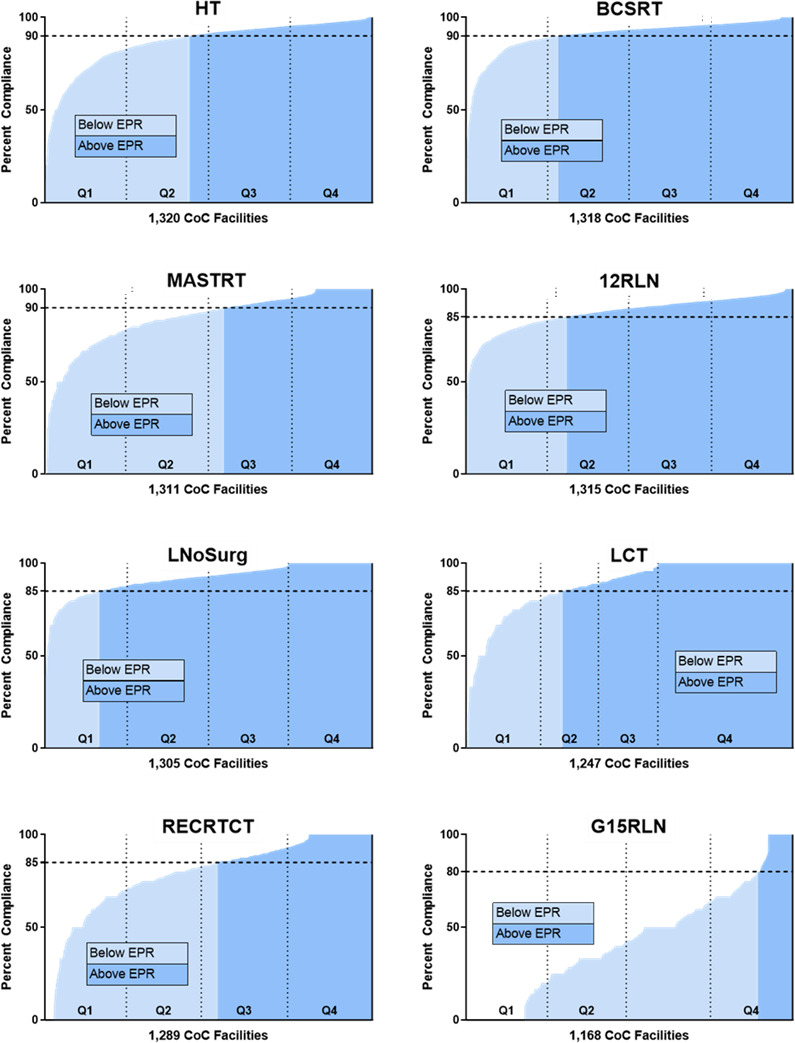
Hospital compliance for each QCM across all CoC facilities with at least one eligible case, with quartile‐based and EPR‐based divisions indicated. Abbreviations: CoC, Commission on Cancer; EPR, estimated performance rate; QCM, Quality of Care Measure

In total, 768,969 unique cancer cases were included from 1323 facilities: 481,094 breast cancers, 183,026 colon cancers, 63,227 non‐small cell lung cancers, 27,325 rectal cancers, and 14,287 gastric cancers (Table 1). Among breast and lung cancer cases, 171,902 and 4075, respectively, qualified for more than one measure. For each measure, the calculated hospital compliance distributions are shown in Figure 1 and Table S1. Overall median hospital compliance ranged from 42.9% for G15RLN (Interquartile Range [IQR] 21.1–64.3%) to 93.5% or LCT (IQR 82.8–100%). Baseline demographic, clinicopathologic, hospital, and treatment data are shown in Table S2–S9.

**TABLE 1:** Commission on Cancer Quality of Care Measures

Measure Name	Measure Type	EPR	Number of Cases	Measure Description
Breast Cancer				
BCSRT	Accountability	90%	346,085	Radiation therapy is administered within 1 year (365 days) of diagnosis for women under age 70 receiving breast‐conserving surgery for breast cancer.
HT	Accountability	90%	450,421	Tamoxifen or third‐generation aromatase inhibitor is recommended or administered within 1 year (365 days) of diagnosis for women with AJCC T1cN0 M0, or stage IB ‐ III hormone receptor‐positive breast cancer.
MASTRT	Accountability	90%	55,763	Radiation therapy is recommended or administered following any mastectomy within 1 year (365 days) of diagnosis of breast cancer for women with ≥4 positive regional lymph nodes.
Colon Cancer				
12RLN	Quality Improvement	85%	285,831	At least 12 regional lymph nodes are removed and pathologically examined for resected colon cancer.
Gastric Cancer				
G15RLN	Quality Improvement	80%	17,795	At least 15 regional lymph nodes are removed and pathologically examined for resected gastric cancer.
Non‐Small Cell Lung Cancer				
LCT	Quality Improvement	85%	30,167	Systemic chemotherapy is administered within 4 months to day preoperatively or day of surgery to 6 months postoperatively, or it is recommended for surgically resected cases with pathologic, lymph node‐positive (pN1) and (pN2) NSCLC.
LNoSurg	Quality Improvement	85%	68,835	Surgery is not the first course of treatment for cN2, M0 lung cases.
Rectal Cancer				
RECRTCT	Quality Improvement	85%	30,946	Preoperative chemo and radiation are administered for clinical AJCC T3 N0, T4 N0, or Stage III; or Postoperative chemo and radiation are administered within 180 days of diagnosis for clinical AJCC T1‐2 N0 with pathologic AJCC T3 N0, T4 N0, or Stage III; or treatment is recommended; for patients under the age of 80 receiving resection for rectal cancer.

Abbreviations: EPR‐expected performance rate.

### 3.1 Survival based on patient‐level compliance

**FIGURE 2:**
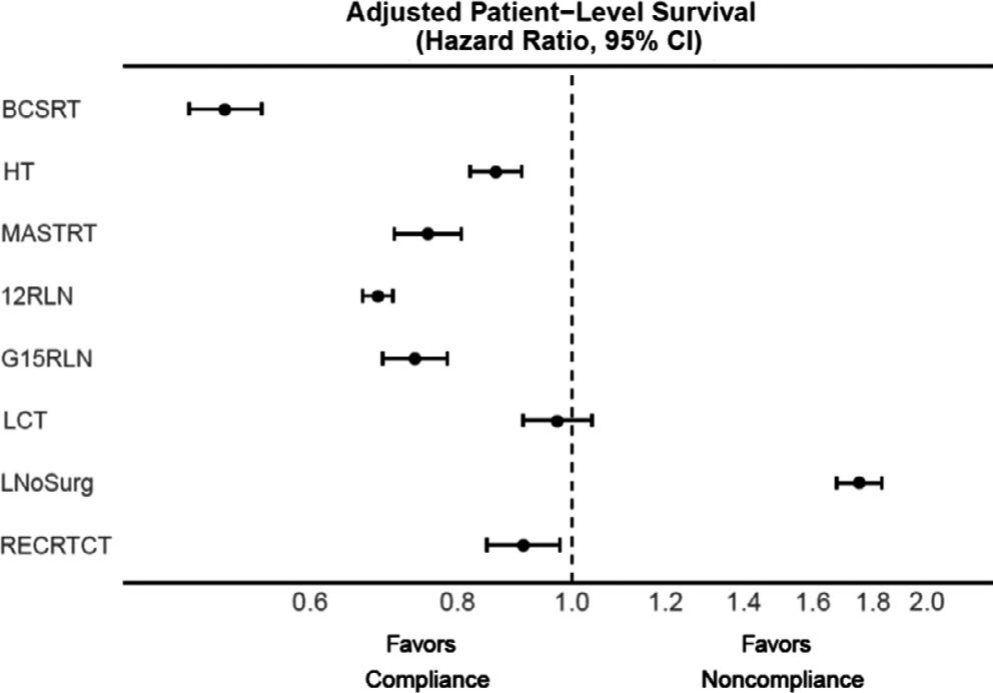
Forest plot representing the association of binary patient‐level compliance with overall survival for each QCM. Hazard ratios are adjusted for patient demographic and clinicopathologic variables in Cox proportional hazard models. Abbreviations: CI, confidence interval; QCM, Quality of Care Measure

Patient‐level survival estimates are shown in Figure 2, Figure S1, and Table S10. For six of eight measures, there was a significant improvement in overall survival for compliant versus noncompliant patients. Of these, the adjusted HR for compliant patients ranged from 0.51 (95% CI 0.47–0.55) for BCSRT to 0.91 (95% CI 0.85–0.98) for RECRTCT. For the lung cancer measures, compliance with LCT was not associated with patient‐level outcomes, and for LNoSurg, compliant patients had a paradoxical survival disadvantage compared to noncompliant patients. Two‐ and five‐year unadjusted survival probabilities were 36.7% and 17.8% for compliant patients (95% CI 36.2–37.2% and 17.3–18.3%), compared to 62.7% and 36.9% for noncompliant patients (95% CI 61.2–64.1% and 35.0–38.7%), resulting in an adjusted HR of 1.75 (95% CI 1.68–1.83). By measure definition, 100% of noncompliant patients underwent upfront surgery, while only 7.1% of compliant patients ever underwent resection. Moreover, 16.4% of all metric eligible patients for LNoSurg received no treatment at all (e.g., chemotherapy or radiation therapy), but were nonetheless considered compliant by measure definition.

### 3.2 Survival based on hospital‐level compliance

**FIGURE 3:**
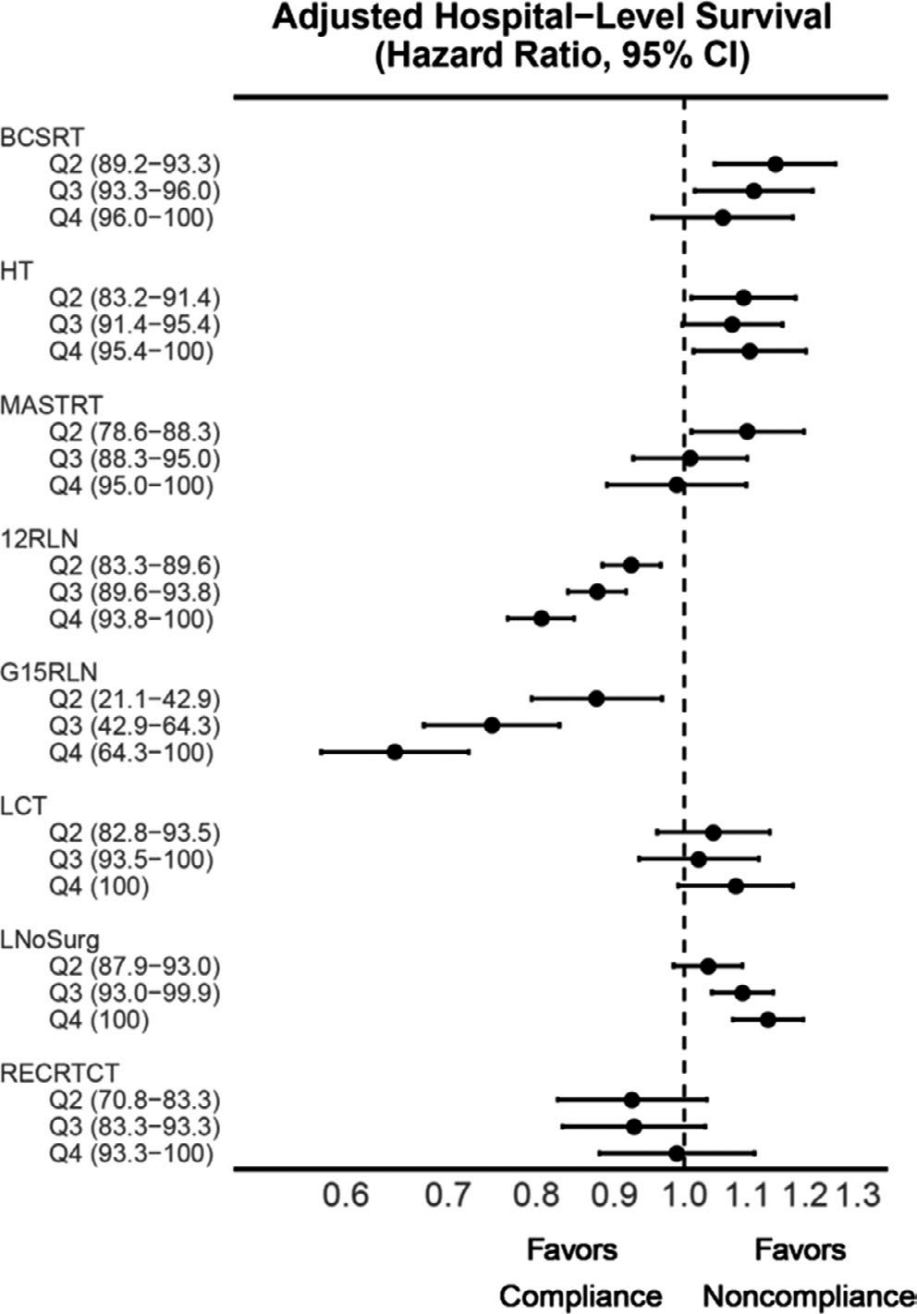
Forest plot representing the association of hospital‐level, quartile‐based compliance with overall survival for each QCM. Hazard ratios are adjusted for patient demographic and clinicopathologic variables in Cox proportional hazard models. Abbreviations: CI, confidence interval; QCM, Quality of Care Measure

Unadjusted hospital‐level survival estimates are shown in Figures S2–S3 and Tables S11–S12. Covariate‐adjusted survival is shown in Figure 3 and Table 2. Increasing hospital‐level compliance was associated with improved survival for only two measures: G15RLN and 12RLN. For G15RLN, treatment at the most compliant hospitals was associated with a 35% reduced risk of mortality (HR 0.65, 95% CI 0.58–0.72) relative to the least compliant hospitals. Similarly, for 12RLN, treatment at the most compliant hospitals was associated with a 19% reduced risk of mortality (HR 0.81, 95% CI 0.77–0.85). The associations between compliance and survival were similar in the additional two adjustment models.

**TABLE 2:** Association between patient‐level and hospital‐level measure compliance and overall survival

Measure Name	Compliance Group	Unadjusted				Adjusted for Demographic and Clinicopathologic Variables (Model 1)				Adjusted for Demographic, Clinicopathologic, and Hospital Variables (Model 2)				Adjusted for Demographic, Clinicopathologic, Hospital, and Treatment Variables (Model 3)			
		HR	95% CI		*p*‐value	HR	95% CI		*p*‐value	HR	95% CI		*p*‐value	HR	95% CI		*p*‐value
Breast Cancer																	
BCSRT	Patient‐Level																
	Compliant	0.41	0.38	0.45	<0.001	0.51	0.47	0.55	<0.001	0.50	0.46	0.53	<0.001	0.55	0.51	0.59	<0.001
	Hospital‐Level																
	Q2	1.11	1.00	1.22	0.700	1.15	1.05	1.26	0.063	1.09	0.99	1.19	1.000	1.12	1.02	1.23	0.226
	Q3	1.06	0.96	1.16	1.000	1.11	1.02	1.21	0.325	1.08	0.98	1.18	1.000	1.12	1.03	1.23	0.192
	Q4	0.96	0.85	1.07	1.000	1.06	0.95	1.18	1.000	0.99	0.89	1.11	1.000	1.04	0.94	1.16	1.000
	Above EPR	1.00	0.92	1.09	1.000	1.07	0.99	1.15	1.000	1.02	0.94	1.10	1.000	1.06	0.98	1.14	1.000
	Continuous	1.00	1.00	1.01	1.000	1.04	1.00	1.09	0.707	1.02	0.98	1.07	1.000	1.05	1.01	1.10	0.223
HT	Patient‐Level																
	Compliant	0.79	0.75	0.84	<0.001	0.86	0.82	0.91	<0.001	0.84	0.80	0.88	<0.001	0.86	0.82	0.90	<0.001
	Hospital‐Level																
	Q2	1.05	0.96	1.15	1.000	1.09	1.01	1.18	0.453	1.04	0.97	1.12	1.0000	1.06	0.99	1.14	1.000
	Q3	1.03	0.95	1.12	1.000	1.08	1.00	1.16	1.000	1.01	0.94	1.09	1.0000	1.04	0.96	1.12	1.000
	Q4	1.04	0.94	1.16	1.000	1.10	1.01	1.20	0.431	1.03	0.94	1.11	1.0000	1.05	0.97	1.15	1.000
	Above EPR	1.01	0.95	1.08	1.000	1.04	0.99	1.10	1.000	1.00	0.94	1.05	1.0000	1.02	0.96	1.07	1.000
	Continuous	1.00	1.00	1.00	1.000	1.03	1.01	1.05	0.267	1.01	0.99	1.03	1.0000	1.02	1.00	1.04	1.000
MASTRT	Patient‐Level																
	Compliant	0.67	0.63	0.71	<0.001	0.76	0.71	0.81	<0.001	0.74	0.69	0.79	<0.001	0.82	0.77	0.88	<0.001
	Hospital‐Level																
	Q2	1.02	0.93	1.11	1.000	1.10	1.01	1.20	0.504	1.07	0.98	1.16	1.000	1.12	1.03	1.22	0.142
	Q3	0.94	0.86	1.02	1.000	1.01	0.93	1.10	1.000	0.98	0.90	1.07	1.000	1.05	0.96	1.14	1.000
	Q4	0.91	0.81	1.02	1.000	0.99	0.89	1.10	1.000	0.96	0.87	1.05	1.000	1.02	0.92	1.13	1.000
	Above EPR	0.93	0.88	1.00	0.65	0.97	0.91	1.03	1.000	0.96	0.90	1.01	1.000	0.98	0.93	1.04	1.000
	Continuous	1.00	1.00	1.00	1.000	1.00	0.98	1.03	1.000	1.00	0.97	1.02	1.000	1.02	1.00	1.04	1.000
Colon Cancer																	
12RLN	Patient‐Level																
	Compliant	0.73	0.71	0.75	<0.001	0.69	0.67	0.71	<0.001	0.69	0.68	0.72	<0.001	0.69	0.67	0.71	<0.001
	Hospital‐Level																
	Q2	0.90	0.86	0.94	<0.001	0.92	0.88	0.97	0.001	0.95	0.91	1.00	0.044	0.95	0.91	0.99	0.044
	Q3	0.85	0.82	0.90	<0.001	0.88	0.84	0.92	<0.001	0.92	0.88	0.96	<0.001	0.92	0.88	0.96	<0.001
	Q4	0.77	0.73	0.81	<0.001	0.81	0.77	0.85	<0.001	0.85	0.81	0.89	<0.001	0.84	0.80	0.89	<0.001
	Above EPR	0.84	0.81	0.87	<0.001	0.86	0.83	0.89	<0.001	0.90	0.87	0.93	<0.001	0.90	0.87	0.93	<0.001
	Continuous	0.99	0.99	0.99	<0.001	0.92	0.90	0.94	<0.001	0.94	0.92	0.96	<0.001	0.94	0.92	0.96	<0.001
Gastric Cancer																	
G15RLN	Patient‐Level																
	Compliant	0.91	0.85	0.98	0.082	0.74	0.69	0.78	<0.001	0.79	0.75	0.84	<0.001	0.77	0.73	0.82	<0.001
	Hospital‐Level																
	Q2	0.92	0.84	1.01	0.238	0.88	0.79	0.97	0.059	0.92	0.83	1.02	0.238	0.92	0.83	1.02	0.238
	Q3	0.78	0.70	0.86	<0.001	0.75	0.68	0.83	<0.001	0.84	0.76	0.93	0.004	0.82	0.74	0.91	0.002
	Q4	0.65	0.57	0.74	<0.001	0.65	0.58	0.72	<0.001	0.79	0.72	0.88	<0.001	0.78	0.70	0.87	<0.001
	Above EPR	0.67	0.52	0.86	0.017	0.75	0.63	0.90	0.017	0.87	0.78	0.98	0.098	0.87	0.78	0.98	0.088
	Continuous	0.99	0.99	1.00	<0.001	0.94	0.92	0.95	<0.001	0.97	0.95	0.98	<0.001	0.96	0.95	0.98	<0.001
Non‐Small Cell Lung Cancer																	
LCT	Patient‐Level																
	Compliant	0.95	0.89	1.02	1.000	0.97	0.91	1.04	1.000	0.96	0.90	1.02	1.000	0.94	0.88	1.00	1.000
	Hospital‐Level																
	Q2	1.05	0.94	1.16	1.000	1.07	0.97	1.18	1.000	1.06	0.97	1.16	1.000	1.06	0.97	1.16	1.000
	Q3	1.03	0.93	1.13	1.000	1.05	0.96	1.16	1.000	1.05	0.96	1.14	1.000	1.05	0.96	1.14	1.000
	Q4	1.09	0.98	1.20	1.000	1.10	1.00	1.21	1.000	1.05	0.97	1.14	1.000	1.04	0.95	1.13	1.000
	Above EPR	1.03	0.95	1.11	1.000	1.04	0.97	1.12	1.000	1.02	0.96	1.09	1.000	1.02	0.96	1.09	1.000
	Continuous	1.00	1.00	1.01	1.000	1.03	1.01	1.06	0.383	1.02	1.00	1.05	0.719	1.02	1.00	1.04	1.000
LNoSurg	Patient‐Level																
	Compliant	1.95	1.87	2.03	<0.001	1.75	1.68	1.83	<0.001	1.74	1.66	1.82	<0.001	2.23	2.12	2.35	<0.001
	Hospital‐Level																
	Q2	1.06	1.00	1.12	0.151	1.04	0.98	1.09	0.171	1.05	1.00	1.10	0.092	1.10	1.05	1.15	<0.001
	Q3	1.14	1.08	1.20	<0.001	1.09	1.04	1.14	0.002	1.08	1.04	1.13	0.002	1.15	1.10	1.21	<0.001
	Q4	1.21	1.14	1.28	<0.001	1.14	1.08	1.20	<0.001	1.10	1.05	1.16	0.002	1.20	1.13	1.27	<0.001
	Above EPR	1.11	1.05	1.17	0.002	1.07	1.02	1.13	0.036	1.07	1.02	1.12	0.027	1.15	1.09	1.21	<0.001
	Continuous	1.01	1.01	1.01	<0.001	1.06	1.03	1.09	<0.001	1.05	1.02	1.08	0.001	1.10	1.07	1.13	<0.001
Rectal Cancer																	
RECRTCT	Patient‐Level																
	Compliant	0.78	0.73	0.84	<0.001	0.91	0.85	0.98	0.172	0.90	0.84	0.97	0.098	0.89	0.83	0.95	0.025
	Hospital‐Level																
	Q2	0.88	0.79	0.99	0.509	0.92	0.83	1.04	1.000	0.92	0.83	1.03	1.000	0.93	0.83	1.04	1.000
	Q3	0.85	0.76	0.95	0.120	0.93	0.83	1.03	1.000	0.91	0.82	1.02	1.000	0.91	0.82	1.02	1.000
	Q4	0.91	0.81	1.03	1.000	0.99	0.88	1.11	1.000	0.96	0.85	1.08	1.000	0.96	0.85	1.08	1.000
	Above EPR	0.95	0.87	1.03	1.000	1.00	0.93	1.08	1.000	0.97	0.90	1.05	1.000	0.97	0.90	1.05	1.000
	Continuous	1.00	0.99	1.00	0.159	0.99	0.97	1.01	1.000	0.98	0.96	1.01	1.000	0.98	0.96	1.01	1.000

Reference comparisons for the compliance groups: patient‐level compliant (ref: noncompliant), hospital‐level Q2, Q3, and Q4 (ref: Q1), hospital‐level above EPR (ref: below EPR), hospital‐level continuous (per 10%);

Abbreviations: CI, confidence interval; EPR, expected performance rate, Q, quartile; HR, hazard ratio.

For five measures, hospital‐level compliance demonstrated no consistent association with patient survival. This included all three breast cancer measures (BCSRT, HT, and MASTRT), as well as the lung cancer measure LCT and the rectal cancer measure RECRTCT. Finally, for the lung cancer measure LNoSurg, treatment at the most compliant hospitals was associated with a 14% increased risk of mortality following adjustment for patient demographic and clinicopathologic variables (HR 1.14, 95% CI 1.08–1.20). Similar results were observed in all adjustment models.

Compliance based on Expected Performance Rates and measured as a continuous variable was used as secondary metrics. Compliance with the established Expected Performance Rates ranged from 10.6% of all hospitals for G15RLN to 83.4% for LNoSurg (Figure 1, Table S1). Similar to the primary analyses, a decreased adjusted risk of mortality was detected for only two measures: G15RLN (HR 0.75, 95% CI 0.63–0.90) and 12RLN (HR 0.86, 95% CI 0.83–0.89). Again, treatment at hospitals meeting the Expected Performance Rate was associated with an increased adjusted risk of mortality for LNoSurg (HR 1.07, 95% CI 1.02–1.13). For the remaining five measures, there was no statistically significant association with adjusted survival. Similarly, with compliance treated as a continuous variable, increasing hospital compliance was associated with improved adjusted survival for only G15RLN and 12RLN. Increasing compliance was associated with inferior survival for LNoSurg, while there was no statistically significant association between hospital compliance and survival for the remaining five measures.

## 4 DISCUSSION

In 2002, the federally sponsored Quality of Cancer Care Performance Measures Project was initiated to establish a comprehensive measurement and reporting system for quality cancer care.^22^ As part of an open “call for measures”—overseen by the NQF—the CoC submitted eight measures, five of which were endorsed as the original Quality of Care Measures in 2008.^18^ Over the past decade, the CoC has expanded to 23 measures across 10 disease sites, a process paralleled nationally by an expansion of quality measurement programs across multiple facets of healthcare delivery.^11^, ^12^, ^13^, ^14^ As quality measures are now being implemented in alternative payment models and public reporting programs,^23^ there is growing concern that this rapid proliferation is increasing the cost and complexity of healthcare delivery without the intended benefits of discriminating hospital performance and promoting value.^1^, ^2^ Evaluation of the CoC’s Quality of Care Measures thus provides insight into measurement systems in general, while specifically examining a quality program with current implications on health policy. This study demonstrates that while patient‐level compliance with the majority of the core measures is associated with patient survival, increasing hospital‐level compliance is associated with improved survival for only two measures (G15RLN, 12RLN); hospital‐level compliance with the remaining six measures is either paradoxically associated with inferior survival (LNoSurg), or demonstrates no consistent association with patient survival (HT, MASTRT, BCSRT, RECRTCT, LCT).

While these results are counterintuitive, they should not be entirely unexpected given the overall paucity of evidence supporting the ability of process measures to discriminate hospital performance. Several well‐designed studies among cardiology patients have found that hospital‐level adherence to individual process measures—such as the acute myocardial infarction quality measures developed by the Centers for Medicare & Medicaid Services (CMS) and the Joint Commission on Accreditation of Healthcare Organizations (JCAHO)—is an unreliable predictor of patient outcomes.^3^, ^4^, ^5^, ^6^ Similar findings were observed for the Children's Asthma Care measure set^7^ —designed by JCAHO and endorsed by the NQF as the first core measure set for hospitalized children—as well as Medicare's Hospital Compare Performance Measures for pneumonia, myocardial infarction, and heart failure.^8^ Studies evaluating quality measures for cancer care are even more limited. In 2007, Wong et al. evaluated hospital‐level adherence with lymph node retrieval among patients undergoing colectomy for colon cancer and found that increasing hospital compliance was not associated with improved staging, appropriate use of adjuvant chemotherapy, or patient survival.^9^ These specific results differed from our findings, perhaps because their study included only Medicare patients greater than 65 years old who were treated prior to the use of modern multiagent adjuvant chemotherapy.^24^, ^25^, ^26^, ^27^ Interestingly, in a 2017 study evaluating three NQF‐endorsed colon cancer measures, Mason et al. found that while individual measures failed to predict survival, a composite of these measures was predictive of hospital‐level mortality.^10^ Other studies evaluating process measures in cancer have focused solely on patient‐level outcomes,^28^, ^29^, ^30^ and have not examined whether these translate to hospital‐level performance.

The limited ability to discriminate hospital performance is particularly problematic given the measures’ use in accreditation and intended purpose for public reporting, payment incentive, and hospital selection by patients and insurers. Certain measures have already been introduced into federal programs such as the Prospective Payment System‐Exempt Cancer Hospital Quality Reporting Program^31^ and the Oncology Care Model,^32^ and are also included in Medicare's Hospital Compare resource.^33^ The function of these measures—and their broader expansion—is also worrisome considering the financial burden, time‐intensiveness, and lack of unified application of quality measurement systems in general. It is estimated that greater than $15 billion are spent annually on quality measurement reporting and that almost 800 work hours are required per physician per year.^34^ Moreover, of greater than 500 distinct measures in use, few are aligned between measurement programs or across private and public payment models, despite near universal expert opinion to standardize measure sets.^35^, ^36^

Our findings demonstrate that while compliance with most Quality of Care Measures is associated with patient‐level survival, the measurement system nonetheless fails to consistently discriminate hospital performance. Specifically, for LNoSurg, compliance at the patient level is itself associated with markedly inferior survival, and this translates to inferior hospital‐level outcomes. This is, in part, due to the CoC’s definition of compliance that includes any patient with clinical N2 non‐small cell lung cancer who does not receive surgery as initial treatment, including those who are confirmed to have received no treatment at all (16.4% of all metric‐eligible patients in this study). This contradicts current national cancer guidelines, which recommend upfront chemotherapy or chemoradiation, but also acknowledge that patients with resectable disease should be considered for surgery post‐treatment.^37^ For six of the remaining seven measures, patient‐level compliance is associated with improved survival, reflecting the high‐level data on which most of these measures are based. In fact, this underscores why these measures may serve as important benchmarks to facilitate evidence‐based care, as has previously been demonstrated for individual measures.^28^, ^29^, ^30^ Nonetheless, these patient‐level findings do not uniformly translate to improved hospital‐level outcomes, even among measures based on high‐level randomized data.^38^, ^39^, ^40^ This paradox suggests that important nuances in treatment planning between cancer patients and their physicians are not necessarily well‐captured at the hospital or health system level.

Notably, the two Quality of Care Measures that best discriminate hospital performance (G15RLN, 12RLN) are both based on an accepted treatment standard rather than conclusive evidence that lymph node yield itself results in improved survival (for gastric cancer, randomized trials have produced equivocal results regarding the impact of lymph node retrieval on survival,^41^, ^42^, ^43^, ^44^, ^45^ and for colon cancer, the mechanism underlying an association between lymph node yield and survival remains unclear^31^, ^46^, ^47^, ^48^, ^49^, ^50^). Traditionally, process measures have been founded on evidence‐based practices, with the assumption that adherence naturally leads to improvements in patient outcomes. While lymph node yield may seem like a singular value limited to surgical technique, it in fact likely represents elements of surgical approach, pathological assessment, and medical oncology decision‐making. Ultimately, it appears that a well‐designed measure does not need to be based on highly effective treatment, but rather should provide an accepted standard that reflects the entire continuum of high‐quality, multidisciplinary, disease‐based care.^32^, ^33^ These findings also raise the possibility that process measures from other organizations may be limited in their ability to discriminate outcomes. It is to the CoC’s great credit that the organization collects data on these measures, makes these data publicly available, and allows the research community to analyze these data.

As the United States shifts toward value‐based accountable healthcare, measurement systems are inherently required to define quality and reliably discriminate performance. The majority of quality measures are process‐based, typically derived from practices with proven patient‐level benefits. While evidence‐based treatments are a key component of high‐quality care, our study and others demonstrate that process measures based on these practices are often implemented without understanding their ability to accurately differentiate hospital quality. Alternatively, outcome measures reflect the direct impact of healthcare on the health status of patients. While theoretically an ideal measurement of quality, an impediment to outcome measures is the need for risk‐adjustment that accounts for complex patients, treatment factors that are often subspecialty‐specific, and challenges in capturing the patient experience.^33^ As payments and reimbursements become increasingly tied to publicly available performance data, findings from this study highlight the need to thoroughly understand how process measures perform, and contribute to outcome before they are implemented as components of quality measurement systems.

There are several limitations to this study. First, this is an observational, non‐randomized study of a nationwide clinical oncology database. However, the CoC specifically utilizes the NCDB to collect, analyze, and report data for all Quality of Care Measures. Thus, whereas prior studies have had to match measure‐specific registry data with separate clinical databases, the NCDB serves as the primary data source for measuring compliance, adjustment, and patient outcomes. Second, the association between Quality of Care Measures and patient outcomes does not prove causality. For example, we do not believe that greater adherence with breast cancer measures predicated on high‐level, randomized data actually fails to improve patient survival on a health system level. Rather, other factors such as the distinct reporting patterns of individual hospitals may influence the discriminative ability of these measures to reliably predict patient outcomes; we are currently investigating how even low levels of underreporting can impact the discriminative ability of the measures, and create the potential for vulnerabilities among quality measurement systems in general. Nonetheless, it should also be acknowledged that clinical trials serving as the foundation for process measures are typically limited to a highly selected and monitored subset of patients, and the implementation of treatments based on their results may have distinct effects when applied generally at the health system level. Third, we did not study one of the Quality of Care Measures utilized in CoC‐accreditation due to unavailable exclusion criteria (nBx); however, this does not impact the findings regarding the remaining eight Quality of Care Measures. Fourth, this study utilizes OS as its primary endpoint, and it must be acknowledged that other outcomes such as resource utilization and quality of life—which are inherently more difficult to quantify—may be equally important. To that end, it has previously been shown that it is challenging to directly compare survival outcomes between individual hospitals,^51^ and thus we instead focused on hospitals aggregated by compliance quartiles. Finally, previous studies have demonstrated that composites rather than individual process measures may be more useful in discriminating hospital quality; while it was beyond the scope of this current study, future work should evaluate the effectiveness of the Quality of Care Measures in composite, and as mentioned, investigate the mechanisms underlying the paradoxical relationship between certain measures and patient outcomes.

In conclusion, this study demonstrates that hospital‐level compliance with the CoC’s Quality of Care Measures is not consistently aligned with patient survival, even for measures that appear to discriminate outcomes at the patient level. We observed one measure for which the compliance definition permits treatment that is discordant with national guidelines, resulting in a paradoxical association between compliance and survival. This underscores a need to move toward quality measures that encompass meaningful measures in processes, outcomes, and patient‐centered care domains while simultaneously reflecting accurate hospital performance. In their current form, the Quality of Care Measures do not uniformly discriminate hospital performance and are limited as a tool for value‐based healthcare delivery.

## Supporting information

Fig S1‐S3‐Table S1‐S12

## Data Availability

The data that support the findings of this study are available from the National Cancer Database. Restrictions apply to the availability of these data, which were used under license for this study. Data are available from the authors with permission from the National Cancer Database.
