# Supplementary material for: Hospital‐level compliance with the commission on cancer’s quality of care measures and the association with patient survival
Source: Cancer Med. 2021 May 4;10(11):3533–44. doi: 10.1002/cam4.3875 (PMC8178497; doi:10.1002/cam4.3875)
Supplement: Supplementary file 1 — Fig S1‐S3‐Table S1‐S12 [file CAM4-10-3533-s001.docx]

Supplementary Appendix

| Figures |  |  | Page |
| --- | --- | --- | --- |
| Figure S1 | Kaplan-Meier estimates of unadjusted patient-level survival for each QCM stratified compliance |  | 2 |
| Figure S2  Figure S3 | Kaplan-Meier estimates of unadjusted hospital-level survival for each QCM stratified by compliance quartile  Kaplan-Meier estimates of unadjusted hospital-level survival for each QCM stratified by EPR group |  | 3  4 |
|  |  |  |  |
| Tables |  |  |  |
| Table S1 | Quartile-based and EPR-based divisions for each QCM |  | 5-6 |
| Table S2 | Patient data for all eligible HT patients |  | 7-10 |
| Table S3 | Patient data for all eligible BCSRT patients |  | 11-14 |
| Table S4 | Patient data for all eligible MASTRT patients |  | 15-18 |
| Table S5 | Patient data for all eligible 12RLN patients |  | 19-21 |
| Table S6 | Patient data for all eligible LNoSurg patients |  | 22-25 |
| Table S7 | Patient data for all eligible LCT patients |  | 26-29 |
| Table S8 | Patient data for all eligible RECRTCT patients |  | 30-32 |
| Table S9 | Patient data for all eligible G15RLN patients |  | 33-35 |
| Table S10 | Unadjusted patient-level overall survival estimates based on compliance |  | 36 |
| Table S11 | Unadjusted hospital-level survival based on quartile group |  | 37 |
| Table S12 | Unadjusted hospital-level survival based on EPR group |  | 38 |
|  |  |  |  |
| Methods |  |  |  |
| Supplementary Methods | Variables included in the multivariable Cox proportional hazards models |  | 39 |


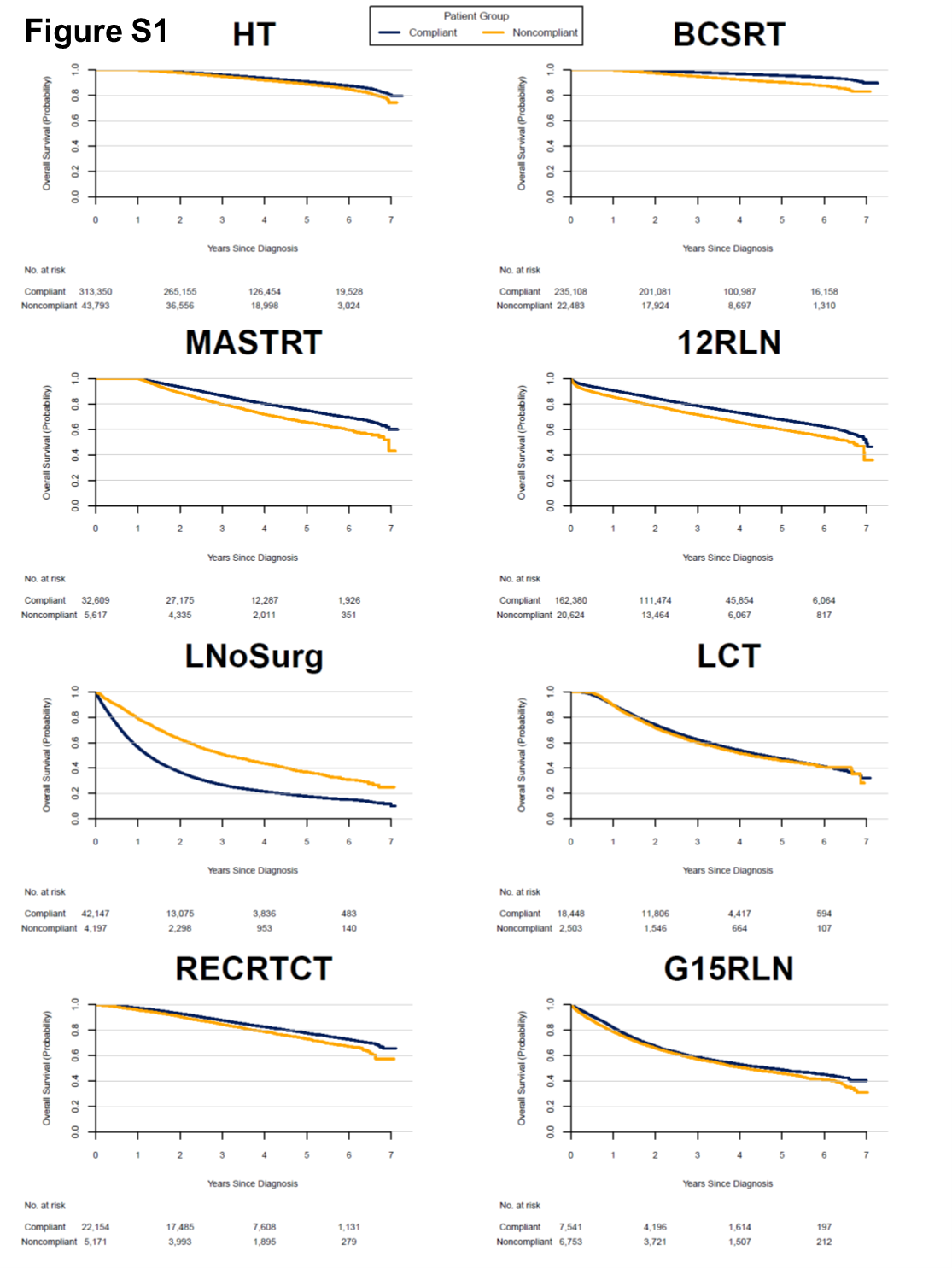

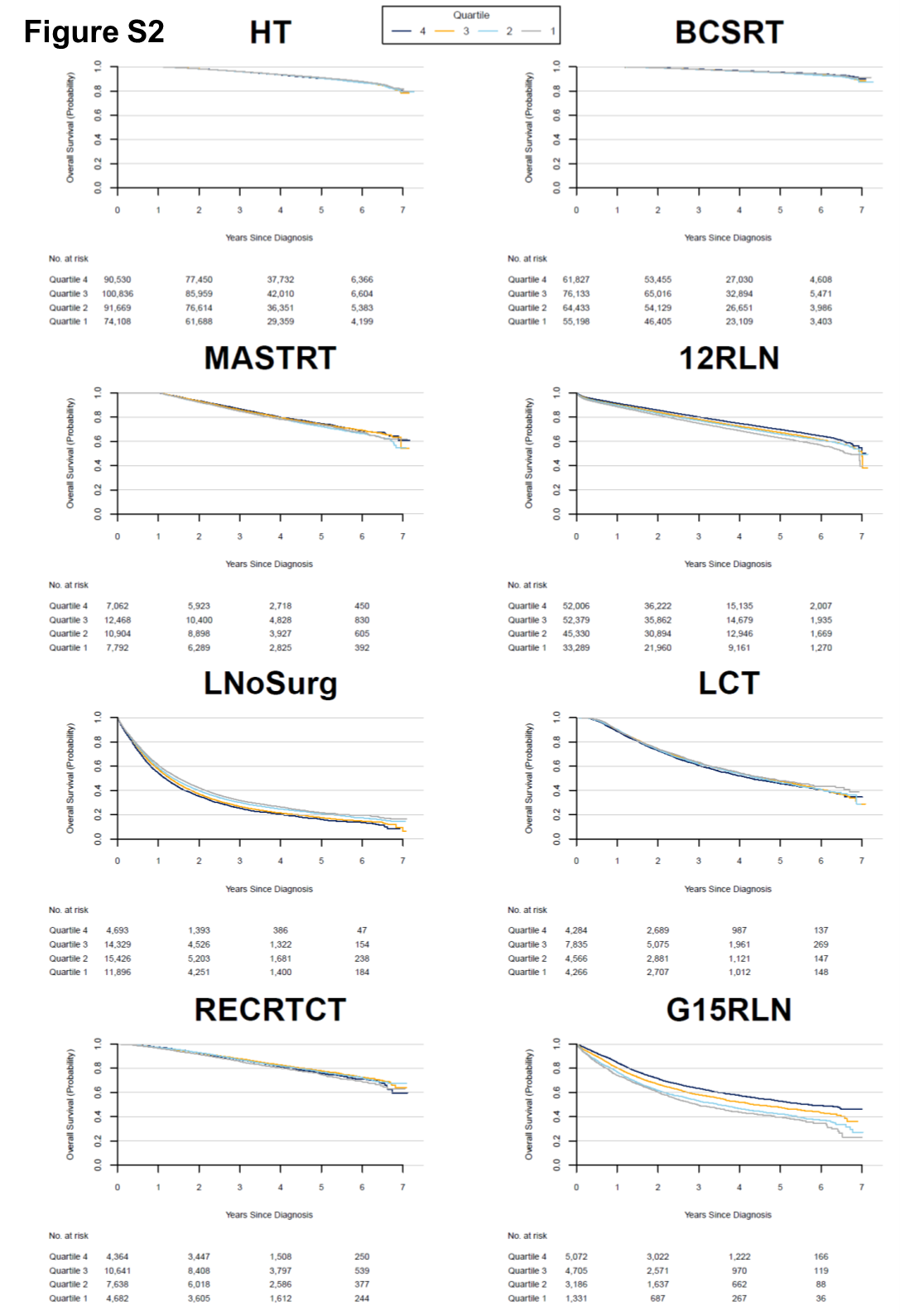

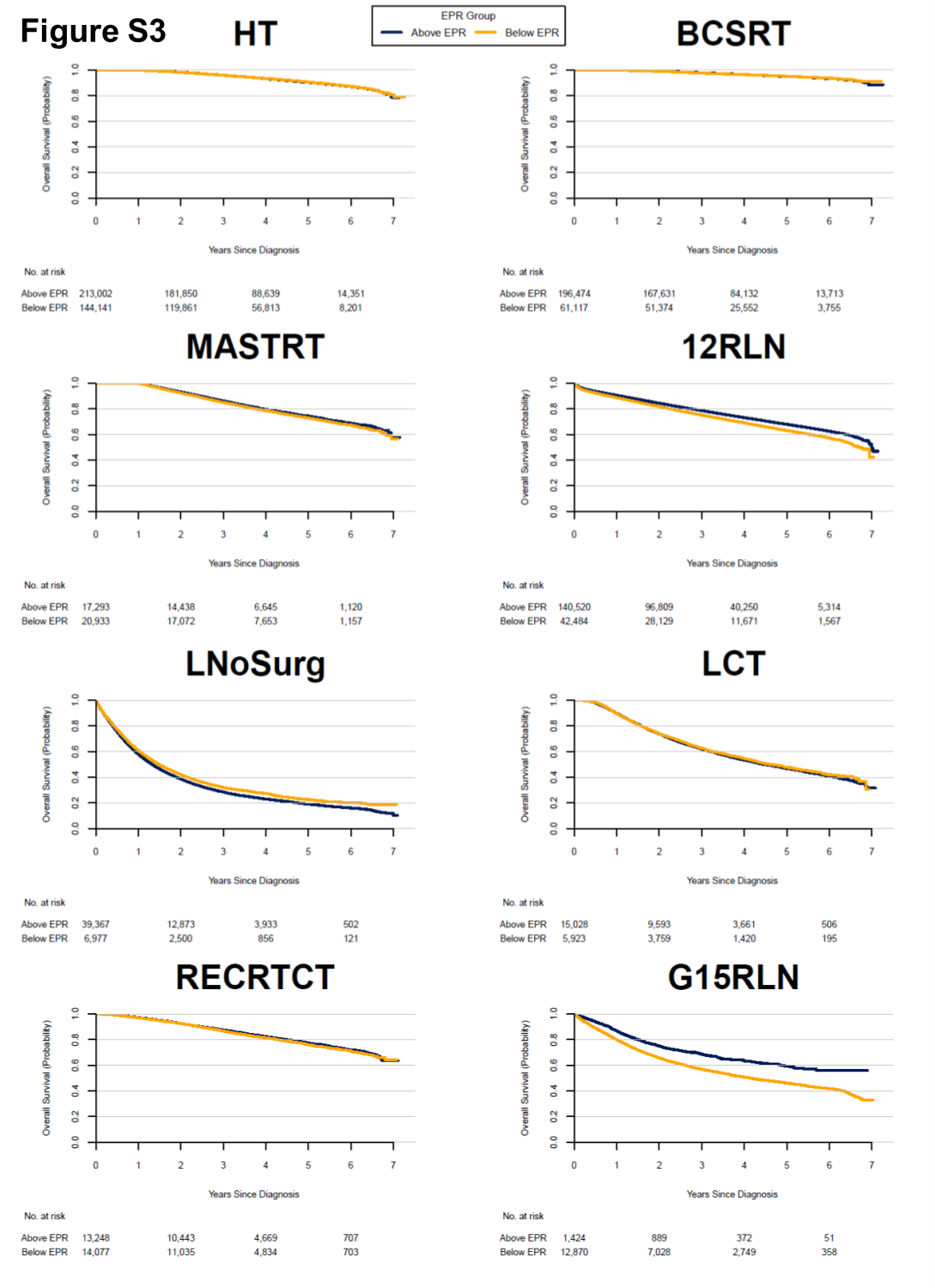


**Figure S1**

Table S1. Quartile-based and EPR-based division for each QCM

| **Measure Name** | **Group (Compliance Range)** | **Number of Patients, %** | | **Number of Hospitals, %** | |
| --- | --- | --- | --- | --- | --- |
| **Breast Cancer** | | | | | |
| BCSRT | Q1 (0 - 89.2%) | 55,200 | 21.4 | 330 | 25 |
|  | Q2 (89.2 - 93.3%) | 64,433 | 25 | 328 | 24.9 |
|  | Q3 (93.3 - 96.0%) | 76,141 | 29.6 | 330 | 25 |
|  | Q4 (96.0 - 100%) | 61,829 | 24 | 330 | 25 |
|  | Below EPR (<90%) | 61,119 | 23.7 | 372 | 28.2 |
|  | Above EPR (>90%) | 196,484 | 76.3 | 946 | 71.8 |
| HT | Q1 (0 - 83.2%) | 74,111 | 20.7 | 329 | 24.9 |
|  | Q2 (83.2 - 91.4%) | 91,671 | 25.7 | 331 | 25.1 |
|  | Q3 (91.4 - 95.4%) | 100,843 | 28.2 | 330 | 25 |
|  | Q4 (95.4 - 100%) | 90,541 | 25.3 | 330 | 25 |
|  | Below EPR (<90%) | 144,146 | 40.4 | 583 | 44.2 |
|  | Above EPR (>90%) | 213,020 | 59.6 | 737 | 55.8 |
| MASTRT | Q1 (0 - 78.6%) | 7,793 | 20.4 | 324 | 24.7 |
|  | Q2 (78.6 - 88.3%) | 10,904 | 28.5 | 331 | 25.2 |
|  | Q3 (88.3 - 95.0%) | 12,468 | 32.6 | 335 | 25.6 |
|  | Q4 (95.0 - 100%) | 7,062 | 18.5 | 321 | 24.5 |
|  | Below EPR (<90%) | 20,934 | 54.8 | 717 | 54.7 |
|  | Above EPR (>90%) | 17,293 | 45.2 | 594 | 45.3 |
| **Colon Cancer** | | | | | |
| 12RLN | Q1 (25.9 - 83.3%) | 33,290 | 18.2 | 327 | 24.9 |
|  | Q2 (83.3 - 89.6%) | 45,345 | 24.8 | 328 | 24.9 |
|  | Q3 (89.6 – 93.8%) | 52,381 | 28.6 | 334 | 25.4 |
|  | Q4 (93.8 - 100%) | 52,010 | 28.4 | 326 | 24.8 |
|  | Below EPR (<85%) | 42,498 | 23.2 | 405 | 30.8 |
|  | Above EPR (>85%) | 140,528 | 76.8 | 910 | 69.2 |
| **Gastric Cancer** | | | | | |
| G15RLN | Q1 (0 - 21.1%) | 1,332 | 9.3 | 289 | 25 |
|  | Q2 (21.1 - 42.9%) | 3,188 | 22.3 | 279 | 24.1 |
|  | Q3 (42.9 - 64.3%) | 4,705 | 32.9 | 299 | 25.8 |
|  | Q4 (64.3 - 100%) | 5,072 | 35.5 | 291 | 25.1 |
|  | Below EPR (<80%) | 12,873 | 90 | 1035 | 89.4 |
|  | Above EPR (>80%) | 1,424 | 10 | 123 | 10.6 |
| **Non-Small Cell Lung Cancer** | | | | | |
| LCT | Q1 (0 - 80.0%) | 4,266 | 20.4 | 285 | 22.9 |
|  | Q2 (80.0 – 88.9%) | 4,566 | 21.8 | 221 | 17.7 |
|  | Q3 (88.9 - 99.9%) | 7,835 | 37.4 | 227 | 18.2 |
|  | Q4 (100%) | 4,285 | 20.5 | 513 | 41.2 |
|  | Below EPR (<85%) | 5,923 | 28.3 | 368 | 29.5 |
|  | Above EPR (>85%) | 15,029 | 71.7 | 878 | 70.5 |
| LNoSurg | Q1 (0 - 87.9%) | 11,896 | 25.7 | 329 | 25.2 |
|  | Q2 (87.9 - 93.0%) | 15,430 | 33.3 | 324 | 24.8 |
|  | Q3 (93.0 - 99.9%) | 14,331 | 30.9 | 318 | 24.4 |
|  | Q4 (99.9 - 100%) | 4,693 | 10.1 | 334 | 25.6 |
|  | Below EPR (<85%) | 6,977 | 15.1 | 217 | 16.6 |
|  | Above EPR (>85%) | 39,373 | 84.9 | 1088 | 83.4 |
| **Rectal Cancer** | | | | | |
| RECRTCT | Q1 (0 - 70.8%) | 4,682 | 17.1 | 321 | 24.9 |
|  | Q2 (70.8 - 83.3%) | 7,638 | 28 | 295 | 22.9 |
|  | Q3 (83.3 - 93.3%) | 10,641 | 38.9 | 340 | 26.4 |
|  | Q4 (93.3 - 100%) | 4,364 | 16 | 333 | 25.8 |
|  | Below EPR (<85%) | 14,077 | 51.5 | 680 | 52.8 |
|  | Above EPR (>85%) | 13,248 | 48.5 | 609 | 47.2 |

Abbreviations: EPR-expected performance rate, QCM-quality of care measure, Q-quartile

Table S2. Patient data for all eligible HT patients

| **Variable** | **Overall** | **Quartile 1** | **Quartile 2** | **Quartile 3** | **Quartile 4** | **Below EPR** | **Above EPR** |
| --- | --- | --- | --- | --- | --- | --- | --- |
| **Demographic variables** | | | | | | | |
| Age, mean (SD) | 60.0 (12.9) | 60.0 (13.1) | 60.0 (13.0) | 60.0 (12.8) | 60.0 (12.9) | 60.0 (13.0) | 60.0 (12.9) |
| Race |  |  |  |  |  |  |  |
| White | 299,873 (83.96) | 57,798 (77.99) | 76,316 (83.25) | 85,920 (85.20) | 79,839 (88.18) | 115,547 (80.16) | 184,326 (86.53) |
| Black | 36,639 (10.26) | 8,631 (11.65) | 9,917 (10.82) | 10,891 (10.80) | 7,200 (7.95) | 16,412 (11.39) | 20,227 (9.50) |
| Other | 17,429 (4.88) | 6,695 (9.03) | 4,498 (4.91) | 3,315 (3.29) | 2,921 (3.23) | 10,378 (7.20) | 7,051 (3.31) |
| Unknown | 3,225 (0.90) | 987 (1.33) | 940 (1.03) | 717 (0.71) | 581 (0.64) | 1,809 (1.25) | 1,416 (0.66) |
| Insurance Status |  |  |  |  |  |  |  |
| Private Insurance | 194,536 (54.47) | 37,350 (50.40) | 50,349 (54.92) | 55,723 (55.26) | 51,114 (56.45) | 75,468 (52.36) | 119,068 (55.90) |
| Medicaid | 120,206 (33.66) | 24,234 (32.70) | 30,632 (33.42) | 34,256 (33.97) | 31,084 (34.33) | 47,576 (33.01) | 72,630 (34.10) |
| Medicare | 25,364 (7.10) | 7,156 (9.66) | 6,477 (7.07) | 6,490 (6.44) | 5,241 (5.79) | 12,325 (8.55) | 13,039 (6.12) |
| Other Government | 3,814 (1.07) | 571 (0.77) | 1,071 (1.17) | 1,318 (1.31) | 854 (0.94) | 1,403 (0.97) | 2,411 (1.13) |
| Not Insured | 7,688 (2.15) | 2,219 (2.99) | 2,021 (2.20) | 2,015 (2.00) | 1,433 (1.58) | 3,882 (2.69) | 3,806 (1.79) |
| Unknown | 5,558 (1.56) | 2,581 (3.48) | 1,121 (1.22) | 1,041 (1.03) | 815 (0.90) | 3,492 (2.42) | 2,066 (0.97) |
| Education (% without high school degree) |  |  |  |  |  |  |  |
| <7% | 101,639 (28.46) | 18,791 (25.36) | 26,387 (28.78) | 29,038 (28.80) | 27,423 (30.29) | 38,159 (26.47) | 63,480 (29.80) |
| 7.0-12.9% | 117,597 (32.93) | 21,010 (28.35) | 30,489 (33.26) | 35,017 (34.72) | 31,081 (34.33) | 43,904 (30.46) | 73,693 (34.59) |
| 13-20.0% | 84,616 (23.69) | 17,007 (22.95) | 21,706 (23.68) | 24,946 (24.74) | 20,957 (23.15) | 34,126 (23.67) | 50,490 (23.70) |
| >=21% | 52,538 (14.71) | 17,099 (23.07) | 12,877 (14.05) | 11,632 (11.53) | 10,930 (12.07) | 27,580 (19.13) | 24,958 (11.72) |
| Missing | 776 (0.22) | 204 (0.28) | 212 (0.23) | 210 (0.21) | 150 (0.17) | 377 (0.26) | 399 (0.19) |
| Charlson Deyo comorbidity score |  |  |  |  |  |  |  |
| 0 | 298,950 (83.70) | 63,598 (85.81) | 77,428 (84.46) | 83,164 (82.47) | 74,760 (82.57) | 122,460 (84.96) | 176,490 (82.85) |
| 1 | 48,086 (13.46) | 8,731 (11.78) | 11,852 (12.93) | 14,564 (14.44) | 12,939 (14.29) | 18,033 (12.51) | 30,053 (14.11) |
| 2 | 8,081 (2.26) | 1,405 (1.90) | 1,927 (2.10) | 2,521 (2.50) | 2,228 (2.46) | 2,895 (2.01) | 5,186 (2.43) |
| >2 | 2,049 (0.57) | 377 (0.51) | 464 (0.51) | 594 (0.59) | 614 (0.68) | 758 (0.53) | 1,291 (0.61) |
| **Cancer variables** | | | | | | | |
| Tumor size (mm), median (IQR) | 20.0 (15.0-29.0) | 20.0 (15.0-30.0) | 20.0 (15.0-30.0) | 19.0 (14.0-28.0) | 19.0 (14.0-28.0) | 20.0 (15.0-30.0) | 19.0 (14.0-28.0) |
| Tumor Grade |  |  |  |  |  |  |  |
| Well differentiated | 71,774 (20.10) | 14,316 (19.32) | 17,674 (19.28) | 21,074 (20.90) | 18,710 (20.66) | 27,806 (19.29) | 43,968 (20.64) |
| Moderately differentiated | 173,932 (48.70) | 36,016 (48.60) | 44,385 (48.42) | 48,852 (48.44) | 44,679 (49.35) | 69,674 (48.34) | 104,258 (48.94) |
| Poorly differentiated | 91,206 (25.54) | 19,616 (26.47) | 23,458 (25.59) | 25,476 (25.26) | 22,656 (25.02) | 37,441 (25.97) | 53,765 (25.24) |
| Undifferentiated | 563 (0.16) | 182 (0.25) | 190 (0.21) | 102 (0.10) | 89 (0.10) | 282 (0.20) | 281 (0.13) |
| Unknown | 19,691 (5.51) | 3,981 (5.37) | 5,964 (6.51) | 5,339 (5.29) | 4,407 (4.87) | 8,943 (6.20) | 10,748 (5.05) |
| Lymph node status |  |  |  |  |  |  |  |
| Negative | 207,354 (58.06) | 42,602 (57.48) | 53,238 (58.08) | 58,523 (58.03) | 52,991 (58.53) | 83,079 (57.64) | 124,275 (58.34) |
| Positive | 143,415 (40.15) | 29,532 (39.85) | 36,875 (40.23) | 40,759 (40.42) | 36,249 (40.04) | 57,868 (40.15) | 85,547 (40.16) |
| Missing | 6,397 (1.79) | 1,977 (2.67) | 1,558 (1.70) | 1,561 (1.55) | 1,301 (1.44) | 3,199 (2.22) | 3,198 (1.50) |
| Estrogen receptor status |  |  |  |  |  |  |  |
| Borderline/uninterpretable | 700 (0.16) | 193 (0.18) | 161 (0.15) | 181 (0.15) | 165 (0.14) | 476 (0.16) | 224 (0.15) |
| Negative/normal | 6,266 (1.39) | 1,377 (1.32) | 1,636 (1.48) | 1,782 (1.47) | 1,471 (1.28) | 4,131 (1.40) | 2,135 (1.38) |
| Positive/elevated | 443,371 (98.43) | 103,043 (98.48) | 108,583 (98.36) | 118,916 (98.36) | 112,829 (98.55) | 291,281 (98.43) | 152,090 (98.45) |
| Test not done | 50 (0.01) | 16 (0.02) | 14 (0.01) | 13 (0.01) | 7 (0.01) | 38 (0.01) | 12 (0.01) |
| Missing | 34 (0.01) | 8 (0.01) | 5 (0.00) | 2 (0.00) | 19 (0.02) | 15 (0.01) | 19 (0.01) |
| Progesterone receptor status |  |  |  |  |  |  |  |
| Borderline/uninterpretable | 492 (0.14) | 161 (0.22) | 129 (0.14) | 110 (0.11) | 92 (0.10) | 265 (0.18) | 227 (0.11) |
| Negative/normal | 44,520 (12.46) | 9,761 (13.17) | 11,479 (12.52) | 12,214 (12.11) | 11,066 (12.22) | 18,594 (12.90) | 25,926 (12.17) |
| Positive/elevated | 311,893 (87.32) | 64,103 (86.50) | 79,976 (87.24) | 88,468 (87.73) | 79,346 (87.64) | 125,137 (86.81) | 186,756 (87.67) |
| Test not done | 84 (0.02) | 24 (0.03) | 23 (0.03) | 21 (0.02) | 16 (0.02) | 41 (0.03) | 43 (0.02) |
| Missing | 177 (0.05) | 62 (0.08) | 64 (0.07) | 30 (0.03) | 21 (0.02) | 109 (0.08) | 68 (0.03) |
| HER2 receptor status |  |  |  |  |  |  |  |
| Borderline/uninterpretable | 7,984 (2.24) | 2,056 (2.77) | 2,140 (2.33) | 2,056 (2.04) | 1,732 (1.91) | 3,607 (2.50) | 4,377 (2.05) |
| Negative/normal | 296,365 (82.98) | 59,654 (80.49) | 75,955 (82.86) | 84,402 (83.70) | 76,354 (84.33) | 117,624 (81.60) | 178,741 (83.91) |
| Positive/elevated | 44,234 (12.38) | 9,013 (12.16) | 11,686 (12.75) | 12,560 (12.46) | 10,975 (12.12) | 17,998 (12.49) | 26,236 (12.32) |
| Test not done | 1,942 (0.54) | 611 (0.82) | 463 (0.51) | 503 (0.50) | 365 (0.40) | 987 (0.68) | 955 (0.45) |
| Missing | 6,641 (1.86) | 2,777 (3.75) | 1,427 (1.56) | 1,322 (1.31) | 1,115 (1.23) | 3,930 (2.73) | 2,711 (1.27) |
| Overall receptor status |  |  |  |  |  |  |  |
| ER positive only | 32,134 (9.00) | 6,745 (9.10) | 8,254 (9.00) | 8,870 (8.80) | 8,265 (9.13) | 13,089 (9.08) | 19,045 (8.94) |
| ER and HER2 positive | 10,013 (2.80) | 2,221 (3.00) | 2,620 (2.86) | 2,733 (2.71) | 2,439 (2.69) | 4,241 (2.94) | 5,772 (2.71) |
| ER and PR positive | 259,359 (72.62) | 52,035 (70.21) | 66,242 (72.26) | 73,652 (73.04) | 67,430 (74.47) | 102,596 (71.18) | 156,763 (73.59) |
| HER2 positive only | 82 (0.02) | 13 (0.02) | 30 (0.03) | 21 (0.02) | 18 (0.02) | 36 (0.02) | 46 (0.02) |
| PR positive only | 3,345 (0.94) | 645 (0.87) | 851 (0.93) | 1,013 (1.00) | 836 (0.92) | 1,285 (0.89) | 2,060 (0.97) |
| PR and HER2 positive | 1,190 (0.33) | 232 (0.31) | 275 (0.30) | 407 (0.40) | 276 (0.30) | 438 (0.30) | 752 (0.35) |
| Triple positive | 32,619 (9.13) | 6,488 (8.75) | 8,653 (9.44) | 9,280 (9.20) | 8,198 (9.05) | 13,127 (9.11) | 19,492 (9.15) |
| Missing | 18,424 (5.16) | 5,732 (7.73) | 4,746 (5.18) | 4,867 (4.83) | 3,079 (3.40) | 9,334 (6.48) | 9,090 (4.27) |
| Overall stage |  |  |  |  |  |  |  |
| 1 | 154,288 (43.20) | 31,396 (42.36) | 38,942 (42.48) | 44,283 (43.91) | 39,667 (43.81) | 60,919 (42.26) | 93,369 (43.83) |
| 2 | 154,765 (43.33) | 32,482 (43.83) | 40,247 (43.90) | 43,053 (42.69) | 38,983 (43.06) | 63,377 (43.97) | 91,388 (42.90) |
| 3 | 48,113 (13.47) | 10,233 (13.81) | 12,482 (13.62) | 13,507 (13.39) | 11,891 (13.13) | 19,850 (13.77) | 28,263 (13.27) |
| **Hospital variables** | | | | | | | |
| Facility type |  |  |  |  |  |  |  |
| Academic/Research Program | 104,462 (29.25) | 24,000 (32.38) | 24,875 (27.14) | 30,772 (30.51) | 24,815 (27.41) | 43,410 (30.12) | 61,052 (28.66) |
| Community Cancer Program | 35,132 (9.84) | 10,260 (13.84) | 9,042 (9.86) | 7,034 (6.98) | 8,796 (9.71) | 17,509 (12.15) | 17,623 (8.27) |
| Comprehensive Community Cancer Program | 160,724 (45.00) | 31,381 (42.34) | 43,558 (47.52) | 46,114 (45.73) | 39,671 (43.82) | 62,617 (43.44) | 98,107 (46.06) |
| Integrated Network Cancer Program | 37,538 (10.51) | 4,470 (6.03) | 9,164 (10.00) | 11,666 (11.57) | 12,238 (13.52) | 12,719 (8.82) | 24,819 (11.65) |
| Missing | 19,310 (5.41) | 4,000 (5.40) | 5,032 (5.49) | 5,257 (5.21) | 5,021 (5.55) | 7,891 (5.47) | 11,419 (5.36) |
| Geographic location |  |  |  |  |  |  |  |
| East North Central | 58,472 (16.37) | 2,587 (3.49) | 14,784 (16.13) | 20,293 (20.12) | 20,808 (22.98) | 13,815 (9.58) | 44,657 (20.96) |
| East South Central | 21,125 (5.91) | 2,603 (3.51) | 5,790 (6.32) | 7,054 (7.00) | 5,678 (6.27) | 6,435 (4.46) | 14,690 (6.90) |
| Middle Atlantic | 50,451 (14.13) | 16,802 (22.67) | 15,621 (17.04) | 8,320 (8.25) | 9,708 (10.72) | 26,946 (18.69) | 23,505 (11.03) |
| Mountain | 16,645 (4.66) | 3,038 (4.10) | 3,095 (3.38) | 7,840 (7.77) | 2,672 (2.95) | 5,120 (3.55) | 11,525 (5.41) |
| New England | 19,182 (5.37) | 2,539 (3.43) | 3,420 (3.73) | 7,301 (7.24) | 5,922 (6.54) | 4,906 (3.40) | 14,276 (6.70) |
| Pacific | 43,429 (12.16) | 21,202 (28.61) | 10,660 (11.63) | 6,143 (6.09) | 5,424 (5.99) | 30,116 (20.89) | 13,313 (6.25) |
| South Atlantic | 74,731 (20.92) | 9,259 (12.49) | 17,531 (19.12) | 26,249 (26.03) | 21,692 (23.96) | 23,117 (16.04) | 51,614 (24.23) |
| West North Central | 25,937 (7.26) | 1,345 (1.81) | 3,924 (4.28) | 9,631 (9.55) | 11,037 (12.19) | 4,944 (3.43) | 20,993 (9.85) |
| West South Central | 27,884 (7.81) | 10,736 (14.49) | 11,814 (12.89) | 2,755 (2.73) | 2,579 (2.85) | 20,856 (14.47) | 7,028 (3.30) |
| Missing | 19,310 (5.41) | 4,000 (5.40) | 5,032 (5.49) | 5,257 (5.21) | 5,021 (5.55) | 7,891 (5.47) | 11,419 (5.36) |
| Number of reporting facilities |  |  |  |  |  |  |  |
| Single | 276,964 (77.54) | 59,938 (80.88) | 71,931 (78.47) | 77,631 (76.98) | 67,464 (74.51) | 115,497 (80.13) | 161,467 (75.80) |
| Multiple | 80,202 (22.46) | 14,173 (19.12) | 19,740 (21.53) | 23,212 (23.02) | 23,077 (25.49) | 28,649 (19.87) | 51,553 (24.20) |
| Travel distance in miles, median (IQR) | 9.1 (4.3-19.0) | 7.6 (3.6-15.2) | 9.0 (4.4-18.5) | 10.3 (4.9-21.6) | 9.6 (4.5-20.1) | 8.1 (3.9-16.3) | 10.0 (4.7-20.9) |
| Number of patients treated, median (IQR) | 406.0 (221.0-665.0) | 330.0 (187.0-578.0) | 424.0 (216.0-662.0) | 425.0 (256.0-756.0) | 433.0 (236.0-684.0) | 366.0 (202.0-630.0) | 431.0 (240.0-689.0) |
| **Treatment variables** | | | | | | | |
| Extent of surgery |  |  |  |  |  |  |  |
| BCS | 192,957 (54.02) | 40,773 (55.02) | 48,693 (53.12) | 53,717 (53.27) | 49,774 (54.97) | 77,598 (53.83) | 115,359 (54.15) |
| Complete mastectomy | 104,975 (29.39) | 20,243 (27.31) | 27,429 (29.92) | 30,542 (30.29) | 26,761 (29.56) | 41,185 (28.57) | 63,790 (29.95) |
| Mastectomy, NOS | 990 (0.28) | 564 (0.76) | 162 (0.18) | 136 (0.13) | 128 (0.14) | 683 (0.47) | 307 (0.14) |
| Modified radical mastectomy | 56,211 (15.74) | 12,022 (16.22) | 14,832 (16.18) | 15,921 (15.79) | 13,436 (14.84) | 23,756 (16.48) | 32,455 (15.24) |
| Radical mastectomy | 1,926 (0.54) | 473 (0.64) | 525 (0.57) | 506 (0.50) | 422 (0.47) | 863 (0.60) | 1,063 (0.50) |
| Missing | 107 (0.03) | 36 (0.05) | 30 (0.03) | 21 (0.02) | 20 (0.02) | 61 (0.04) | 46 (0.02) |
| Use of radiation therapy |  |  |  |  |  |  |  |
| No | 121,375 (33.98) | 26,982 (36.41) | 31,655 (34.53) | 33,716 (33.43) | 29,022 (32.05) | 51,786 (35.93) | 69,589 (32.67) |
| Yes | 234,463 (65.65) | 46,444 (62.67) | 59,628 (65.05) | 66,942 (66.38) | 61,449 (67.87) | 91,341 (63.37) | 143,122 (67.19) |
| Missing | 1,328 (0.37) | 685 (0.92) | 388 (0.42) | 185 (0.18) | 70 (0.08) | 1,019 (0.71) | 309 (0.15) |
| Use of chemotherapy |  |  |  |  |  |  |  |
| No | 179,397 (50.23) | 36,531 (49.29) | 45,681 (49.83) | 50,867 (50.44) | 46,318 (51.16) | 71,274 (49.45) | 108,123 (50.76) |
| Yes | 171,243 (47.94) | 34,688 (46.81) | 44,141 (48.15) | 48,847 (48.44) | 43,567 (48.12) | 68,443 (47.48) | 102,800 (48.26) |
| Missing | 6,526 (1.83) | 2,892 (3.90) | 1,849 (2.02) | 1,129 (1.12) | 656 (0.72) | 4,429 (3.07) | 2,097 (0.98) |
| Use of hormone therapy |  |  |  |  |  |  |  |
| Yes | 318,552 (89.19) | 60,749 (81.97) | 81,024 (88.39) | 92,356 (91.58) | 84,423 (93.24) | 122,063 (84.68) | 196,489 (92.24) |
| No | 33,480 (9.37) | 10,597 (14.30) | 9,276 (10.12) | 7,790 (7.72) | 5,817 (6.42) | 18,158 (12.60) | 15,322 (7.19) |
| Missing | 5,134 (1.44) | 2,765 (3.73) | 1,371 (1.50) | 697 (0.69) | 301 (0.33) | 3,925 (2.72) | 1,209 (0.57) |

Abbreviations: EPR-expected performance rate, SD-standard deviation, mm-millimeter, IQR-interquartile range, ER-estrogen receptor, PR-progesterone receptor, NOS-not otherwise specified

Table S3. Patient data for all eligible BCSRT patients

| **Variable** | **Overall** | **Quartile 1** | **Quartile 2** | **Quartile 3** | **Quartile 4** | **Below EPR** | **Above EPR** |
| --- | --- | --- | --- | --- | --- | --- | --- |
| **Demographic variables** | | | | | | | |
| Age, mean (SD) | 58.0 (8.6) | 57.0 (8.8) | 58.0 (8.6) | 58.0 (8.6) | 58.0 (8.6) | 57.0 (8.8) | 58.0 (8.6) |
| Race |  |  |  |  |  |  |  |
| White | 211,598 (82.14) | 42,086 (76.24) | 52,442 (81.39) | 63,270 (83.10) | 53,800 (87.01) | 46,747 (76.49) | 164,851 (83.90) |
| Black | 31,062 (12.06) | 7,702 (13.95) | 8,708 (13.51) | 9,043 (11.88) | 5,609 (9.07) | 8,714 (14.26) | 22,348 (11.37) |
| Other | 12,344 (4.79) | 4,445 (8.05) | 2,733 (4.24) | 3,201 (4.20) | 1,965 (3.18) | 4,627 (7.57) | 7,717 (3.93) |
| Unknown | 2,599 (1.01) | 967 (1.75) | 550 (0.85) | 627 (0.82) | 455 (0.74) | 1,031 (1.69) | 1,568 (0.80) |
| Insurance Status |  |  |  |  |  |  |  |
| Private Insurance | 172,283 (66.88) | 34,574 (62.63) | 42,448 (65.88) | 51,819 (68.06) | 43,442 (70.26) | 38,216 (62.53) | 134,067 (68.23) |
| Medicaid | 19,049 (7.39) | 6,132 (11.11) | 4,469 (6.94) | 4,994 (6.56) | 3,454 (5.59) | 6,768 (11.07) | 12,281 (6.25) |
| Medicare | 53,392 (20.73) | 11,052 (20.02) | 13,594 (21.10) | 15,868 (20.84) | 12,878 (20.83) | 12,308 (20.14) | 41,084 (20.91) |
| Other Government | 3,130 (1.22) | 544 (0.99) | 859 (1.33) | 1,088 (1.43) | 639 (1.03) | 599 (0.98) | 2,531 (1.29) |
| Not Insured | 5,710 (2.22) | 1,861 (3.37) | 1,441 (2.24) | 1,476 (1.94) | 932 (1.51) | 2,087 (3.41) | 3,623 (1.84) |
| Unknown | 557 (0.22) | 149 (0.27) | 136 (0.21) | 170 (0.22) | 102 (0.16) | 164 (0.27) | 393 (0.20) |
| Education (% without high school degree) |  |  |  |  |  |  |  |
| <7% | 76,185 (29.57) | 13,544 (24.54) | 18,476 (28.67) | 23,371 (30.69) | 20,794 (33.63) | 14,869 (24.33) | 61,316 (31.21) |
| 7.0-12.9% | 84,979 (32.99) | 15,424 (27.94) | 21,367 (33.16) | 26,496 (34.80) | 21,692 (35.08) | 17,518 (28.66) | 67,461 (34.33) |
| 13-20.9% | 59,510 (23.10) | 13,001 (23.55) | 15,762 (24.46) | 17,330 (22.76) | 13,417 (21.70) | 14,636 (23.95) | 44,874 (22.84) |
| >=21% | 36,372 (14.12) | 13,082 (23.70) | 8,692 (13.49) | 8,774 (11.52) | 5,824 (9.42) | 13,932 (22.79) | 22,440 (11.42) |
| Missing | 637 (0.25) | 170 (0.31) | 161 (0.25) | 190 (0.25) | 116 (0.19) | 188 (0.31) | 449 (0.23) |
| Charlson Deyo comorbidity score |  |  |  |  |  |  |  |
| 0 | 222,475 (86.36) | 48,530 (87.92) | 55,994 (86.90) | 64,689 (84.96) | 53,262 (86.14) | 53,477 (87.50) | 168,998 (86.01) |
| 1 | 29,660 (11.51) | 5,657 (10.25) | 7,166 (11.12) | 9,630 (12.65) | 7,207 (11.66) | 6,468 (10.58) | 23,192 (11.80) |
| 2 | 4,478 (1.74) | 829 (1.50) | 1,034 (1.60) | 1,486 (1.95) | 1,129 (1.83) | 960 (1.57) | 3,518 (1.79) |
| >2 | 990 (0.38) | 184 (0.33) | 239 (0.37) | 336 (0.44) | 231 (0.37) | 214 (0.35) | 776 (0.39) |
| **Cancer variables** | | | | | | | |
| Tumor size (mm), median (IQR) | 14.0 (9.0-21.0) | 15.0 (9.0-22.0) | 14.0 (9.0-21.0) | 14.0 (9.0-21.0) | 14.0 (9.0-20.0) | 15.0 (9.0-22.0) | 14.0 (9.0-21.0) |
| Tumor Grade |  |  |  |  |  |  |  |
| Well differentiated | 64,668 (25.10) | 13,027 (23.60) | 16,411 (25.47) | 19,129 (25.12) | 16,101 (26.04) | 14,502 (23.73) | 50,166 (25.53) |
| Moderately differentiated | 104,678 (40.64) | 22,455 (40.68) | 25,793 (40.03) | 31,185 (40.96) | 25,245 (40.83) | 24,878 (40.70) | 79,800 (40.61) |
| Poorly differentiated | 71,798 (27.87) | 15,798 (28.62) | 17,856 (27.71) | 21,164 (27.80) | 16,980 (27.46) | 17,410 (28.49) | 54,388 (27.68) |
| Undifferentiated | 498 (0.19) | 145 (0.26) | 146 (0.23) | 85 (0.11) | 122 (0.20) | 153 (0.25) | 345 (0.18) |
| Unknown | 15,961 (6.20) | 3,775 (6.84) | 4,227 (6.56) | 4,578 (6.01) | 3,381 (5.47) | 4,176 (6.83) | 11,785 (6.00) |
| Lymph node status |  |  |  |  |  |  |  |
| Negative | 196,014 (76.09) | 41,021 (74.31) | 48,999 (76.05) | 58,177 (76.41) | 47,817 (77.34) | 45,492 (74.43) | 150,522 (76.61) |
| Positive | 54,495 (21.15) | 12,045 (21.82) | 13,535 (21.01) | 16,058 (21.09) | 12,857 (20.79) | 13,327 (21.81) | 41,168 (20.95) |
| Missing | 7,094 (2.75) | 2,134 (3.87) | 1,899 (2.95) | 1,906 (2.50) | 1,155 (1.87) | 2,300 (3.76) | 4,794 (2.44) |
| Estrogen receptor status |  |  |  |  |  |  |  |
| Borderline/uninterpretable | 170 (0.07) | 54 (0.10) | 37 (0.06) | 51 (0.07) | 28 (0.05) | 57 (0.09) | 113 (0.06) |
| Negative/normal | 41,432 (16.08) | 8,921 (16.16) | 10,532 (16.35) | 12,366 (16.24) | 9,613 (15.55) | 9,960 (16.30) | 31,472 (16.02) |
| Positive/elevated | 214,317 (83.20) | 45,300 (82.07) | 53,522 (83.07) | 63,464 (83.35) | 52,031 (84.15) | 50,128 (82.02) | 164,189 (83.56) |
| Test not done | 413 (0.16) | 155 (0.28) | 80 (0.12) | 109 (0.14) | 69 (0.11) | 164 (0.27) | 249 (0.13) |
| Missing | 1,271 (0.49) | 770 (1.39) | 262 (0.41) | 151 (0.20) | 88 (0.14) | 810 (1.33) | 461 (0.23) |
| Progesterone receptor status |  |  |  |  |  |  |  |
| Borderline/uninterpretable | 382 (0.15) | 105 (0.19) | 83 (0.13) | 107 (0.14) | 87 (0.14) | 118 (0.19) | 264 (0.13) |
| Negative/normal | 63,183 (24.53) | 13,588 (24.62) | 15,946 (24.75) | 18,826 (24.73) | 14,823 (23.97) | 15,126 (24.75) | 48,057 (24.46) |
| Positive/elevated | 192,061 (74.56) | 40,509 (73.39) | 48,012 (74.51) | 56,854 (74.67) | 46,686 (75.51) | 44,821 (73.33) | 147,240 (74.94) |
| Test not done | 570 (0.22) | 185 (0.34) | 96 (0.15) | 162 (0.21) | 127 (0.21) | 197 (0.32) | 373 (0.19) |
| Missing | 1,407 (0.55) | 813 (1.47) | 296 (0.46) | 192 (0.25) | 106 (0.17) | 857 (1.40) | 550 (0.28) |
| HER2 receptor status |  |  |  |  |  |  |  |
| Borderline/uninterpretable | 4,824 (1.87) | 1,139 (2.06) | 1,206 (1.87) | 1,556 (2.04) | 923 (1.49) | 1,223 (2.00) | 3,601 (1.83) |
| Negative/normal | 212,335 (82.43) | 44,273 (80.20) | 52,638 (81.69) | 63,246 (83.06) | 52,178 (84.39) | 49,205 (80.51) | 163,130 (83.02) |
| Positive/elevated | 30,817 (11.96) | 6,869 (12.44) | 7,629 (11.84) | 9,121 (11.98) | 7,198 (11.64) | 7,572 (12.39) | 23,245 (11.83) |
| Test not done | 3,717 (1.44) | 1,167 (2.11) | 744 (1.15) | 1,038 (1.36) | 768 (1.24) | 1,241 (2.03) | 2,476 (1.26) |
| Missing | 5,910 (2.29) | 1,752 (3.17) | 2,216 (3.44) | 1,180 (1.55) | 762 (1.23) | 1,878 (3.07) | 4,032 (2.05) |
| Overall receptor status |  |  |  |  |  |  |  |
| ER positive only | 17,811 (6.91) | 3,737 (6.77) | 4,396 (6.82) | 5,336 (7.01) | 4,342 (7.02) | 4,152 (6.79) | 13,659 (6.95) |
| ER and HER2 positive | 5,019 (1.95) | 1,094 (1.98) | 1,277 (1.98) | 1,461 (1.92) | 1,187 (1.92) | 1,221 (2.00) | 3,798 (1.93) |
| ER and PR positive | 162,694 (63.16) | 33,828 (61.28) | 40,291 (62.53) | 48,351 (63.50) | 40,224 (65.06) | 37,535 (61.41) | 125,159 (63.70) |
| HER2 positive only | 8,114 (3.15) | 1,858 (3.37) | 1,962 (3.05) | 2,426 (3.19) | 1,868 (3.02) | 2,055 (3.36) | 6,059 (3.08) |
| PR positive only | 1,889 (0.73) | 388 (0.70) | 501 (0.78) | 574 (0.75) | 426 (0.69) | 428 (0.70) | 1,461 (0.74) |
| PR and HER2 positive | 544 (0.21) | 126 (0.23) | 150 (0.23) | 147 (0.19) | 121 (0.20) | 138 (0.23) | 406 (0.21) |
| Triple negative | 28,923 (11.23) | 6,137 (11.12) | 7,340 (11.39) | 8,643 (11.35) | 6,803 (11.00) | 6,890 (11.27) | 22,033 (11.21) |
| Triple positive | 16,973 (6.59) | 3,755 (6.80) | 4,275 (6.63) | 4,994 (6.56) | 3,949 (6.39) | 4,120 (6.74) | 12,853 (6.54) |
| Missing | 15,636 (6.07) | 4,277 (7.75) | 4,241 (6.58) | 4,209 (5.53) | 2,909 (4.70) | 4,580 (7.49) | 11,056 (5.63) |
| Overall stage |  |  |  |  |  |  |  |
| 1 | 172,100 (66.81) | 35,843 (64.93) | 42,859 (66.52) | 51,125 (67.15) | 42,273 (68.37) | 39,817 (65.15) | 132,283 (67.33) |
| 2 | 74,675 (28.99) | 16,813 (30.46) | 18,882 (29.30) | 21,808 (28.64) | 17,172 (27.77) | 18,511 (30.29) | 56,164 (28.58) |
| 3 | 10,828 (4.20) | 2,544 (4.61) | 2,692 (4.18) | 3,208 (4.21) | 2,384 (3.86) | 2,791 (4.57) | 8,037 (4.09) |
| **Hospital variables** | | | | | | | |
| Facility type |  |  |  |  |  |  |  |
| Academic/Research Program | 79,781 (30.97) | 18,935 (34.30) | 21,068 (32.70) | 24,505 (32.18) | 15,273 (24.70) | 20,691 (33.85) | 59,090 (30.07) |
| Community Cancer Program | 25,421 (9.87) | 6,980 (12.64) | 6,340 (9.84) | 5,267 (6.92) | 6,834 (11.05) | 7,810 (12.78) | 17,611 (8.96) |
| Comprehensive Community Cancer Program | 116,408 (45.19) | 22,329 (40.45) | 29,681 (46.06) | 35,806 (47.03) | 28,592 (46.24) | 25,477 (41.68) | 90,931 (46.28) |
| Integrated Network Cancer Program | 27,112 (10.52) | 4,783 (8.66) | 5,147 (7.99) | 8,071 (10.60) | 9,111 (14.74) | 4,783 (7.83) | 22,329 (11.36) |
| Missing | 8,881 (3.45) | 2,173 (3.94) | 2,197 (3.41) | 2,492 (3.27) | 2,019 (3.27) | 2,358 (3.86) | 6,523 (3.32) |
| Geographic location |  |  |  |  |  |  |  |
| East North Central | 44,148 (17.14) | 2,188 (3.96) | 11,696 (18.15) | 14,054 (18.46) | 16,210 (26.22) | 3,168 (5.18) | 40,980 (20.86) |
| East South Central | 13,495 (5.24) | 2,561 (4.64) | 5,061 (7.85) | 2,954 (3.88) | 2,919 (4.72) | 2,935 (4.80) | 10,560 (5.37) |
| Middle Atlantic | 40,815 (15.84) | 13,096 (23.72) | 6,325 (9.82) | 11,831 (15.54) | 9,563 (15.47) | 14,267 (23.34) | 26,548 (13.51) |
| Mountain | 11,966 (4.65) | 2,070 (3.75) | 3,989 (6.19) | 3,682 (4.84) | 2,225 (3.60) | 2,155 (3.53) | 9,811 (4.99) |
| New England | 17,661 (6.86) | 2,011 (3.64) | 2,513 (3.90) | 8,557 (11.24) | 4,580 (7.41) | 2,283 (3.74) | 15,378 (7.83) |
| Pacific | 31,181 (12.10) | 13,847 (25.09) | 6,590 (10.23) | 7,820 (10.27) | 2,924 (4.73) | 14,710 (24.07) | 16,471 (8.38) |
| South Atlantic | 54,040 (20.98) | 7,075 (12.82) | 17,933 (27.83) | 18,109 (23.78) | 10,923 (17.67) | 8,268 (13.53) | 45,772 (23.30) |
| West North Central | 17,449 (6.77) | 542 (0.98) | 4,128 (6.41) | 3,143 (4.13) | 9,636 (15.58) | 850 (1.39) | 16,599 (8.45) |
| West South Central | 17,967 (6.97) | 9,637 (17.46) | 4,001 (6.21) | 3,499 (4.60) | 830 (1.34) | 10,125 (16.57) | 7,842 (3.99) |
| Missing | 8,881 (3.45) | 2,173 (3.94) | 2,197 (3.41) | 2,492 (3.27) | 2,019 (3.27) | 2,358 (3.86) | 6,523 (3.32) |
| Number of reporting facilities |  |  |  |  |  |  |  |
| Single | 194,603 (75.54) | 43,619 (79.02) | 49,952 (77.53) | 56,111 (73.69) | 44,921 (72.65) | 48,353 (79.11) | 146,250 (74.43) |
| Multiple | 63,000 (24.46) | 11,581 (20.98) | 14,481 (22.47) | 20,030 (26.31) | 16,908 (27.35) | 12,766 (20.89) | 50,234 (25.57) |
| Travel distance in miles, median (IQR) | 8.9 (4.3-18.0) | 7.6 (3.7-15.1) | 9.2 (4.5-19.2) | 9.5 (4.6-18.5) | 9.3 (4.5-19.0) | 7.7 (3.7-15.3) | 9.4,(4.5-18.9) |
| Number of patients treated, median (IQR) | 296 (168.0-500.0) | 274.0 (141.0-456.0) | 292.0 (159.0-492.0) | 330.0 (196.0-551.0) | 288.0 (168.0-450.0) | 257.0 (137.0-426.0) | 306.0 (176.0-505.0) |
| **Treatment variables** | | | | | | | |
| Extent of Surgery |  |  |  |  |  |  |  |
| BCS | 257,603 (100.00) | 55,200 (100.00) | 64,433 (100.00) | 76,141 (100.00) | 61,829 (100.00) | 61,119 (100.00) | 196,484 (100.00) |
| Use of radiation therapy |  |  |  |  |  |  |  |
| No | 15,477 (6.01) | 6,774 (12.27) | 4,080 (6.33) | 3,212 (4.22) | 1,411 (2.28) | 7,217 (11.81) | 8,260 (4.20) |
| Yes | 241,690 (93.82) | 48,148 (87.22) | 60,256 (93.52) | 72,885 (95.72) | 60,401 (97.69) | 53,601 (87.70) | 188,089 (95.73) |
| Missing | 436 (0.17) | 278 (0.50) | 97 (0.15) | 44 (0.06) | 17 (0.03) | 301 (0.49) | 135 (0.07) |
| Use of chemotherapy |  |  |  |  |  |  |  |
| No | 143,012 (55.52) | 29,643 (53.70) | 35,724 (55.44) | 42,615 (55.97) | 35,030 (56.66) | 32,857 (53.76) | 110,155 (56.06) |
| Yes | 109,875 (42.65) | 23,254 (42.13) | 27,603 (42.84) | 32,736 (42.99) | 26,282 (42.51) | 25,813 (42.23) | 84,062 (42.78) |
| Missing | 4,716 (1.83) | 2,303 (4.17) | 1,106 (1.72) | 790 (1.04) | 517 (0.84) | 2,449 (4.01) | 2,267 (1.15) |
| Use of hormone therapy |  |  |  |  |  |  |  |
| Yes | 191,941 (74.51) | 36,918 (66.88) | 47,745 (74.10) | 58,367 (76.66) | 48,911 (79.11) | 41,166 (67.35) | 150,775 (76.74) |
| No | 59,638 (23.15) | 15,542 (28.16) | 15,177 (23.55) | 16,665 (21.89) | 12,254 (19.82) | 16,956 (27.74) | 42,682 (21.72) |
| Missing | 6,024 (2.34) | 2,740 (4.96) | 1,511 (2.35) | 1,109 (1.46) | 664 (1.07) | 2,997 (4.90) | 3,027 (1.54) |

Abbreviations: EPR-expected performance rate, SD-standard deviation, mm-millimeter, IQR-interquartile range, ER-estrogen receptor, PR-progesterone receptor

Table S4. Patient data for all eligible MASTRT patients

| **Variable** | **Overall** | **Quartile 1** | **Quartile 2** | **Quartile 3** | **Quartile 4** | **Below EPR** | **Above EPR** |
| --- | --- | --- | --- | --- | --- | --- | --- |
| **Demographic variables** | | | | | | | |
| Age, mean (SD) | 56.0 (13.5) | 57.0 (13.7) | 56.0 (13.5) | 56.0 (13.4) | 56.0 (13.5) | 56.0 (13.5) | 56.0 (13.5) |
| Race |  |  |  |  |  |  |  |
| White | 336 (0.88) | 120 (1.54) | 60 (0.55) | 103 (0.83) | 53 (0.75) | 206 (0.98) | 130 (0.75) |
| Black | 5,232 (13.69) | 1,248 (16.01) | 1,689 (15.49) | 1,498 (12.01) | 797 (11.29) | 3,187 (15.22) | 2,045 (11.83) |
| Other | 1,957 (5.12) | 556 (7.13) | 566 (5.19) | 528 (4.23) | 307 (4.35) | 1,243 (5.94) | 714 (4.13) |
| Unknown | 336 (0.88) | 120 (1.54) | 60 (0.55) | 103 (0.83) | 53 (0.75) | 206 (0.98) | 130 (0.75) |
| Insurance Status |  |  |  |  |  |  |  |
| Private Insurance | 20,808 (54.43) | 3,572 (45.84) | 5,901 (54.12) | 7,241 (58.08) | 4,094 (57.97) | 10,730 (51.26) | 10,078 (58.28) |
| Medicaid | 4,436 (11.60) | 1,274 (16.35) | 1,249 (11.45) | 1,269 (10.18) | 644 (9.12) | 2,780 (13.28) | 1,656 (9.58) |
| Medicare | 10,664 (27.90) | 2,275 (29.19) | 3,033 (27.82) | 3,405 (27.31) | 1,951 (27.63) | 5,943 (28.39) | 4,721 (27.30) |
| Other Government | 492 (1.29) | 62 (0.80) | 168 (1.54) | 165 (1.32) | 97 (1.37) | 254 (1.21) | 238 (1.38) |
| Not Insured | 1,330 (3.48) | 443 (5.68) | 403 (3.70) | 277 (2.22) | 207 (2.93) | 894 (4.27) | 436 (2.52) |
| Unknown | 497 (1.30) | 167 (2.14) | 150 (1.38) | 111 (0.89) | 69 (0.98) | 333 (1.59) | 164 (0.95) |
| Education (% without high school degree) |  |  |  |  |  |  |  |
| <7% | 9,255 (24.21) | 1,331 (17.08) | 2,380 (21.83) | 3,657 (29.33) | 1,887 (26.72) | 4,331 (20.69) | 4,924 (28.47) |
| 7.0-12.9% | 12,093 (31.63) | 2,036 (26.13) | 3,456 (31.69) | 4,161 (33.37) | 2,440 (34.55) | 6,212 (29.67) | 5,881 (34.01) |
| 13-20.9% | 9,848 (25.76) | 2,035 (26.11) | 3,032 (27.81) | 3,016 (24.19) | 1,765 (24.99) | 5,644 (26.96) | 4,204 (24.31) |
| >=21% | 6,917 (18.09) | 2,367 (30.37) | 2,001 (18.35) | 1,601 (12.84) | 948 (13.42) | 4,684 (22.38) | 2,233 (12.91) |
| Missing | 114 (0.30) | 24 (0.31) | 35 (0.32) | 33 (0.26) | 22 (0.31) | 63 (0.30) | 51 (0.29) |
| Charlson Deyo comorbidity score |  |  |  |  |  |  |  |
| 0 | 31,684 (82.88) | 6,519 (83.65) | 9,057 (83.06) | 10,322 (82.79) | 5,786 (81.93) | 17,442 (83.32) | 14,242 (82.36) |
| 1 | 5,345 (13.98) | 1,043 (13.38) | 1,500 (13.76) | 1,757 (14.09) | 1,045 (14.80) | 2,846 (13.60) | 2,499 (14.45) |
| 2 | 926 (2.42) | 176 (2.26) | 262 (2.40) | 309 (2.48) | 179 (2.53) | 491 (2.35) | 435 (2.52) |
| >2 | 272 (0.71) | 55 (0.71) | 85 (0.78) | 80 (0.64) | 52 (0.74) | 155 (0.74) | 117 (0.68) |
| **Cancer variables** | | | | | | | |
| Tumor size (mm), median (IQR) | 36.0 (25.0-56.0) | 38.0 (25.0-58.0) | 36.0 (25.0-57.0) | 36.0 (25.0-57.0) | 35.0 (24.0-55.0) | 36.0 (25.0-57.0) | 35.0 (24.0-56.0) |
| Tumor Grade |  |  |  |  |  |  |  |
| Well differentiated | 2,893 (7.57) | 538 (6.90) | 866 (7.94) | 933 (7.48) | 556 (7.87) | 1,585 (7.57) | 1,308 (7.56) |
| Moderately differentiated | 15,966 (41.77) | 3,174 (40.73) | 4,466 (40.96) | 5,317 (42.65) | 3,009 (42.61) | 8,539 (40.79) | 7,427 (42.95) |
| Poorly differentiated | 16,674 (43.62) | 3,472 (44.55) | 4,793 (43.96) | 5,446 (43.68) | 2,963 (41.96) | 9,259 (44.23) | 7,415 (42.88) |
| Undifferentiated | 114 (0.30) | 35 (0.45) | 30 (0.28) | 30 (0.24) | 19 (0.27) | 70 (0.33) | 44 (0.25) |
| Unknown | 2,580 (6.75) | 574 (7.37) | 749 (6.87) | 742 (5.95) | 515 (7.29) | 1,481 (7.07) | 1,099 (6.36) |
| Lymph node status |  |  |  |  |  |  |  |
| Positive | 38,227 (100.00) | 7,793 (100.00) | 10,904 (100.00) | 12,468 (100.00) | 7,062 (100.00) | 20,934 (100.00) | 17,293 (100.00) |
| Estrogen receptor status |  |  |  |  |  |  |  |
| Borderline/uninterpretable | 19 (0.05) | 5 (0.06) | 3 (0.03) | 6 (0.05) | 5 (0.07) | 8 (0.04) | 11 (0.06) |
| Negative/normal | 7,691 (20.12) | 1,627 (20.88) | 2,175 (19.95) | 2,492 (19.99) | 1,397 (19.78) | 4,269 (20.39) | 3,422 (19.79) |
| Positive/elevated | 30,279 (79.21) | 6,006 (77.07) | 8,683 (79.63) | 9,938 (79.71) | 5,652 (80.03) | 16,447 (78.57) | 13,832 (79.99) |
| Test not done | 35 (0.09) | 16 (0.21) | 8 (0.07) | 10 (0.08) | 1 (0.01) | 26 (0.12) | 9 (0.05) |
| Missing | 203 (0.53) | 139 (1.78) | 35 (0.32) | 22 (0.18) | 7 (0.10) | 184 (0.88) | 19 (0.11) |
| Progesterone receptor status |  |  |  |  |  |  |  |
| Borderline/uninterpretable | 60 (0.16) | 15 (0.19) | 17 (0.16) | 20 (0.16) | 8 (0.11) | 33 (0.16) | 27 (0.16) |
| Negative/normal | 11,713 (30.64) | 2,468 (31.67) | 3,320 (30.45) | 3,784 (30.35) | 2,141 (30.32) | 6,480 (30.95) | 5,233 (30.26) |
| Positive/elevated | 26,188 (68.51) | 5,149 (66.07) | 7,515 (68.92) | 8,626 (69.19) | 4,898 (69.36) | 14,193 (67.80) | 11,995 (69.36) |
| Test not done | 45 (0.12) | 20 (0.26) | 11 (0.10) | 11 (0.09) | 3 (0.04) | 33 (0.16) | 12 (0.07) |
| Missing | 221 (0.58) | 141 (1.81) | 41 (0.38) | 27 (0.22) | 12 (0.17) | 195 (0.93) | 26 (0.15) |
| HER2 receptor status |  |  |  |  |  |  |  |
| Borderline/uninterpretable | 868 (2.27) | 232 (2.98) | 288 (2.64) | 232 (1.86) | 116 (1.64) | 562 (2.68) | 306 (1.77) |
| Negative/normal | 28,995 (75.85) | 5,635 (72.31) | 8,207 (75.27) | 9,703 (77.82) | 5,450 (77.17) | 15,565 (74.35) | 13,430 (77.66) |
| Positive/elevated | 7,360 (19.25) | 1,517 (19.47) | 2,128 (19.52) | 2,342 (18.78) | 1,373 (19.44) | 4,062 (19.40) | 3,298 (19.07) |
| Test not done | 225 (0.59) | 88 (1.13) | 58 (0.53) | 46 (0.37) | 33 (0.47) | 154 (0.74) | 71 (0.41) |
| Missing | 779 (2.04) | 321 (4.12) | 223 (2.05) | 145 (1.16) | 90 (1.27) | 591 (2.82) | 188 (1.09) |
| Overall receptor status |  |  |  |  |  |  |  |
| ER positive only | 3,057 (8.00) | 623 (7.99) | 866 (7.94) | 1,007 (8.08) | 561 (7.94) | 1,666 (7.96) | 1,391 (8.04) |
| ER and HER2 positive | 1,172 (3.07) | 234 (3.00) | 341 (3.13) | 364 (2.92) | 233 (3.30) | 627 (3.00) | 545 (3.15) |
| ER and PR positive | 21,039 (55.04) | 4,017 (51.55) | 5,989 (54.92) | 7,040 (56.46) | 3,993 (56.54) | 11,258 (53.78) | 9,781 (56.56) |
| HER2 positive only | 2,434 (6.37) | 527 (6.76) | 708 (6.49) | 751 (6.02) | 448 (6.34) | 1,378 (6.58) | 1,056 (6.11) |
| PR positive only | 236 (0.62) | 38 (0.49) | 62 (0.57) | 82 (0.66) | 54 (0.76) | 112 (0.54) | 124 (0.72) |
| PR and HER2 positive | 163 (0.43) | 35 (0.45) | 42 (0.39) | 64 (0.51) | 22 (0.31) | 89 (0.43) | 74 (0.43) |
| Triple negative | 4,564 (11.94) | 946 (12.14) | 1,281 (11.75) | 1,510 (12.11) | 827 (11.71) | 2,499 (11.94) | 2,065 (11.94) |
| Triple positive | 3,561 (9.32) | 725 (9.30) | 1,051 (9.64) | 1,133 (9.09) | 652 (9.23) | 1,973 (9.42) | 1,588 (9.18) |
| Missing | 2,001 (5.23) | 648 (8.32) | 564 (5.17) | 517 (4.15) | 272 (3.85) | 1,332 (6.36) | 669 (3.87) |
| Overall stage |  |  |  |  |  |  |  |
| 0 | 229 (0.60) | 37 (0.47) | 55 (0.50) | 94 (0.75) | 43 (0.61) | 104 (0.50) | 125 (0.72) |
| 1 | 480 (1.26) | 102 (1.31) | 156 (1.43) | 161 (1.29) | 61 (0.86) | 281 (1.34) | 199 (1.15) |
| 2 | 2,001 (5.23) | 468 (6.01) | 611 (5.60) | 613 (4.92) | 309 (4.38) | 1,193 (5.70) | 808 (4.67) |
| 3 | 35,517 (92.91) | 7,186 (92.21) | 10,082 (92.46) | 11,600 (93.04) | 6,649 (94.15) | 19,356 (92.46) | 16,161 (93.45) |
| **Hospital variables** | | | | | | | |
| Facility type |  |  |  |  |  |  |  |
| Academic/Research Program | 11,044 (28.89) | 2,442 (31.34) | 3,231 (29.63) | 3,512 (28.17) | 1,859 (26.32) | 6,243 (29.82) | 4,801 (27.76) |
| Community Cancer Program | 3,683 (9.63) | 1,075 (13.79) | 874 (8.02) | 709 (5.69) | 1,025 (14.51) | 2,011 (9.61) | 1,672 (9.67) |
| Comprehensive Community Cancer Program | 16,152 (42.25) | 2,996 (38.44) | 4,830 (44.30) | 5,592 (44.85) | 2,734 (38.71) | 8,891 (42.47) | 7,261 (41.99) |
| Integrated Network Cancer Program | 3,649 (9.55) | 528 (6.78) | 915 (8.39) | 1,458 (11.69) | 748 (10.59) | 1,781 (8.51) | 1,868 (10.80) |
| Missing | 3,699 (9.68) | 752 (9.65) | 1,054 (9.67) | 1,197 (9.60) | 696 (9.86) | 2,008 (9.59) | 1,691 (9.78) |
| Geographic location |  |  |  |  |  |  |  |
| East North Central | 5,821 (15.23) | 524 (6.72) | 1,584 (14.53) | 2,271 (18.21) | 1,442 (20.42) | 2,466 (11.78) | 3,355 (19.40) |
| East South Central | 2,626 (6.87) | 588 (7.55) | 1,066 (9.78) | 604 (4.84) | 368 (5.21) | 1,747 (8.35) | 879 (5.08) |
| Middle Atlantic | 4,866 (12.73) | 1,217 (15.62) | 1,077 (9.88) | 1,496 (12.00) | 1,076 (15.24) | 2,595 (12.40) | 2,271 (13.13) |
| Mountain | 1,616 (4.23) | 139 (1.78) | 535 (4.91) | 740 (5.94) | 202 (2.86) | 772 (3.69) | 844 (4.88) |
| New England | 1,435 (3.75) | 137 (1.76) | 218 (2.00) | 650 (5.21) | 430 (6.09) | 437 (2.09) | 998 (5.77) |
| Pacific | 4,375 (11.44) | 1,658 (21.28) | 1,114 (10.22) | 1,090 (8.74) | 513 (7.26) | 3,088 (14.75) | 1,287 (7.44) |
| South Atlantic | 7,765 (20.31) | 1,243 (15.95) | 2,346 (21.52) | 2,657 (21.31) | 1,519 (21.51) | 4,199 (20.06) | 3,566 (20.62) |
| West North Central | 2,747 (7.19) | 106 (1.36) | 689 (6.32) | 1,265 (10.15) | 687 (9.73) | 874 (4.18) | 1,873 (10.83) |
| West South Central | 3,277 (8.57) | 1,429 (18.34) | 1,221 (11.20) | 498 (3.99) | 129 (1.83) | 2,748 (13.13) | 529 (3.06) |
| Missing | 3,699 (9.68) | 752 (9.65) | 1,054 (9.67) | 1,197 (9.60) | 696 (9.86) | 2,008 (9.59) | 1,691 (9.78) |
| Number of reporting facilities |  |  |  |  |  |  |  |
| Single | 28,926 (75.67) | 6,144 (78.84) | 8,351 (76.59) | 9,141 (73.32) | 5,290 (74.91) | 16,176 (77.27) | 12,750 (73.73) |
| Multiple | 9,301 (24.33) | 1,649 (21.16) | 2,553 (23.41) | 3,327 (26.68) | 1,772 (25.09) | 4,758 (22.73) | 4,543 (26.27) |
| Travel distance in miles, median (IQR) | 9.6 (4.4-20.5) | 8.2 (3.8-16.8) | 9.6 (4.5-20.6) | 10.4 (4.8-21.6) | 9.9 (4.6-22.5) | 9.0 (4.2-19.0) | 10.3 (4.7-22.2) |
| Number of patients treated, median (IQR) | 43.0 (25.0-74.0) | 38.0 (20.0-62.0) | 45.0 (26.0-74.0) | 50.0 (31.0-82.0) | 36.0 (21.0-71.0) | 43.0 (25.0-72.0) | 45.0 (25.0-78.0) |
| **Treatment** **variables** | | | | | | | |
| Extent of surgery |  |  |  |  |  |  |  |
| Complete mastectomy | 12,385 (32.40) | 2,478 (31.80) | 3,532 (32.39) | 4,120 (33.04) | 2,255 (31.93) | 6,829 (32.62) | 5,556 (32.13) |
| Mastectomy, NOS | 184 (0.48) | 75 (0.96) | 48 (0.44) | 38 (0.30) | 23 (0.33) | 129 (0.62) | 55 (0.32) |
| Modified radical mastectomy | 24,827 (64.95) | 5,051 (64.81) | 7,056 (64.71) | 8,073 (64.75) | 4,647 (65.80) | 13,468 (64.34) | 11,359 (65.69) |
| Radical mastectomy | 831 (2.17) | 189 (2.43) | 268 (2.46) | 237 (1.90) | 137 (1.94) | 508 (2.43) | 323 (1.87) |
| Use of radiation therapy |  |  |  |  |  |  |  |
| No | 6,283 (16.44) | 2,208 (28.33) | 1,879 (17.23) | 1,515 (12.15) | 681 (9.64) | 4,401 (21.02) | 1,882 (10.88) |
| Yes | 31,806 (83.20) | 5,499 (70.56) | 8,991 (82.46) | 10,940 (87.74) | 6,376 (90.29) | 16,411 (78.39) | 15,395 (89.02) |
| Missing | 138 (0.36) | 86 (1.10) | 34 (0.31) | 13 (0.10) | 5 (0.07) | 122 (0.58) | 16 (0.09) |
| Use of chemotherapy |  |  |  |  |  |  |  |
| No | 4,374 (11.44) | 1,073 (13.77) | 1,246 (11.43) | 1,328 (10.65) | 727 (10.29) | 2,552 (12.19) | 1,822 (10.54) |
| Yes | 33,528 (87.71) | 6,538 (83.90) | 9,580 (87.86) | 11,093 (88.97) | 6,317 (89.45) | 18,113 (86.52) | 15,415 (89.14) |
| Missing | 325 (0.85) | 182 (2.34) | 78 (0.72) | 47 (0.38) | 18 (0.25) | 269 (1.28) | 56 (0.32) |
| Use of hormone therapy |  |  |  |  |  |  |  |
| Yes | 28,044 (73.36) | 5,141 (65.97) | 8,012 (73.48) | 9,466 (75.92) | 5,425 (76.82) | 14,819 (70.79) | 13,225 (76.48) |
| No | 9,410 (24.62) | 2,309 (29.63) | 2,674 (24.52) | 2,846 (22.83) | 1,581 (22.39) | 5,520 (26.37) | 3,890 (22.49) |
| Missing | 773 (2.02) | 343 (4.40) | 218 (2.00) | 156 (1.25) | 56 (0.79) | 595 (2.84) | 178 (1.03) |

Abbreviations: EPR-expected performance rate, SD-standard deviation, mm-millimeter, IQR-interquartile range, ER-estrogen receptor, PR-progesterone receptor, NOS-not otherwise specified

Table S5. Patient data for all eligible 12RLN patients

| **Variable** | **Overall** | **Quartile 1** | **Quartile 2** | **Quartile 3** | **Quartile 4** | **Below EPR** | **Above EPR** |
| --- | --- | --- | --- | --- | --- | --- | --- |
| **Demographic variables** | | | | | | | |
| Age, mean (SD) | 70.0 (13.3) | 71.0 (13.0) | 70.0 (13.2) | 70.0 (13.3) | 69.0 (13.6) | 71.0 (13.1) | 70.0 (13.4) |
| Sex |  |  |  |  |  |  |  |
| Female | 94,736 (51.76) | 17,117 (51.42) | 23,350 (51.49) | 27,297 (52.11) | 26,972 (51.86) | 21,818 (51.34) | 72,918 (51.89) |
| Male | 88,290 (48.24) | 16,173 (48.58) | 21,995 (48.51) | 25,084 (47.89) | 25,038 (48.14) | 20,680 (48.66) | 67,610 (48.11) |
| Race |  |  |  |  |  |  |  |
| White | 152,552 (83.35) | 28,042 (84.24) | 37,248 (82.14) | 44,090 (84.17) | 43,172 (83.01) | 35,208 (82.85) | 117,344 (83.50) |
| Black | 21,843 (11.93) | 4,227 (12.70) | 5,884 (12.98) | 5,976 (11.41) | 5,756 (11.07) | 5,870 (13.81) | 15,973 (11.37) |
| Other | 7,354 (4.02) | 902 (2.71) | 1,808 (3.99) | 1,986 (3.79) | 2,658 (5.11) | 1,210 (2.85) | 6,144 (4.37) |
| Unknown | 1,277 (0.70) | 119 (0.36) | 405 (0.89) | 329 (0.63) | 424 (0.82) | 210 (0.49) | 1,067 (0.76) |
| Insurance Status |  |  |  |  |  |  |  |
| Private Insurance | 57,541 (31.44) | 9,590 (28.81) | 13,876 (30.60) | 16,648 (31.78) | 17,427 (33.51) | 12,205 (28.72) | 45,336 (32.26) |
| Medicaid | 8,248 (4.51) | 1,438 (4.32) | 2,117 (4.67) | 2,418 (4.62) | 2,275 (4.37) | 2,068 (4.87) | 6,180 (4.40) |
| Medicare | 107,259 (58.60) | 20,220 (60.74) | 26,707 (58.90) | 30,605 (58.43) | 29,727 (57.16) | 25,614 (60.27) | 81,645 (58.10) |
| Other Government | 1,523 (0.83) | 325 (0.98) | 373 (0.82) | 418 (0.80) | 407 (0.78) | 389 (0.92) | 1,134 (0.81) |
| Not Insured | 5,802 (3.17) | 1,151 (3.46) | 1,582 (3.49) | 1,733 (3.31) | 1,336 (2.57) | 1,517 (3.57) | 4,285 (3.05) |
| Unknown | 2,653 (1.45) | 566 (1.70) | 690 (1.52) | 559 (1.07) | 838 (1.61) | 705 (1.66) | 1,948 (1.39) |
| Education (% without high school degree) |  |  |  |  |  |  |  |
| <7% | 42,911 (23.45) | 5,818 (17.48) | 9,453 (20.85) | 13,335 (25.46) | 14,305 (27.50) | 7,361 (17.32) | 35,550 (25.30) |
| 7.0-12.9% | 59,940 (32.75) | 10,794 (32.42) | 14,662 (32.33) | 17,303 (33.03) | 17,181 (33.03) | 13,838 (32.56) | 46,102 (32.81) |
| 13-20.9% | 47,595 (26.00) | 9,901 (29.74) | 12,600 (27.79) | 12,884 (24.60) | 12,210 (23.48) | 12,461 (29.32) | 35,134 (25.00) |
| >=21% | 32,130 (17.55) | 6,702 (20.13) | 8,516 (18.78) | 8,706 (16.62) | 8,206 (15.78) | 8,742 (20.57) | 23,388 (16.64) |
| Missing | 544 (0.30) | 80 (0.24) | 148 (0.33) | 178 (0.34) | 138 (0.27) | 109 (0.26) | 435 (0.31) |
| Charlson Deyo comorbidity score |  |  |  |  |  |  |  |
| 0 | 122,233 (66.78) | 22,176 (66.61) | 30,473 (67.20) | 34,854 (66.54) | 34,730 (66.78) | 28,263 (66.50) | 93,970 (66.87) |
| 1 | 43,341 (23.68) | 7,895 (23.72) | 10,699 (23.59) | 12,422 (23.71) | 12,325 (23.70) | 10,154 (23.89) | 33,187 (23.62) |
| 2 | 12,263 (6.70) | 2,339 (7.03) | 2,948 (6.50) | 3,549 (6.78) | 3,427 (6.59) | 2,944 (6.93) | 9,319 (6.63) |
| >2 | 5,189 (2.84) | 880 (2.64) | 1,225 (2.70) | 1,556 (2.97) | 1,528 (2.94) | 1,137 (2.68) | 4,052 (2.88) |
| **Cancer variables** | | | | | | | |
| Tumor size (mm), median (IQR) | 42.0 (29.0-60.0) | 42.0 (30.0-60.0) | 42.0 (29.0-60.0) | 42.0 (29.0-60.0) | 42.0 (28.0-60.0) | 42.0 (30.0-60.0) | 42.0 (28.0-60.0) |
| Tumor Grade |  |  |  |  |  |  |  |
| Well differentiated | 18,927 (10.34) | 3,971 (11.93) | 4,862 (10.72) | 5,099 (9.73) | 4,995 (9.60) | 4,869 (11.46) | 14,058 (10.00) |
| Moderately differentiated | 123,256 (67.34) | 22,204 (66.70) | 30,565 (67.41) | 35,311 (67.41) | 35,176 (67.63) | 28,458 (66.96) | 94,798 (67.46) |
| Poorly differentiated | 28,860 (15.77) | 5,202 (15.63) | 6,959 (15.35) | 8,233 (15.72) | 8,466 (16.28) | 6,728 (15.83) | 22,132 (15.75) |
| Undifferentiated | 5,508 (3.01) | 796 (2.39) | 1,299 (2.86) | 1,845 (3.52) | 1,568 (3.01) | 956 (2.25) | 4,552 (3.24) |
| Unknown | 6,475 (3.54) | 1,117 (3.36) | 1,660 (3.66) | 1,893 (3.61) | 1,805 (3.47) | 1,487 (3.50) | 4,988 (3.55) |
| Lymph node status |  |  |  |  |  |  |  |
| Negative | 119,325 (65.20) | 21,585 (64.84) | 29,630 (65.34) | 34,158 (65.21) | 33,952 (65.28) | 27,570 (64.87) | 91,755 (65.29) |
| Positive | 62,628 (34.22) | 11,308 (33.97) | 15,446 (34.06) | 17,979 (34.32) | 17,895 (34.41) | 14,467 (34.04) | 48,161 (34.27) |
| Missing | 1,073 (0.59) | 397 (1.19) | 269 (0.59) | 244 (0.47) | 163 (0.31) | 461 (1.08) | 612 (0.44) |
| Overall stage |  |  |  |  |  |  |  |
| 1 | 48,976 (26.76) | 8,648 (25.98) | 12,247 (27.01) | 14,023 (26.77) | 14,058 (27.03) | 11,156 (26.25) | 37,820 (26.91) |
| 2 | 68,250 (37.29) | 12,714 (38.19) | 16,894 (37.26) | 19,445 (37.12) | 19,197 (36.91) | 16,092 (37.87) | 52,158 (37.12) |
| 3 | 65,800 (35.95) | 11,928 (35.83) | 16,204 (35.73) | 18,913 (36.11) | 18,755 (36.06) | 15,250 (35.88) | 50,550 (35.97) |
| **Hospital variables** | | | | | | | |
| Facility type |  |  |  |  |  |  |  |
| Academic/Research Program | 46,813 (25.58) | 2,962 (8.90) | 10,599 (23.37) | 16,009 (30.56) | 17,243 (33.15) | 4,751 (11.18) | 42,062 (29.93) |
| Community Cancer Program | 24,017 (13.12) | 9,640 (28.96) | 5,100 (11.25) | 4,872 (9.30) | 4,405 (8.47) | 11,132 (26.19) | 12,885 (9.17) |
| Comprehensive Community Cancer Program | 88,461 (48.33) | 18,033 (54.17) | 24,781 (54.65) | 23,195 (44.28) | 22,452 (43.17) | 22,929 (53.95) | 65,532 (46.63) |
| Integrated Network Cancer Program | 19,924 (10.89) | 2,094 (6.29) | 4,018 (8.86) | 7,195 (13.74) | 6,617 (12.72) | 2,948 (6.94) | 16,976 (12.08) |
| Missing | 3,811 (2.08) | 561 (1.69) | 847 (1.87) | 1,110 (2.12) | 1,293 (2.49) | 738 (1.74) | 3,073 (2.19) |
| Geographic location |  |  |  |  |  |  |  |
| East North Central | 32,936 (18.00) | 4,928 (14.80) | 7,812 (17.23) | 8,905 (17.00) | 11,291 (21.71) | 6,477 (15.24) | 26,459 (18.83) |
| East South Central | 13,298 (7.27) | 4,267 (12.82) | 3,912 (8.63) | 2,682 (5.12) | 2,437 (4.69) | 5,482 (12.90) | 7,816 (5.56) |
| Middle Atlantic | 27,245 (14.89) | 4,801 (14.42) | 5,858 (12.92) | 9,074 (17.32) | 7,512 (14.44) | 6,514 (15.33) | 20,731 (14.75) |
| Mountain | 7,237 (3.95) | 1,368 (4.11) | 2,407 (5.31) | 2,001 (3.82) | 1,461 (2.81) | 1,747 (4.11) | 5,490 (3.91) |
| New England | 9,655 (5.28) | 1,452 (4.36) | 1,695 (3.74) | 2,708 (5.17) | 3,800 (7.31) | 1,878 (4.42) | 7,777 (5.53) |
| Pacific | 19,659 (10.74) | 3,246 (9.75) | 5,050 (11.14) | 5,873 (11.21) | 5,490 (10.56) | 3,975 (9.35) | 15,684 (11.16) |
| South Atlantic | 38,835 (21.22) | 8,128 (24.42) | 11,618 (25.62) | 10,251 (19.57) | 8,838 (16.99) | 10,230 (24.07) | 28,605 (20.36) |
| West North Central | 14,197 (7.76) | 2,007 (6.03) | 2,523 (5.56) | 4,902 (9.36) | 4,765 (9.16) | 2,275 (5.35) | 11,922 (8.48) |
| West South Central | 16,153 (8.83) | 2,532 (7.61) | 3,623 (7.99) | 4,875 (9.31) | 5,123 (9.85) | 3,182 (7.49) | 12,971 (9.23) |
| Missing | 3,811 (2.08) | 561 (1.69) | 847 (1.87) | 1,110 (2.12) | 1,293 (2.49) | 738 (1.74) | 3,073 (2.19) |
| Number of reporting facilities |  |  |  |  |  |  |  |
| Single | 170,142 (92.96) | 31,572 (94.84) | 42,003 (92.63) | 48,552 (92.69) | 48,015 (92.32) | 40,241 (94.69) | 129,901 (92.44) |
| Multiple | 12,884 (7.04) | 1,718 (5.16) | 3,342 (7.37) | 3,829 (7.31) | 3,995 (7.68) | 2,257 (5.31) | 10,627 (7.56) |
| Travel distance in miles, median (IQR) | 7.7 (3.6-17.1) | 6.9 (3.1-15.2) | 7.4 (3.6-15.8) | 7.6 (3.6-16.6) | 8.7 (4.0-20.1) | 6.9 (3.2-15.1) | 8.0 (3.8-17.7) |
| Number of patients treated, median (IQR) | 174.0 (117.0-279.0) | 122.0 (86.0-199.0) | 171.0 (122.0-249.0) | 197.0 (128.0-294.0) | 213.0 (138.0-333.0) | 127.0 (87.0-199.0) | 196.0 (131.0-297.0) |
| **Treatment variables** | | | | | | | |
| Extent of surgery |  |  |  |  |  |  |  |
| Segmental colectomy | 61,571 (33.64) | 11,587 (34.81) | 15,662 (34.54) | 17,466 (33.34) | 16,856 (32.41) | 14,697 (34.58) | 46,874 (33.36) |
| Subtotal colectomy | 113,889 (62.23) | 20,642 (62.01) | 28,032 (61.82) | 32,940 (62.89) | 32,275 (62.06) | 26,393 (62.10) | 87,496 (62.26) |
| Total colectomy | 4,669 (2.55) | 694 (2.08) | 1,020 (2.25) | 1,287 (2.46) | 1,668 (3.21) | 926 (2.18) | 3,743 (2.66) |
| Total proctocolectomy | 1,041 (0.57) | 78 (0.23) | 220 (0.49) | 292 (0.56) | 451 (0.87) | 114 (0.27) | 927 (0.66) |
| Surgery, NOS | 1,835 (1.00) | 286 (0.86) | 402 (0.89) | 391 (0.75) | 756 (1.45) | 364 (0.86) | 1,471 (1.05) |
| Missing | 21 (0.01) | 3 (0.01) | 9 (0.02) | 5 (0.01) | 4 (0.01) | 4 (0.01) | 17 (0.01) |
| Use of radiation therapy |  |  |  |  |  |  |  |
| No | 179,371 (98.00) | 32,575 (97.85) | 44,340 (97.78) | 51,385 (98.10) | 51,071 (98.19) | 41,534 (97.73) | 137,837 (98.09) |
| Yes | 2,248 (1.23) | 432 (1.30) | 573 (1.26) | 583 (1.11) | 660 (1.27) | 541 (1.27) | 1,707 (1.21) |
| Missing | 1,407 (0.77) | 283 (0.85) | 432 (0.95) | 413 (0.79) | 279 (0.54) | 423 (1.00) | 984 (0.70) |
| Use of chemotherapy |  |  |  |  |  |  |  |
| No | 121,468 (66.37) | 22,120 (66.45) | 30,193 (66.59) | 34,831 (66.50) | 34,324 (66.00) | 28,105 (66.13) | 93,363 (66.44) |
| Yes | 54,635 (29.85) | 9,827 (29.52) | 13,372 (29.49) | 15,503 (29.60) | 15,933 (30.63) | 12,613 (29.68) | 42,022 (29.90) |
| Missing | 6,923 (3.78) | 1,343 (4.03) | 1,780 (3.93) | 2,047 (3.91) | 1,753 (3.37) | 1,780 (4.19) | 5,143 (3.66) |
| Number of lymph nodes examined, median (IQR) | 18.0 (14.0-24.0) | 15.0 (12.0-20.0) | 17.0 (13.0-23.0) | 18.0 (14.0-25.0) | 20.0 (16.0-28.0) | 15.0 (12.0-20.0) | 19.0 (14.0-25.0) |

Abbreviations: EPR-expected performance rate, SD-standard deviation, mm-millimeter, IQR-interquartile range, NOS-not otherwise specified

Table S6. Patient data for all eligible LNoSurg patients

| **Variable** | **Overall** | **Quartile 1** | **Quartile 2** | **Quartile 3** | **Quartile 4** | **Below EPR** | **Above EPR** |
| --- | --- | --- | --- | --- | --- | --- | --- |
| **Demographic variables** | | | | | | | |
| Age, mean (SD) | 67.0 (10.6) | 67.0 (10.5) | 67.0 (10.7) | 68.0 (10.5) | 68.0 (10.7) | 68.0 (10.4) | 67.0 (10.6) |
| Sex |  |  |  |  |  |  |  |
| Female | 20,707 (44.68) | 5,321 (44.73) | 6,910 (44.78) | 6,372 (44.46) | 2,104 (44.83) | 3,128 (44.83) | 17,579 (44.65) |
| Male | 38,345 (55.71) | 10,050 (55.15) | 11,159 (55.86) | 11,488 (55.73) | 5,648 (56.34) | 7,214 (55.23) | 31,131 (55.82) |
| Race |  |  |  |  |  |  |  |
| White | 38,504 (83.07) | 9,926 (83.44) | 12,700 (82.31) | 11,946 (83.36) | 3,932 (83.78) | 5,855 (83.92) | 32,649 (82.92) |
| Black | 6,184 (13.34) | 1,487 (12.50) | 2,128 (13.79) | 1,951 (13.61) | 618 (13.17) | 782 (11.21) | 5,402 (13.72) |
| Other | 1,360 (2.93) | 389 (3.27) | 482 (3.12) | 367 (2.56) | 122 (2.60) | 281 (4.03) | 1,079 (2.74) |
| Unknown | 302 (0.65) | 94 (0.79) | 120 (0.78) | 67 (0.47) | 21 (0.45) | 59 (0.85) | 243 (0.62) |
| Insurance Status |  |  |  |  |  |  |  |
| Private Insurance | 12,517 (27.01) | 3,351 (28.17) | 4,233 (27.43) | 3,807 (26.56) | 1,126 (23.99) | 2,016 (28.89) | 10,501 (26.67) |
| Medicaid | 3,723 (8.03) | 894 (7.52) | 1,302 (8.44) | 1,128 (7.87) | 399 (8.50) | 485 (6.95) | 3,238 (8.22) |
| Medicare | 26,425 (57.01) | 6,838 (57.48) | 8,789 (56.96) | 8,004 (55.85) | 2,794 (59.54) | 4,033 (57.80) | 22,392 (56.87) |
| Other Government | 976 (2.11) | 251 (2.11) | 299 (1.94) | 368 (2.57) | 58 (1.24) | 128 (1.83) | 848 (2.15) |
| Not Insured | 1,845 (3.98) | 381 (3.20) | 584 (3.78) | 693 (4.84) | 187 (3.98) | 215 (3.08) | 1,630 (4.14) |
| Unknown | 864 (1.86) | 181 (1.52) | 223 (1.45) | 331 (2.31) | 129 (2.75) | 100 (1.43) | 764 (1.94) |
| Education (% without high school degree) |  |  |  |  |  |  |  |
| <7% | 8,198 (17.69) | 2,291 (19.26) | 2,753 (17.84) | 2,368 (16.52) | 786 (16.75) | 1,338 (19.18) | 6,860 (17.42) |
| 7.0-12.9% | 14,784 (31.90) | 3,845 (32.32) | 4,910 (31.82) | 4,534 (31.64) | 1,495 (31.86) | 2,169 (31.09) | 12,615 (32.04) |
| 13-20.9% | 13,940 (30.08) | 3,292 (27.67) | 4,723 (30.61) | 4,423 (30.86) | 1,502 (32.01) | 1,963 (28.14) | 11,977 (30.42) |
| >=21% | 9,148 (19.74) | 2,377 (19.98) | 2,969 (19.24) | 2,923 (20.40) | 879 (18.73) | 1,440 (20.64) | 7,708 (19.58) |
| Missing | 280 (0.60) | 91 (0.76) | 75 (0.49) | 83 (0.58) | 31 (0.66) | 67 (0.96) | 213 (0.54) |
| Charlson Deyo comorbidity score |  |  |  |  |  |  |  |
| 0 | 26,458 (57.08) | 6,876 (57.80) | 8,744 (56.67) | 8,216 (57.33) | 2,622 (55.87) | 4,056 (58.13) | 22,402 (56.90) |
| 1 | 13,519 (29.17) | 3,414 (28.70) | 4,557 (29.53) | 4,165 (29.06) | 1,383 (29.47) | 2,015 (28.88) | 11,504 (29.22) |
| 2 | 4,653 (10.04) | 1,165 (9.79) | 1,543 (10.00) | 1,429 (9.97) | 516 (11.00) | 668 (9.57) | 3,985 (10.12) |
| >2 | 1,720 (3.71) | 441 (3.71) | 586 (3.80) | 521 (3.64) | 172 (3.67) | 238 (3.41) | 1,482 (3.76) |
| **Cancer variables** | | | | | | | |
| Tumor size (mm), median (IQR) | 45.0 (30.0-65.0) | 44.0 (28.0-63.0) | 45.0 (30.0-65.0) | 46.0 (30.0-65.5) | 48.0 (31.0-69.0) | 43.0 (28.0-63.0) | 46.0 (30.0-65.0) |
| Histology |  |  |  |  |  |  |  |
| Adenocarcinoma | 18,008 (38.85) | 4,902 (41.21) | 5,989 (38.81) | 5,420 (37.82) | 1,697 (36.16) | 2,892 (41.45) | 15,116 (38.39) |
| Squamous | 20,501 (44.23) | 4,984 (41.90) | 6,758 (43.80) | 6,491 (45.29) | 2,268 (48.33) | 2,868 (41.11) | 17,633 (44.78) |
| Adenosquamous | 686 (1.48) | 221 (1.86) | 198 (1.28) | 214 (1.49) | 53 (1.13) | 135 (1.93) | 551 (1.40) |
| Bronchioloalveolar | 763 (1.65) | 232 (1.95) | 351 (2.27) | 143 (1.00) | 37 (0.79) | 158 (2.26) | 605 (1.54) |
| Large cell | 616 (1.33) | 147 (1.24) | 218 (1.41) | 182 (1.27) | 69 (1.47) | 90 (1.29) | 526 (1.34) |
| Other | 5,776 (12.46) | 1,410 (11.85) | 1,916 (12.42) | 1,881 (13.13) | 569 (12.12) | 834 (11.95) | 4,942 (12.55) |
| Tumor stage |  |  |  |  |  |  |  |
| 0 | 191 (0.41) | 50 (0.42) | 57 (0.37) | 63 (0.44) | 21 (0.45) | 28 (0.40) | 163 (0.41) |
| 1 | 7,409 (15.98) | 2,152 (18.09) | 2,474 (16.03) | 2,166 (15.11) | 617 (13.15) | 1,318 (18.89) | 6,091 (15.47) |
| 2 | 15,769 (34.02) | 4,050 (34.05) | 5,352 (34.69) | 4,833 (33.72) | 1,534 (32.69) | 2,383 (34.16) | 13,386 (34.00) |
| 3 | 10,464 (22.58) | 2,552 (21.45) | 3,391 (21.98) | 3,408 (23.78) | 1,113 (23.72) | 1,492 (21.38) | 8,972 (22.79) |
| 4 | 10,397 (22.43) | 2,533 (21.29) | 3,472 (22.50) | 3,232 (22.55) | 1,160 (24.72) | 1,412 (20.24) | 8,985 (22.82) |
| Missing | 2,120 (4.57) | 559 (4.70) | 684 (4.43) | 629 (4.39) | 248 (5.28) | 344 (4.93) | 1,776 (4.51) |
| Tumor Grade |  |  |  |  |  |  |  |
| Well differentiated | 1,164 (2.51) | 322 (2.71) | 389 (2.52) | 344 (2.40) | 109 (2.32) | 191 (2.74) | 973 (2.47) |
| Moderately differentiated | 8,414 (18.15) | 2,414 (20.29) | 2,659 (17.23) | 2,533 (17.67) | 808 (17.22) | 1,437 (20.60) | 6,977 (17.72) |
| Poorly differentiated | 15,563 (33.58) | 4,156 (34.94) | 5,196 (33.67) | 4,634 (32.34) | 1,577 (33.60) | 2,465 (35.33) | 13,098 (33.27) |
| Undifferentiated | 484 (1.04) | 137 (1.15) | 141 (0.91) | 148 (1.03) | 58 (1.24) | 87 (1.25) | 397 (1.01) |
| Unknown | 20,725 (44.71) | 4,867 (40.91) | 7,045 (45.66) | 6,672 (46.56) | 2,141 (45.62) | 2,797 (40.09) | 17,928 (45.53) |
| Lymph node status |  |  |  |  |  |  |  |
| Negative | 2,963 (6.39) | 1,015 (8.53) | 1,030 (6.68) | 744 (5.19) | 174 (3.71) | 616 (8.83) | 2,347 (5.96) |
| Positive | 15,015 (32.39) | 4,570 (38.42) | 5,258 (34.08) | 4,213 (29.40) | 974 (20.75) | 2,799 (40.12) | 12,216 (31.03) |
| Missing | 28,372 (61.21) | 6,311 (53.05) | 9,142 (59.25) | 9,374 (65.41) | 3,545 (75.54) | 3,562 (51.05) | 24,810 (63.01) |
| pN stage |  |  |  |  |  |  |  |
| 0 | 2,698 (5.82) | 968 (8.14) | 992 (6.43) | 605 (4.22) | 133 (2.83) | 586 (8.40) | 2,112 (5.36) |
| 1 | 911 (1.97) | 307 (2.58) | 355 (2.30) | 211 (1.47) | 38 (0.81) | 194 (2.78) | 717 (1.82) |
| 2 | 6,078 (13.11) | 2,356 (19.80) | 2,099 (13.60) | 1,335 (9.32) | 288 (6.14) | 1,541 (22.09) | 4,537 (11.52) |
| 3 | 61 (0.13) | 25 (0.21) | 20 (0.13) | 14 (0.10) | 2 (0.04) | 17 (0.24) | 44 (0.11) |
| X | 16,940 (36.55) | 4,065 (34.17) | 5,819 (37.71) | 5,293 (36.93) | 1,763 (37.57) | 2,305 (33.04) | 14,635 (37.17) |
| Missing | 19,662 (42.42) | 4,175 (35.10) | 6,145 (39.83) | 6,873 (47.96) | 2,469 (52.61) | 2,334 (33.45) | 17,328 (44.01) |
| Tumor Location |  |  |  |  |  |  |  |
| Bilateral involvement | 28 (0.06) | 12 (0.10) | 6 (0.04) | 6 (0.04) | 4 (0.09) | 11 (0.16) | 17 (0.04) |
| Left Lower Lobe | 4,041 (8.72) | 1,026 (8.62) | 1,369 (8.87) | 1,243 (8.67) | 403 (8.59) | 603 (8.64) | 3,438 (8.73) |
| Left Lung, NOS | 969 (2.09) | 267 (2.24) | 311 (2.02) | 288 (2.01) | 103 (2.19) | 176 (2.52) | 793 (2.01) |
| Left Overlapping Lesion of Lung | 187 (0.40) | 58 (0.49) | 62 (0.40) | 38 (0.27) | 29 (0.62) | 40 (0.57) | 147 (0.37) |
| Left Upper Lobe | 10,523 (22.70) | 2,779 (23.36) | 3,518 (22.80) | 3,153 (22.00) | 1,073 (22.86) | 1,590 (22.79) | 8,933 (22.69) |
| Main Bronchus | 2,708 (5.84) | 649 (5.46) | 933 (6.05) | 809 (5.65) | 317 (6.75) | 379 (5.43) | 2,329 (5.92) |
| Only one side involved, right or left or | 61 (0.13) | 24 (0.20) | 18 (0.12) | 14 (0.10) | 5 (0.11) | 12 (0.17) | 49 (0.12) |
| Paired site midline tumor | 15 (0.03) | 5 (0.04) | 5 (0.03) | 4 (0.03) | 1 (0.02) | 5 (0.07) | 10 (0.03) |
| Right Lower Lobe | 6,853 (14.79) | 1,775 (14.92) | 2,250 (14.58) | 2,123 (14.81) | 705 (15.02) | 1,056 (15.14) | 5,797 (14.72) |
| Right Lung, NOS | 1,966 (4.24) | 507 (4.26) | 657 (4.26) | 620 (4.33) | 182 (3.88) | 301 (4.31) | 1,665 (4.23) |
| Right Middle Lobe | 1,804 (3.89) | 452 (3.80) | 592 (3.84) | 562 (3.92) | 198 (4.22) | 267 (3.83) | 1,537 (3.90) |
| Right Overlapping Lesion of Lung | 544 (1.17) | 157 (1.32) | 189 (1.22) | 146 (1.02) | 52 (1.11) | 102 (1.46) | 442 (1.12) |
| Right Upper Lobe | 16,000 (34.52) | 4,026 (33.84) | 5,295 (34.32) | 5,112 (35.67) | 1,567 (33.39) | 2,333 (33.44) | 13,667 (34.71) |
| Missing | 651 (1.40) | 159 (1.34) | 225 (1.46) | 213 (1.49) | 54 (1.15) | 102 (1.46) | 549 (1.39) |
| Overall stage |  |  |  |  |  |  |  |
| 0 | 127 (0.27) | 31 (0.26) | 42 (0.27) | 36 (0.25) | 18 (0.38) | 15 (0.21) | 112 (0.28) |
| 1 | 1,387 (2.99) | 515 (4.33) | 512 (3.32) | 290 (2.02) | 70 (1.49) | 316 (4.53) | 1,071 (2.72) |
| 2 | 1,360 (2.93) | 482 (4.05) | 491 (3.18) | 318 (2.22) | 69 (1.47) | 303 (4.34) | 1,057 (2.68) |
| 3 | 42,363 (91.40) | 10,547 (88.66) | 14,055 (91.09) | 13,368 (93.28) | 4,393 (93.61) | 6,129 (87.85) | 36,234 (92.03) |
| 4 | 418 (0.90) | 151 (1.27) | 98 (0.64) | 117 (0.82) | 52 (1.11) | 120 (1.72) | 298 (0.76) |
| Occult | 20 (0.04) | 6 (0.05) | 7 (0.05) | 5 (0.03) | 2 (0.04) | 3 (0.04) | 17 (0.04) |
| Missing | 675 (1.46) | 164 (1.38) | 225 (1.46) | 197 (1.37) | 89 (1.90) | 91 (1.30) | 584 (1.48) |
| **Hospital variables** | | | | | | | |
| Facility type |  |  |  |  |  |  |  |
| Academic/Research Program | 14,700 (31.72) | 4,295 (36.10) | 6,251 (40.51) | 3,779 (26.37) | 375 (7.99) | 2,521 (36.13) | 12,179 (30.93) |
| Community Cancer Program | 5,476 (11.81) | 1,300 (10.93) | 1,043 (6.76) | 1,338 (9.34) | 1,795 (38.25) | 949 (13.60) | 4,527 (11.50) |
| Comprehensive Community Cancer Program | 21,270 (45.89) | 5,026 (42.25) | 6,337 (41.07) | 8,084 (56.41) | 1,823 (38.85) | 2,882 (41.31) | 18,388 (46.70) |
| Integrated Network Cancer Program | 4,713 (10.17) | 1,224 (10.29) | 1,725 (11.18) | 1,087 (7.58) | 677 (14.43) | 596 (8.54) | 4,117 (10.46) |
| Missing | 191 (0.41) | 51 (0.43) | 74 (0.48) | 43 (0.30) | 23 (0.49) | 29 (0.42) | 162 (0.41) |
| Geographic location |  |  |  |  |  |  |  |
| East North Central | 8,974 (19.36) | 2,340 (19.67) | 2,939 (19.05) | 2,759 (19.25) | 936 (19.94) | 1,024 (14.68) | 7,950 (20.19) |
| East South Central | 4,204 (9.07) | 1,526 (12.83) | 1,307 (8.47) | 1,205 (8.41) | 166 (3.54) | 972 (13.93) | 3,232 (8.21) |
| Middle Atlantic | 6,925 (14.94) | 1,887 (15.86) | 2,731 (17.70) | 1,842 (12.85) | 465 (9.91) | 1,068 (15.31) | 5,857 (14.88) |
| Mountain | 1,367 (2.95) | 220 (1.85) | 422 (2.73) | 519 (3.62) | 206 (4.39) | 150 (2.15) | 1,217 (3.09) |
| New England | 2,709 (5.84) | 561 (4.72) | 1,040 (6.74) | 763 (5.32) | 345 (7.35) | 309 (4.43) | 2,400 (6.10) |
| Pacific | 4,228 (9.12) | 1,351 (11.36) | 1,178 (7.63) | 1,120 (7.82) | 579 (12.34) | 964 (13.82) | 3,264 (8.29) |
| South Atlantic | 10,785 (23.27) | 2,820 (23.71) | 3,240 (21.00) | 3,491 (24.36) | 1,234 (26.29) | 1,702 (24.39) | 9,083 (23.07) |
| West North Central | 3,482 (7.51) | 520 (4.37) | 1,499 (9.71) | 1,090 (7.61) | 373 (7.95) | 316 (4.53) | 3,166 (8.04) |
| West South Central | 3,485 (7.52) | 620 (5.21) | 1,000 (6.48) | 1,499 (10.46) | 366 (7.80) | 443 (6.35) | 3,042 (7.73) |
| Missing | 191 (0.41) | 51 (0.43) | 74 (0.48) | 43 (0.30) | 23 (0.49) | 29 (0.42) | 162 (0.41) |
| Number of reporting facilities |  |  |  |  |  |  |  |
| Single | 38,407 (82.86) | 9,781 (82.22) | 12,875 (83.44) | 11,991 (83.67) | 3,760 (80.12) | 5,739 (82.26) | 32,668 (82.97) |
| Multiple | 7,943 (17.14) | 2,115 (17.78) | 2,555 (16.56) | 2,340 (16.33) | 933 (19.88) | 1,238 (17.74) | 6,705 (17.03) |
| Travel distance in miles, median (IQR) | 9.2 (4.0-21.8) | 9.1 (4.1-21.9) | 9.7 (4.2-23.6) | 9.4 (4.1-22.2) | 7.2 (2.9-16.4) | 9.1 (4.0-22.1) | 9.2 (4.1-21.8) |
| Number of patients treated, median (IQR) | 52.0 (31.0-81.0) | 51.0 (32.0-81.0) | 65.0 (40.0-108.0) | 54.0 (36.0-77.0) | 21.0 (13.0-32.0) | 45.0 (27.0-73.0) | 53.0 (32.0-82.0) |
| **Treatment variables** | | | | | | | |
| Extent of Surgery |  |  |  |  |  |  |  |
| Sublobar Resection | 796 (1.72) | 345 (2.90) | 297 (1.92) | 147 (1.03) | 7 (0.15) | 240 (3.44) | 556 (1.41) |
| Lobectomy | 5,073 (10.94) | 2,072 (17.42) | 1,794 (11.63) | 1,012 (7.06) | 195 (4.16) | 1,365 (19.56) | 3,708 (9.42) |
| Pneumonectomy | 856 (1.85) | 344 (2.89) | 322 (2.09) | 163 (1.14) | 27 (0.58) | 241 (3.45) | 615 (1.56) |
| Other | 777 (1.68) | 320 (2.69) | 286 (1.85) | 154 (1.07) | 17 (0.36) | 194 (2.78) | 583 (1.48) |
| No Surgery | 38,521 (83.11) | 8,722 (73.32) | 12,596 (81.63) | 12,776 (89.15) | 4,427 (94.33) | 4,903 (70.27) | 33,618 (85.38) |
| Missing | 327 (0.71) | 93 (0.78) | 135 (0.87) | 79 (0.55) | 20 (0.43) | 34 (0.49) | 293 (0.74) |
| Use of radiation therapy |  |  |  |  |  |  |  |
| No | 14,288 (30.83) | 4,365 (36.69) | 4,701 (30.47) | 3,948 (27.55) | 1,274 (27.15) | 2,773 (39.74) | 11,515 (29.25) |
| Yes | 31,844 (68.70) | 7,454 (62.66) | 10,648 (69.01) | 10,339 (72.14) | 3,403 (72.51) | 4,150 (59.48) | 27,694 (70.34) |
| Missing | 218 (0.47) | 77 (0.65) | 81 (0.52) | 44 (0.31) | 16 (0.34) | 54 (0.77) | 164 (0.42) |
| Use of chemotherapy |  |  |  |  |  |  |  |
| No | 12,750 (27.51) | 3,550 (29.84) | 4,116 (26.68) | 3,815 (26.62) | 1,269 (27.04) | 2,187 (31.35) | 10,563 (26.83) |
| Yes | 32,776 (70.71) | 8,093 (68.03) | 11,044 (71.57) | 10,280 (71.73) | 3,359 (71.57) | 4,629 (66.35) | 28,147 (71.49) |
| Missing | 824 (1.78) | 253 (2.13) | 270 (1.75) | 236 (1.65) | 65 (1.39) | 161 (2.31) | 663 (1.68) |
| Use of hormone therapy |  |  |  |  |  |  |  |
| No | 45,304 (97.74) | 11,588 (97.41) | 15,083 (97.75) | 14,033 (97.92) | 4,600 (98.02) | 6,799 (97.45) | 38,505 (97.80) |
| Yes | 178 (0.38) | 49 (0.41) | 52 (0.34) | 62 (0.43) | 15 (0.32) | 34 (0.49) | 144 (0.37) |
| Missing | 868 (1.87) | 259 (2.18) | 295 (1.91) | 236 (1.65) | 78 (1.66) | 144 (2.06) | 724 (1.84) |
| Use of immunotherapy |  |  |  |  |  |  |  |
| No | 46,064 (99.38) | 11,833 (99.47) | 15,311 (99.23) | 14,243 (99.39) | 4,677 (99.66) | 6,938 (99.44) | 39,126 (99.37) |
| Yes | 197 (0.43) | 42 (0.35) | 77 (0.50) | 62 (0.43) | 16 (0.34) | 23 (0.33) | 174 (0.44) |
| Missing | 89 (0.19) | 21 (0.18) | 42 (0.27) | 26 (0.18) |  | 16 (0.23) | 73 (0.19) |

Abbreviations: EPR-expected performance rate, SD-standard deviation, mm-millimeter, IQR-interquartile range, NOS-not otherwise specified

Table S7. Patient data for all eligible LCT patients

| **Variable** | **Overall** | **Quartile 1** | **Quartile 2** | **Quartile 3** | **Quartile 4** | **Below EPR** | **Above EPR** |
| --- | --- | --- | --- | --- | --- | --- | --- |
| **Demographic variables** | | | | | | | |
| Age, mean (SD) | 66.0 (9.9) | 67.0 (10.0) | 66.0 (9.9) | 65.0 (9.8) | 65.0 (9.8) | 66.0 (10.0) | 66.0 (9.8) |
| Sex |  |  |  |  |  |  |  |
| Female | 10,226 (48.81) | 2,128 (49.88) | 2,221 (48.64) | 3,815 (48.69) | 2,062 (48.12) | 2,924 (49.37) | 7,302 (48.59) |
| Male | 10,726 (51.19) | 2,138 (50.12) | 2,345 (51.36) | 4,020 (51.31) | 2,223 (51.88) | 2,999 (50.63) | 7,727 (51.41) |
| Race |  |  |  |  |  |  |  |
| White | 18,027 (86.04) | 3,536 (82.89) | 3,977 (87.10) | 6,820 (87.05) | 3,694 (86.21) | 4,981 (84.10) | 13,046 (86.81) |
| Black | 1,982 (9.46) | 405 (9.49) | 404 (8.85) | 725 (9.25) | 448 (10.46) | 561 (9.47) | 1,421 (9.46) |
| Other | 806 (3.85) | 278 (6.52) | 152 (3.33) | 247 (3.15) | 129 (3.01) | 317 (5.35) | 489 (3.25) |
| Unknown | 137 (0.65) | 47 (1.10) | 33 (0.72) | 43 (0.55) | 14 (0.33) | 64 (1.08) | 73 (0.49) |
| Insurance Status |  |  |  |  |  |  |  |
| Private Insurance | 7,527 (35.92) | 1,455 (34.11) | 1,602 (35.09) | 2,927 (37.36) | 1,543 (36.01) | 2,064 (34.85) | 5,463 (36.35) |
| Medicaid | 1,288 (6.15) | 284 (6.66) | 283 (6.20) | 438 (5.59) | 283 (6.60) | 380 (6.42) | 908 (6.04) |
| Medicare | 10,990 (52.45) | 2,243 (52.58) | 2,450 (53.66) | 4,091 (52.21) | 2,206 (51.48) | 3,103 (52.39) | 7,887 (52.48) |
| Other Government | 288 (1.37) | 49 (1.15) | 66 (1.45) | 121 (1.54) | 52 (1.21) | 80 (1.35) | 208 (1.38) |
| Not Insured | 543 (2.59) | 101 (2.37) | 117 (2.56) | 175 (2.23) | 150 (3.50) | 143 (2.41) | 400 (2.66) |
| Unknown | 316 (1.51) | 134 (3.14) | 48 (1.05) | 83 (1.06) | 51 (1.19) | 153 (2.58) | 163 (1.08) |
| Education (% without high school degree) |  |  |  |  |  |  |  |
| <7% | 4,626 (22.08) | 900 (21.10) | 1,083 (23.72) | 1,765 (22.53) | 878 (20.49) | 1,291 (21.80) | 3,335 (22.19) |
| 7.0-12.9% | 7,068 (33.73) | 1,265 (29.65) | 1,591 (34.84) | 2,644 (33.75) | 1,568 (36.59) | 1,820 (30.73) | 5,248 (34.92) |
| 13-20.9% | 5,710 (27.25) | 1,172 (27.47) | 1,165 (25.51) | 2,198 (28.05) | 1,175 (27.42) | 1,607 (27.13) | 4,103 (27.30) |
| >=21% | 3,475 (16.59) | 916 (21.47) | 711 (15.57) | 1,200 (15.32) | 648 (15.12) | 1,185 (20.01) | 2,290 (15.24) |
| Missing | 73 (0.35) | 13 (0.30) | 16 (0.35) | 28 (0.36) | 16 (0.37) | 20 (0.34) | 53 (0.35) |
| Charlson Deyo comorbidity score |  |  |  |  |  |  |  |
| 0 | 11,353 (54.19) | 2,429 (56.94) | 2,507 (54.91) | 4,141 (52.85) | 2,276 (53.12) | 3,339 (56.37) | 8,014 (53.32) |
| 1 | 6,972 (33.28) | 1,332 (31.22) | 1,509 (33.05) | 2,698 (34.44) | 1,433 (33.44) | 1,889 (31.89) | 5,083 (33.82) |
| 2 | 2,074 (9.90) | 404 (9.47) | 425 (9.31) | 786 (10.03) | 459 (10.71) | 555 (9.37) | 1,519 (10.11) |
| >2 | 553 (2.64) | 101 (2.37) | 125 (2.74) | 210 (2.68) | 117 (2.73) | 140 (2.36) | 413 (2.75) |
| **Cancer variables** | | | | | | | |
| Tumor size (mm), median (IQR) | 35.0 (23.0-50.0) | 35.0 (23.0-50.0) | 34.0 (24.0-50.0) | 35.0 (24.0-50.0) | 35.0 (23.0-50.0) | 35.0 (23.0-50.0) | 35.0 (23.0-50.0) |
| Histology |  |  |  |  |  |  |  |
| Adenocarcinoma | 10,947 (52.25) | 2,254 (52.84) | 2,420 (53.00) | 3,966 (50.62) | 2,307 (53.84) | 3,124 (52.74) | 7,823 (52.05) |
| Squamous | 6,364 (30.37) | 1,216 (28.50) | 1,372 (30.05) | 2,464 (31.45) | 1,312 (30.62) | 1,720 (29.04) | 4,644 (30.90) |
| Adenosquamous | 639 (3.05) | 130 (3.05) | 152 (3.33) | 214 (2.73) | 143 (3.34) | 197 (3.33) | 442 (2.94) |
| Bronchioloalveolar | 1,798 (8.58) | 403 (9.45) | 341 (7.47) | 769 (9.81) | 285 (6.65) | 525 (8.86) | 1,273 (8.47) |
| Large cell | 230 (1.10) | 48 (1.13) | 62 (1.36) | 80 (1.02) | 40 (0.93) | 71 (1.20) | 159 (1.06) |
| Other | 974 (4.65) | 215 (5.04) | 219 (4.80) | 342 (4.37) | 198 (4.62) | 286 (4.83) | 688 (4.58) |
| Tumor stage |  |  |  |  |  |  |  |
| 0 | 84 (0.40) | 13 (0.30) | 19 (0.42) | 37 (0.47) | 15 (0.35) | 19 (0.32) | 65 (0.43) |
| 1 | 5,997 (28.62) | 1,232 (28.88) | 1,320 (28.91) | 2,231 (28.47) | 1,214 (28.33) | 1,695 (28.62) | 4,302 (28.62) |
| 2 | 9,993 (47.69) | 2,067 (48.45) | 2,173 (47.59) | 3,697 (47.19) | 2,056 (47.98) | 2,871 (48.47) | 7,122 (47.39) |
| 3 | 3,739 (17.85) | 730 (17.11) | 800 (17.52) | 1,444 (18.43) | 765 (17.85) | 1,036 (17.49) | 2,703 (17.99) |
| 4 | 989 (4.72) | 191 (4.48) | 221 (4.84) | 377 (4.81) | 200 (4.67) | 257 (4.34) | 732 (4.87) |
| Missing | 150 (0.72) | 33 (0.77) | 33 (0.72) | 49 (0.63) | 35 (0.82) | 45 (0.76) | 105 (0.70) |
| Tumor Grade |  |  |  |  |  |  |  |
| Well differentiated | 1,037 (4.95) | 216 (5.06) | 237 (5.19) | 379 (4.84) | 205 (4.78) | 296 (5.00) | 741 (4.93) |
| Moderately differentiated | 8,901 (42.48) | 1,805 (42.31) | 2,007 (43.96) | 3,278 (41.84) | 1,811 (42.26) | 2,513 (42.43) | 6,388 (42.50) |
| Poorly differentiated | 9,328 (44.52) | 1,899 (44.51) | 2,006 (43.93) | 3,550 (45.31) | 1,873 (43.71) | 2,647 (44.69) | 6,681 (44.45) |
| Undifferentiated | 275 (1.31) | 63 (1.48) | 56 (1.23) | 103 (1.31) | 53 (1.24) | 83 (1.40) | 192 (1.28) |
| Unknown | 1,411 (6.73) | 283 (6.63) | 260 (5.69) | 525 (6.70) | 343 (8.00) | 384 (6.48) | 1,027 (6.83) |
| Lymph node status |  |  |  |  |  |  |  |
| Negative | 126 (0.60) | 36 (0.84) | 21 (0.46) | 46 (0.59) | 23 (0.54) | 44 (0.74) | 82 (0.55) |
| Positive | 20,674 (98.67) | 4,193 (98.29) | 4,513 (98.84) | 7,748 (98.89) | 4,220 (98.48) | 5,826 (98.36) | 14,848 (98.80) |
| Missing | 152 (0.73) | 37 (0.87) | 32 (0.70) | 41 (0.52) | 42 (0.98) | 53 (0.89) | 99 (0.66) |
| pN stage |  |  |  |  |  |  |  |
| 1 | 12,575 (60.02) | 2,555 (59.89) | 2,816 (61.67) | 4,673 (59.64) | 2,531 (59.07) | 3,612 (60.98) | 8,963 (59.64) |
| 2 | 8,377 (39.98) | 1,711 (40.11) | 1,750 (38.33) | 3,162 (40.36) | 1,754 (40.93) | 2,311 (39.02) | 6,066 (40.36) |
| Tumor Location |  |  |  |  |  |  |  |
| Bilateral involvement | 1 (0.00) |  |  | 1 (0.01) |  |  | 1 (0.01) |
| Left Lower Lobe | 3,155 (15.06) | 644 (15.10) | 712 (15.59) | 1,145 (14.61) | 654 (15.26) | 901 (15.21) | 2,254 (15.00) |
| Left Lung, NOS | 204 (0.97) | 44 (1.03) | 46 (1.01) | 77 (0.98) | 37 (0.86) | 61 (1.03) | 143 (0.95) |
| Left Overlapping Lesion of Lung | 185 (0.88) | 33 (0.77) | 36 (0.79) | 79 (1.01) | 37 (0.86) | 49 (0.83) | 136 (0.90) |
| Left Upper Lobe | 5,663 (27.03) | 1,152 (27.00) | 1,226 (26.85) | 2,124 (27.11) | 1,161 (27.09) | 1,613 (27.23) | 4,050 (26.95) |
| Main Bronchus | 346 (1.65) | 58 (1.36) | 73 (1.60) | 136 (1.74) | 79 (1.84) | 89 (1.50) | 257 (1.71) |
| Only one side involved, right or left | 10 (0.05) |  | 5 (0.11) | 5 (0.06) |  |  | 10 (0.07) |
| Paired site midline tumor | 2 (0.01) | 1 (0.02) |  |  | 1 (0.02) | 1 (0.02) | 1 (0.01) |
| Right Lower Lobe | 3,637 (17.36) | 744 (17.44) | 798 (17.48) | 1,366 (17.43) | 729 (17.01) | 1,019 (17.20) | 2,618 (17.42) |
| Right Lung, NOS | 332 (1.58) | 74 (1.73) | 80 (1.75) | 115 (1.47) | 63 (1.47) | 106 (1.79) | 226 (1.50) |
| Right Middle Lobe | 917 (4.38) | 188 (4.41) | 190 (4.16) | 351 (4.48) | 188 (4.39) | 257 (4.34) | 660 (4.39) |
| Right Overlapping Lesion of Lung | 454 (2.17) | 92 (2.16) | 106 (2.32) | 171 (2.18) | 85 (1.98) | 130 (2.19) | 324 (2.16) |
| Right Upper Lobe | 6,029 (28.78) | 1,232 (28.88) | 1,289 (28.23) | 2,259 (28.83) | 1,249 (29.15) | 1,691 (28.55) | 4,338 (28.86) |
| Missing | 17 (0.08) | 4 (0.09) | 5 (0.11) | 6 (0.08) | 2 (0.05) | 6 (0.10) | 11 (0.07) |
| Overall stage |  |  |  |  |  |  |  |
| 0 | 4 (0.02) | 1 (0.02) | 2 (0.04) |  | 1 (0.02) | 2 (0.03) | 2 (0.01) |
| 1 | 233 (1.11) | 50 (1.17) | 56 (1.23) | 96 (1.23) | 31 (0.72) | 71 (1.20) | 162 (1.08) |
| 2 | 9,480 (45.25) | 1,916 (44.91) | 2,087 (45.71) | 3,531 (45.07) | 1,946 (45.41) | 2,690 (45.42) | 6,790 (45.18) |
| 3 | 11,127 (53.11) | 2,249 (52.72) | 2,396 (52.47) | 4,189 (53.47) | 2,293 (53.51) | 3,101 (52.36) | 8,026 (53.40) |
| 4 | 32 (0.15) | 16 (0.38) | 5 (0.11) | 5 (0.06) | 6 (0.14) | 16 (0.27) | 16 (0.11) |
| Occult | 1 (0.00) | 1 (0.02) |  |  |  | 1 (0.02) |  |
| Missing | 75 (0.36) | 33 (0.77) | 20 (0.44) | 14 (0.18) | 8 (0.19) | 42 (0.71) | 33 (0.22) |
| **Hospital variables** | | | | | | | |
| Facility type |  |  |  |  |  |  |  |
| Academic/Research Program | 7,926 (37.83) | 1,494 (35.02) | 1,977 (43.30) | 3,655 (46.65) | 800 (18.67) | 2,227 (37.60) | 5,699 (37.92) |
| Community Cancer Program | 1,560 (7.45) | 455 (10.67) | 225 (4.93) | 106 (1.35) | 774 (18.06) | 540 (9.12) | 1,020 (6.79) |
| Comprehensive Community Cancer Program | 9,000 (42.96) | 1,958 (45.90) | 1,987 (43.52) | 2,968 (37.88) | 2,087 (48.70) | 2,663 (44.96) | 6,337 (42.17) |
| Integrated Network Cancer Program | 2,352 (11.23) | 341 (7.99) | 354 (7.75) | 1,055 (13.47) | 602 (14.05) | 466 (7.87) | 1,886 (12.55) |
| Missing | 114 (0.54) | 18 (0.42) | 23 (0.50) | 51 (0.65) | 22 (0.51) | 27 (0.46) | 87 (0.58) |
| Geographic location |  |  |  |  |  |  |  |
| East North Central | 3,889 (18.56) | 329 (7.71) | 905 (19.82) | 1,541 (19.67) | 1,114 (26.00) | 603 (10.18) | 3,286 (21.86) |
| East South Central | 1,845 (8.81) | 496 (11.63) | 371 (8.13) | 716 (9.14) | 262 (6.11) | 655 (11.06) | 1,190 (7.92) |
| Middle Atlantic | 3,409 (16.27) | 1,057 (24.78) | 602 (13.18) | 1,225 (15.63) | 525 (12.25) | 1,297 (21.90) | 2,112 (14.05) |
| Mountain | 639 (3.05) | 154 (3.61) | 229 (5.02) | 85 (1.08) | 171 (3.99) | 250 (4.22) | 389 (2.59) |
| New England | 1,286 (6.14) | 191 (4.48) | 293 (6.42) | 511 (6.52) | 291 (6.79) | 321 (5.42) | 965 (6.42) |
| Pacific | 1,889 (9.02) | 659 (15.45) | 568 (12.44) | 386 (4.93) | 276 (6.44) | 789 (13.32) | 1,100 (7.32) |
| South Atlantic | 4,849 (23.14) | 589 (13.81) | 1,140 (24.97) | 2,176 (27.77) | 944 (22.03) | 946 (15.97) | 3,903 (25.97) |
| West North Central | 1,617 (7.72) | 173 (4.06) | 239 (5.23) | 853 (10.89) | 352 (8.21) | 317 (5.35) | 1,300 (8.65) |
| West South Central | 1,415 (6.75) | 600 (14.06) | 196 (4.29) | 291 (3.71) | 328 (7.65) | 718 (12.12) | 697 (4.64) |
| Missing | 3,889 (18.56) | 329 (7.71) | 905 (19.82) | 1,541 (19.67) | 1,114 (26.00) | 603 (10.18) | 3,286 (21.86) |
| Number of reporting facilities |  |  |  |  |  |  |  |
| Single | 15,704 (74.95) | 3,358 (78.72) | 3,549 (77.73) | 5,798 (74.00) | 2,999 (69.99) | 4,661 (78.69) | 11,043 (73.48) |
| Multiple | 5,248 (25.05) | 908 (21.28) | 1,017 (22.27) | 2,037 (26.00) | 1,286 (30.01) | 1,262 (21.31) | 3,986 (26.52) |
| Travel distance in miles, median (IQR) | 11.4 (4.9-27.9) | 9.5 (4.3-23.0) | 10.4 (4.7-26.3) | 14.9 (6.2-35.2) | 9.7 (4.3-22.1) | 9.7 (4.4-24.1) | 12.1 (5.3-29.3) |
| Number of patients treated, median (IQR) | 28.0 (15.0-53.0) | 23.0 (14.0-54.0) | 27.0 (17.0-42.0) | 41.0 (26.0-82.0) | 15.0 (7.0-24.0) | 26.0 (14.0-52.0) | 29.0 (16.0-55.0) |
| **Treatment variables** | | | | | | | |
| Extent of Surgery |  |  |  |  |  |  |  |
| Sublobar Resection | 1,325 (6.32) | 275 (6.45) | 285 (6.24) | 453 (5.78) | 312 (7.28) | 376 (6.35) | 949 (6.31) |
| Lobectomy | 15,496 (73.96) | 3,144 (73.70) | 3,363 (73.65) | 5,874 (74.97) | 3,115 (72.70) | 4,364 (73.68) | 11,132 (74.07) |
| Pneumonectomy | 2,804 (13.38) | 506 (11.86) | 640 (14.02) | 1,074 (13.71) | 584 (13.63) | 740 (12.49) | 2,064 (13.73) |
| Other | 1,327 (6.33) | 341 (7.99) | 278 (6.09) | 434 (5.54) | 274 (6.39) | 443 (7.48) | 884 (5.88) |
| Use of radiation therapy |  |  |  |  |  |  |  |
| No | 14,693 (70.13) | 3,090 (72.43) | 3,284 (71.92) | 5,545 (70.77) | 2,774 (64.74) | 4,285 (72.35) | 10,408 (69.25) |
| Yes | 6,082 (29.03) | 1,092 (25.60) | 1,247 (27.31) | 2,266 (28.92) | 1,477 (34.47) | 1,539 (25.98) | 4,543 (30.23) |
| Missing | 177 (0.84) | 84 (1.97) | 35 (0.77) | 24 (0.31) | 34 (0.79) | 99 (1.67) | 78 (0.52) |
| Use of chemotherapy |  |  |  |  |  |  |  |
| No | 4,111 (19.62) | 1,264 (29.63) | 1,021 (22.36) | 1,306 (16.67) | 520 (12.14) | 1,664 (28.09) | 2,447 (16.28) |
| Yes | 16,159 (77.12) | 2,769 (64.91) | 3,388 (74.20) | 6,328 (80.77) | 3,674 (85.74) | 3,968 (66.99) | 12,191 (81.12) |
| Missing | 682 (3.26) | 233 (5.46) | 157 (3.44) | 201 (2.57) | 91 (2.12) | 291 (4.91) | 391 (2.60) |
| Use of immunotherapy |  |  |  |  |  |  |  |
| No | 20,827 (99.40) | 4,233 (99.23) | 4,546 (99.56) | 7,788 (99.40) | 4,260 (99.42) | 5,883 (99.32) | 14,944 (99.43) |
| Yes | 62 (0.30) | 5 (0.12) | 14 (0.31) | 31 (0.40) | 12 (0.28) | 9 (0.15) | 53 (0.35) |
| Missing | 63 (0.30) | 28 (0.66) | 6 (0.13) | 16 (0.20) | 13 (0.30) | 31 (0.52) | 32 (0.21) |

Abbreviations: EPR-expected performance rate, SD-standard deviation, mm-millimeter, IQR-interquartile range, NOS-not otherwise specified

Table S8. Patient data for all eligible RECRTCT patients

| **Variable** | **Overall** | **Quartile 1** | **Quartile 2** | **Quartile 3** | **Quartile 4** | **Below EPR** | **Above EPR** |
| --- | --- | --- | --- | --- | --- | --- | --- |
| **Demographic variables** | | | | | | | |
| Age, mean (SD) | 59.0 (11.0) | 59.0 (11.1) | 59.0 (11.1) | 58.0 (11.1) | 59.0 (10.7) | 59.0 (11.1) | 59.0 (11.0) |
| Sex |  |  |  |  |  |  |  |
| Female | 10,259 (37.54) | 1,735 (37.06) | 2,912 (38.13) | 4,030 (37.87) | 1,582 (36.25) | 5,361 (38.08) | 4,898 (36.97) |
| Male | 17,066 (62.46) | 2,947 (62.94) | 4,726 (61.87) | 6,611 (62.13) | 2,782 (63.75) | 8,716 (61.92) | 8,350 (63.03) |
| Race |  |  |  |  |  |  |  |
| White | 23,437 (85.77) | 3,801 (81.18) | 6,428 (84.16) | 9,310 (87.49) | 3,898 (89.32) | 11,762 (83.55) | 11,675 (88.13) |
| Black | 2,215 (8.11) | 436 (9.31) | 688 (9.01) | 792 (7.44) | 299 (6.85) | 1,263 (8.97) | 952 (7.19) |
| Other | 1,501 (5.49) | 392 (8.37) | 472 (6.18) | 490 (4.60) | 147 (3.37) | 942 (6.69) | 559 (4.22) |
| Unknown | 172 (0.63) | 53 (1.13) | 50 (0.65) | 49 (0.46) | 20 (0.46) | 110 (0.78) | 62 (0.47) |
| Insurance Status |  |  |  |  |  |  |  |
| Private Insurance | 14,533 (53.19) | 2,436 (52.03) | 3,971 (51.99) | 5,880 (55.26) | 2,246 (51.47) | 7,372 (52.37) | 7,161 (54.05) |
| Medicaid | 2,122 (7.77) | 411 (8.78) | 611 (8.00) | 792 (7.44) | 308 (7.06) | 1,174 (8.34) | 948 (7.16) |
| Medicare | 8,310 (30.41) | 1,477 (31.55) | 2,249 (29.44) | 3,144 (29.55) | 1,440 (33.00) | 4,262 (30.28) | 4,048 (30.56) |
| Other Government | 392 (1.43) | 62 (1.32) | 104 (1.36) | 145 (1.36) | 81 (1.86) | 188 (1.34) | 204 (1.54) |
| Not Insured | 1,433 (5.24) | 228 (4.87) | 423 (5.54) | 559 (5.25) | 223 (5.11) | 718 (5.10) | 715 (5.40) |
| Unknown | 535 (1.96) | 68 (1.45) | 280 (3.67) | 121 (1.14) | 66 (1.51) | 363 (2.58) | 172 (1.30) |
| Education (% without high school degree) |  |  |  |  |  |  |  |
| <7% | 6,358 (23.27) | 979 (20.91) | 1,825 (23.89) | 2,548 (23.95) | 1,006 (23.05) | 3,283 (23.32) | 3,075 (23.21) |
| 7.0-12.9% | 8,874 (32.48) | 1,353 (28.90) | 2,426 (31.76) | 3,527 (33.15) | 1,568 (35.93) | 4,400 (31.26) | 4,474 (33.77) |
| 13-20.9% | 7,257 (26.56) | 1,244 (26.57) | 2,004 (26.24) | 2,863 (26.91) | 1,146 (26.26) | 3,649 (25.92) | 3,608 (27.23) |
| >=21% | 4,784 (17.51) | 1,096 (23.41) | 1,371 (17.95) | 1,680 (15.79) | 637 (14.60) | 2,717 (19.30) | 2,067 (15.60) |
| Missing | 52 (0.19) | 10 (0.21) | 12 (0.16) | 23 (0.22) | 7 (0.16) | 28 (0.20) | 24 (0.18) |
| Charlson Deyo comorbidity score |  |  |  |  |  |  |  |
| 0 | 21,530 (78.79) | 3,710 (79.24) | 5,985 (78.36) | 8,421 (79.14) | 3,414 (78.23) | 11,099 (78.84) | 10,431 (78.74) |
| 1 | 4,631 (16.95) | 786 (16.79) | 1,309 (17.14) | 1,774 (16.67) | 762 (17.46) | 2,373 (16.86) | 2,258 (17.04) |
| 2 | 866 (3.17) | 143 (3.05) | 260 (3.40) | 323 (3.04) | 140 (3.21) | 452 (3.21) | 414 (3.13) |
| >2 | 298 (1.09) | 43 (0.92) | 84 (1.10) | 123 (1.16) | 48 (1.10) | 153 (1.09) | 145 (1.09) |
| **Cancer variables** | | | | | | | |
| Size of tumor (mm), median (SD) | 40.0 (27.0-55.0) | 40.0 (26.0-55.0) | 40.0 (25.0-51.0) | 40.0 (27.0-55.0) | 40.0 (27.0-55.0) | 40.0 (26.0-54.0) | 40.0 (27.0-55.0) |
| Tumor Grade |  |  |  |  |  |  |  |
| Well differentiated | 1,999 (7.32) | 364 (7.77) | 550 (7.20) | 743 (6.98) | 342 (7.84) | 1,035 (7.35) | 964 (7.28) |
| Moderately differentiated | 18,540 (67.85) | 3,141 (67.09) | 5,182 (67.84) | 7,255 (68.18) | 2,962 (67.87) | 9,465 (67.24) | 9,075 (68.50) |
| Poorly differentiated | 2,682 (9.82) | 536 (11.45) | 723 (9.47) | 1,051 (9.88) | 372 (8.52) | 1,495 (10.62) | 1,187 (8.96) |
| Undifferentiated | 331 (1.21) | 63 (1.35) | 86 (1.13) | 132 (1.24) | 50 (1.15) | 174 (1.24) | 157 (1.19) |
| Unknown | 3,773 (13.81) | 578 (12.35) | 1,097 (14.36) | 1,460 (13.72) | 638 (14.62) | 1,908 (13.55) | 1,865 (14.08) |
| Lymph node status |  |  |  |  |  |  |  |
| Negative | 16,636 (60.88) | 2,642 (56.43) | 4,493 (58.82) | 6,657 (62.56) | 2,844 (65.17) | 8,216 (58.36) | 8,420 (63.56) |
| Positive | 8,461 (30.96) | 1,627 (34.75) | 2,457 (32.17) | 3,189 (29.97) | 1,188 (27.22) | 4,631 (32.90) | 3,830 (28.91) |
| Missing | 2,228 (8.15) | 413 (8.82) | 688 (9.01) | 795 (7.47) | 332 (7.61) | 1,230 (8.74) | 998 (7.53) |
| Overall stage |  |  |  |  |  |  |  |
| 0 | 768 (2.81) | 93 (1.99) | 207 (2.71) | 333 (3.13) | 135 (3.09) | 358 (2.54) | 410 (3.09) |
| 1 | 5,276 (19.31) | 693 (14.80) | 1,412 (18.49) | 2,177 (20.46) | 994 (22.78) | 2,448 (17.39) | 2,828 (21.35) |
| 2 | 9,504 (34.78) | 1,712 (36.57) | 2,640 (34.56) | 3,641 (34.22) | 1,511 (34.62) | 4,953 (35.19) | 4,551 (34.35) |
| 3 | 11,693 (42.79) | 2,153 (45.98) | 3,362 (44.02) | 4,465 (41.96) | 1,713 (39.25) | 6,262 (44.48) | 5,431 (40.99) |
| 4 | 39 (0.14) | 13 (0.28) | 11 (0.14) | 11 (0.10) | 4 (0.09) | 25 (0.18) | 14 (0.11) |
| Unknown | 45 (0.16) | 18 (0.38) | 6 (0.08) | 14 (0.13) | 7 (0.16) | 31 (0.22) | 14 (0.11) |
| **Hospital variables** | | | | | | | |
| Facility type |  |  |  |  |  |  |  |
| Academic/Research Program | 9,606 (35.15) | 1,502 (32.08) | 3,046 (39.88) | 4,224 (39.70) | 834 (19.11) | 5,363 (38.10) | 4,243 (32.03) |
| Community Cancer Program | 2,269 (8.30) | 562 (12.00) | 422 (5.53) | 503 (4.73) | 782 (17.92) | 1,150 (8.17) | 1,119 (8.45) |
| Comprehensive Community Cancer Program | 11,282 (41.29) | 2,202 (47.03) | 3,159 (41.36) | 3,905 (36.70) | 2,016 (46.20) | 5,861 (41.64) | 5,421 (40.92) |
| Integrated Network Cancer Program | 2,806 (10.27) | 185 (3.95) | 620 (8.12) | 1,439 (13.52) | 562 (12.88) | 980 (6.96) | 1,826 (13.78) |
| Missing | 1,362 (4.98) | 231 (4.93) | 391 (5.12) | 570 (5.36) | 170 (3.90) | 723 (5.14) | 639 (4.82) |
| Geographic location |  |  |  |  |  |  |  |
| East North Central | 4,888 (17.89) | 350 (7.48) | 1,603 (20.99) | 2,062 (19.38) | 873 (20.00) | 2,087 (14.83) | 2,801 (21.14) |
| East South Central | 1,712 (6.27) | 340 (7.26) | 425 (5.56) | 697 (6.55) | 250 (5.73) | 787 (5.59) | 925 (6.98) |
| Middle Atlantic | 3,612 (13.22) | 625 (13.35) | 968 (12.67) | 1,551 (14.58) | 468 (10.72) | 1,871 (13.29) | 1,741 (13.14) |
| Mountain | 1,295 (4.74) | 159 (3.40) | 532 (6.97) | 490 (4.60) | 114 (2.61) | 794 (5.64) | 501 (3.78) |
| New England | 1,385 (5.07) | 106 (2.26) | 336 (4.40) | 767 (7.21) | 176 (4.03) | 721 (5.12) | 664 (5.01) |
| Pacific | 3,082 (11.28) | 1,298 (27.72) | 776 (10.16) | 598 (5.62) | 410 (9.40) | 2,142 (15.22) | 940 (7.10) |
| South Atlantic | 5,298 (19.39) | 812 (17.34) | 1,185 (15.51) | 2,361 (22.19) | 940 (21.54) | 2,271 (16.13) | 3,027 (22.85) |
| West North Central | 2,506 (9.17) | 239 (5.10) | 463 (6.06) | 1,058 (9.94) | 746 (17.09) | 1,146 (8.14) | 1,360 (10.27) |
| West South Central | 2,185 (8.00) | 522 (11.15) | 959 (12.56) | 487 (4.58) | 217 (4.97) | 1,535 (10.90) | 650 (4.91) |
| Missing | 1,362 (4.98) | 231 (4.93) | 391 (5.12) | 570 (5.36) | 170 (3.90) | 723 (5.14) | 639 (4.82) |
| Number of reporting facilities |  |  |  |  |  |  |  |
| Single | 20,317 (74.35) | 3,766 (80.44) | 5,752 (75.31) | 7,815 (73.44) | 2,984 (68.38) | 10,785 (76.61) | 9,532 (71.95) |
| Multiple | 7,008 (25.65) | 916 (19.56) | 1,886 (24.69) | 2,826 (26.56) | 1,380 (31.62) | 3,292 (23.39) | 3,716 (28.05) |
| Travel distance in miles, median (range) | 11.9 (5.1-29.0) | 9.6 (4.3-23.0) | 11.7 (5.1-28.9) | 14.0 (5.9-32.8) | 10.3 (4.7-26.3) | 11.2 (4.8-27.4) | 12.9 (5.5-30.7) |
| Number of patients treated, median (IQR) | 36.0 (19.0-66.0) | 25.0 (13.0-43.0) | 38.0 (21.0-69.0) | 49.0 (26.0-74.0) | 23.0 (12.0-42.0) | 33.0 (19.0-63.0) | 41.0 (19.5-68.0) |
| **Treatment variables** | | | | | | | |
| Extent of Surgery |  |  |  |  |  |  |  |
| Segmental Resection | 16,632 (60.87) | 2,885 (61.62) | 4,680 (61.27) | 6,475 (60.85) | 2,592 (59.40) | 8,587 (61.00) | 8,045 (60.73) |
| Coloanal Anastomosis | 2,048 (7.49) | 226 (4.83) | 606 (7.93) | 870 (8.18) | 346 (7.93) | 977 (6.94) | 1,071 (8.08) |
| Total proctectomy | 6,339 (23.20) | 1,124 (24.01) | 1,586 (20.76) | 2,480 (23.31) | 1,149 (26.33) | 3,166 (22.49) | 3,173 (23.95) |
| Total proctocolectomy | 656 (2.40) | 122 (2.61) | 181 (2.37) | 254 (2.39) | 99 (2.27) | 338 (2.40) | 318 (2.40) |
| Surgery, NOS | 1,650 (6.04) | 325 (6.94) | 585 (7.66) | 562 (5.28) | 178 (4.08) | 1,009 (7.17) | 641 (4.84) |
| Use of radiation therapy |  |  |  |  |  |  |  |
| No | 2,268 (8.30) | 687 (14.67) | 758 (9.92) | 721 (6.78) | 102 (2.34) | 1,595 (11.33) | 673 (5.08) |
| Yes | 25,023 (91.58) | 3,985 (85.11) | 6,863 (89.85) | 9,914 (93.17) | 4,261 (97.64) | 12,454 (88.47) | 12,569 (94.87) |
| Missing | 34 (0.12) | 10 (0.21) | 17 (0.22) | 6 (0.06) | 1 (0.02) | 28 (0.20) | 6 (0.05) |
| Use of chemotherapy |  |  |  |  |  |  |  |
| No | 156 (0.57) | 57 (1.22) | 54 (0.71) | 39 (0.37) | 6 (0.14) | 125 (0.89) | 31 (0.23) |
| Yes | 1,486 (5.44) | 484 (10.34) | 475 (6.22) | 449 (4.22) | 78 (1.79) | 1,058 (7.52) | 428 (3.23) |
| Missing | 25,683 (93.99) | 4,141 (88.45) | 7,109 (93.07) | 10,153 (95.41) | 4,280 (98.08) | 12,894 (91.60) | 12,789 (96.54) |

Abbreviations: EPR-expected performance rate, SD-standard deviation, mm-millimeter, IQR-interquartile range, NOS-not otherwise specified

Table S9. Patient data for all eligible G15RLN patients

| **Variable** | **Overall** | **Quartile 1** | **Quartile 2** | **Quartile 3** | **Quartile 4** | **Below EPR** | **Above EPR** |
| --- | --- | --- | --- | --- | --- | --- | --- |
| **Demographic variables** | | | | | | | |
| Age, mean (SD) | 69.0 (13.3) | 72.0 (13.0) | 70.0 (13.0) | 69.0 (13.3) | 67.0 (13.4) | 69.0 (13.2) | 66.0 (13.7) |
| Sex |  |  |  |  |  |  |  |
| Female | 6,264 (43.81) | 608 (45.65) | 1,372 (43.04) | 2,077 (44.14) | 2,207 (43.51) | 5,630 (43.73) | 634 (44.52) |
| Male | 8,033 (56.19) | 724 (54.35) | 1,816 (56.96) | 2,628 (55.86) | 2,865 (56.49) | 7,243 (56.27) | 790 (55.48) |
| Race |  |  |  |  |  |  |  |
| White | 9,015 (63.06) | 935 (70.20) | 2,000 (62.74) | 2,983 (63.40) | 3,097 (61.06) | 8,171 (63.47) | 844 (59.27) |
| Black | 2,976 (20.82) | 281 (21.10) | 836 (26.22) | 1,006 (21.38) | 853 (16.82) | 2,782 (21.61) | 194 (13.62) |
| Other | 2,162 (15.12) | 108 (8.11) | 318 (9.97) | 683 (14.52) | 1,053 (20.76) | 1,799 (13.97) | 363 (25.49) |
| Unknown | 144 (1.01) | 8 (0.60) | 34 (1.07) | 33 (0.70) | 69 (1.36) | 121 (0.94) | 23 (1.62) |
| Insurance Status |  |  |  |  |  |  |  |
| Private Insurance | 4,328 (30.27) | 342 (25.68) | 875 (27.45) | 1,412 (30.01) | 1,699 (33.50) | 3,783 (29.39) | 545 (38.27) |
| Medicaid | 1,163 (8.13) | 85 (6.38) | 219 (6.87) | 369 (7.84) | 490 (9.66) | 1,017 (7.90) | 146 (10.25) |
| Medicare | 7,896 (55.23) | 845 (63.44) | 1,868 (58.59) | 2,577 (54.77) | 2,606 (51.38) | 7,225 (56.13) | 671 (47.12) |
| Other Government | 137 (0.96) | 8 (0.60) | 48 (1.51) | 48 (1.02) | 33 (0.65) | 132 (1.03) | 5 (0.35) |
| Not Insured | 525 (3.67) | 43 (3.23) | 120 (3.76) | 183 (3.89) | 179 (3.53) | 494 (3.84) | 31 (2.18) |
| Unknown | 331 (1.86) | 24 (1.25) | 57 (1.51) | 65 (1.30) | 185 (2.61) | 326 (1.93) | 5 (0.54) |
| Education (% without high school degree) |  |  |  |  |  |  |  |
| <7% | 2,739 (19.16) | 229 (17.19) | 531 (16.66) | 933 (19.83) | 1,046 (20.62) | 2,455 (19.07) | 284 (19.94) |
| 7.0-12.9% | 4,000 (27.98) | 371 (27.85) | 845 (26.51) | 1,298 (27.59) | 1,486 (29.30) | 3,580 (27.81) | 420 (29.49) |
| 13-20.9% | 3,842 (26.87) | 401 (30.11) | 962 (30.18) | 1,258 (26.74) | 1,221 (24.07) | 3,513 (27.29) | 329 (23.10) |
| >=21% | 3,661 (25.61) | 326 (24.47) | 843 (26.44) | 1,197 (25.44) | 1,295 (25.53) | 3,274 (25.43) | 387 (27.18) |
| Missing | 55 (0.38) | 5 (0.38) | 7 (0.22) | 19 (0.40) | 24 (0.47) | 51 (0.40) | 4 (0.28) |
| Charlson Deyo comorbidity score |  |  |  |  |  |  |  |
| 0 | 9,150 (64.00) | 812 (60.96) | 1,957 (61.39) | 2,957 (62.85) | 3,424 (67.51) | 8,148 (63.30) | 1,002 (70.37) |
| 1 | 3,708 (25.94) | 365 (27.40) | 858 (26.91) | 1,247 (26.50) | 1,238 (24.41) | 3,366 (26.15) | 342 (24.02) |
| 2 | 1,027 (7.18) | 114 (8.56) | 262 (8.22) | 355 (7.55) | 296 (5.84) | 973 (7.56) | 54 (3.79) |
| >2 | 412 (2.88) | 41 (3.08) | 111 (3.48) | 146 (3.10) | 114 (2.25) | 386 (3.00) | 26 (1.83) |
| **Cancer variables** | | | | | | | |
| Tumor size (mm), median (IQR) | 40.0 (22.0-60.0) | 38.0 (20.0-55.0) | 40.0 (23.0-60.0) | 40.0 (22.0-60.0) | 40.0 (21.0-60.0) | 40.0 (22.0-60.0) | 37.0 (20.0-60.0) |
| Tumor Grade |  |  |  |  |  |  |  |
| Well differentiated | 1,054 (7.37) | 108 (8.11) | 298 (9.35) | 357 (7.59) | 291 (5.74) | 976 (7.58) | 78 (5.48) |
| Moderately differentiated | 3,447 (24.11) | 395 (29.65) | 804 (25.22) | 1,075 (22.85) | 1,173 (23.13) | 3,124 (24.27) | 323 (22.68) |
| Poorly differentiated | 8,673 (60.66) | 721 (54.13) | 1,841 (57.75) | 2,899 (61.62) | 3,212 (63.33) | 7,761 (60.29) | 912 (64.04) |
| Undifferentiated | 275 (1.92) | 22 (1.65) | 63 (1.98) | 65 (1.38) | 125 (2.46) | 237 (1.84) | 38 (2.67) |
| Unknown | 848 (5.93) | 86 (6.46) | 182 (5.71) | 309 (6.57) | 271 (5.34) | 775 (6.02) | 73 (5.13) |
| Lymph node status |  |  |  |  |  |  |  |
| Negative | 6,292 (44.01) | 580 (43.54) | 1,326 (41.59) | 2,054 (43.66) | 2,332 (45.98) | 5,606 (43.55) | 686 (48.17) |
| Positive | 7,201 (50.37) | 592 (44.44) | 1,620 (50.82) | 2,379 (50.56) | 2,610 (51.46) | 6,487 (50.39) | 714 (50.14) |
| Missing | 804 (5.62) | 160 (12.01) | 242 (7.59) | 272 (5.78) | 130 (2.56) | 780 (6.06) | 24 (1.69) |
| Overall stage |  |  |  |  |  |  |  |
| 0 | 233 (1.63) | 20 (1.50) | 48 (1.51) | 86 (1.83) | 79 (1.56) | 210 (1.63) | 23 (1.62) |
| 1 | 4,762 (33.31) | 472 (35.44) | 1,020 (31.99) | 1,526 (32.43) | 1,744 (34.38) | 4,249 (33.01) | 513 (36.03) |
| 2 | 4,014 (28.08) | 426 (31.98) | 958 (30.05) | 1,318 (28.01) | 1,312 (25.87) | 3,651 (28.36) | 363 (25.49) |
| 3 | 5,288 (36.99) | 414 (31.08) | 1,162 (36.45) | 1,775 (37.73) | 1,937 (38.19) | 4,763 (37.00) | 525 (36.87) |
| **Hospital variables** | | | | | | | |
| Facility type |  |  |  |  |  |  |  |
| Academic/Research Program | 6,237 (43.62) | 193 (14.49) | 839 (26.32) | 2,031 (43.17) | 3,174 (62.58) | 5,272 (40.95) | 965 (67.77) |
| Community Cancer Program | 1,057 (7.39) | 299 (22.45) | 302 (9.47) | 257 (5.46) | 199 (3.92) | 975 (7.57) | 82 (5.76) |
| Comprehensive Community Cancer Program | 5,061 (35.40) | 705 (52.93) | 1,730 (54.27) | 1,527 (32.45) | 1,099 (21.67) | 4,831 (37.53) | 230 (16.15) |
| Integrated Network Cancer Program | 1,512 (10.58) | 105 (7.88) | 234 (7.34) | 737 (15.66) | 436 (8.60) | 1,423 (11.05) | 89 (6.25) |
| Missing | 430 (3.01) | 30 (2.25) | 83 (2.60) | 153 (3.25) | 164 (3.23) | 372 (2.89) | 58 (4.07) |
| Geographic location |  |  |  |  |  |  |  |
| East North Central | 2,052 (14.35) | 202 (15.17) | 464 (14.55) | 755 (16.05) | 631 (12.44) | 1,965 (15.26) | 87 (6.11) |
| East South Central | 851 (5.95) | 196 (14.71) | 294 (9.22) | 271 (5.76) | 90 (1.77) | 819 (6.36) | 32 (2.25) |
| Middle Atlantic | 2,813 (19.68) | 147 (11.04) | 390 (12.23) | 728 (15.47) | 1,548 (30.52) | 2,191 (17.02) | 622 (43.68) |
| Mountain | 404 (2.83) | 91 (6.83) | 103 (3.23) | 126 (2.68) | 84 (1.66) | 400 (3.11) | 4 (0.28) |
| New England | 729 (5.10) | 46 (3.45) | 112 (3.51) | 269 (5.72) | 302 (5.95) | 671 (5.21) | 58 (4.07) |
| Pacific | 1,988 (13.91) | 97 (7.28) | 339 (10.63) | 491 (10.44) | 1,061 (20.92) | 1,660 (12.90) | 328 (23.03) |
| South Atlantic | 2,987 (20.89) | 259 (19.44) | 915 (28.70) | 1,137 (24.17) | 676 (13.33) | 2,867 (22.27) | 120 (8.43) |
| West North Central | 693 (4.85) | 144 (10.81) | 124 (3.89) | 207 (4.40) | 218 (4.30) | 643 (4.99) | 50 (3.51) |
| West South Central | 12,107 (84.68) | 1,191 (89.41) | 2,782 (87.26) | 3,994 (84.89) | 4,140 (81.62) | 10,978 (85.28) | 1,129 (79.28) |
| Missing | 430 (3.01) | 30 (2.25) | 83 (2.60) | 153 (3.25) | 164 (3.23) | 372 (2.89) | 58 (4.07) |
| Number of reporting facilities |  |  |  |  |  |  |  |
| Single | 15,218 (85.52) | 1,736 (90.28) | 3,299 (87.46) | 4,262 (85.09) | 5,921 (83.50) | 14,446 (85.62) | 772 (83.64) |
| Multiple | 2,190 (15.32) | 141 (10.59) | 406 (12.74) | 711 (15.11) | 932 (18.38) | 1,895 (14.72) | 295 (20.72) |
| Travel distance in miles, median (IQR) | 8.6 (3.9-21.5) | 7.3 (3.3-17.2) | 7.6 (3.7-17.0) | 9.2 (4.2-23.4) | 9.4 (4.1-25.0) | 8.4 (3.9-20.8) | 11.5 (4.6-28.4) |
| Number of patients treated, median (IQR) | 23.0 (12.0-46.0) | 8.0 (5.0-12.0) | 15.0 (10.0-24.0) | 25.0 (14.0-46.0) | 38.0 (21.0-60.0) | 22.0 (11.0-39.0) | 49.0 (21.0-103.0) |
| **Treatment variables** | | | | | | | |
| Extent of surgery |  |  |  |  |  |  |  |
| Partial gastrectomy | 10,505 (73.48) | 1,127 (84.61) | 2,524 (79.17) | 3,348 (71.16) | 3,506 (69.12) | 9,532 (74.05) | 973 (68.33) |
| Total gastrectomy | 3,383 (23.66) | 179 (13.44) | 582 (18.26) | 1,194 (25.38) | 1,428 (28.15) | 2,958 (22.98) | 425 (29.85) |
| Gastrectomy, NOS | 409 (2.86) | 26 (1.95) | 82 (2.57) | 163 (3.46) | 138 (2.72) | 383 (2.98) | 26 (1.83) |
| Use of radiation therapy |  |  |  |  |  |  |  |
| No | 10,959 (76.65) | 1,021 (76.65) | 2,437 (76.44) | 3,520 (74.81) | 3,981 (78.49) | 9,835 (76.40) | 1,124 (78.93) |
| Yes | 3,215 (22.49) | 290 (21.77) | 738 (23.15) | 1,152 (24.48) | 1,035 (20.41) | 2,946 (22.89) | 269 (18.89) |
| Missing | 123 (0.86) | 21 (1.58) | 13 (0.41) | 33 (0.70) | 56 (1.10) | 92 (0.71) | 31 (2.18) |
| Use of chemotherapy |  |  |  |  |  |  |  |
| No | 7,167 (50.13) | 769 (57.73) | 1,740 (54.58) | 2,346 (49.86) | 2,312 (45.58) | 6,560 (50.96) | 607 (42.63) |
| Yes | 6,529 (45.67) | 490 (36.79) | 1,346 (42.22) | 2,153 (45.76) | 2,540 (50.08) | 5,766 (44.79) | 763 (53.58) |
| Missing | 601 (4.20) | 73 (5.48) | 102 (3.20) | 206 (4.38) | 220 (4.34) | 547 (4.25) | 54 (3.79) |
| Number of lymph nodes examined, median (IQR) | 15.0 (8.0-23.0) | 7.0 (3.0-11.0) | 11.0 (5.0-17.0) | 15.0 (9.0-23.0) | 20.0 (15.0-29.0) | 14.0 (8.0-22.0) | 23.0 (17.0-32.0) |

Abbreviations: EPR-expected performance rate, SD-standard deviation, mm-millimeter, IQR-interquartile range, NOS-not otherwise specified

Table S10. Unadjusted patient-level overall survival estimates based on compliance

|  |  | **Non-Compliant Patients** | | | **Compliant Patients** | | |
| --- | --- | --- | --- | --- | --- | --- | --- |
| **Measure** | **Timing** | **Survival**  **Probability** | **95% CI**  **Lower Limit** | **95% CI**  **Upper Limit** | **Survival**  **Probability** | **95% CI**  **Lower Limit** | **95% CI**  **Upper Limit** |
| **BCSRT** | 2 Year | 97.5% | 97.3% | 97.7% | 99.2% | 99.2% | 99.3% |
|  | 5 Year | 90.3% | 89.8% | 90.9% | 95.6% | 95.4% | 95.7% |
|  |  |  |  |  |  |  |  |
| **HT** | 2 Year | 97.8% | 97.6% | 97.9% | 98.4% | 98.3% | 98.4% |
|  | 5 Year | 88.7% | 88.3% | 89.1% | 90.9% | 90.7% | 91.0% |
|  |  |  |  |  |  |  |  |
| **MASTRT** | 2 Year | 88.7% | 87.9% | 89.6% | 93.5% | 93.2% | 93.7% |
|  | 5 Year | 65.7% | 64.1% | 67.3% | 74.9% | 74.3% | 75.5% |
|  |  |  |  |  |  |  |  |
| **12RLN** | 2 Year | 78.4% | 77.8% | 78.9% | 84.5% | 84.4% | 84.7% |
|  | 5 Year | 59.9% | 59.0% | 60.8% | 67.7% | 67.4% | 68.0% |
|  |  |  |  |  |  |  |  |
| **LNoSurg** | 2 Year | 62.7% | 61.2% | 64.1% | 36.7% | 36.2% | 37.2% |
|  | 5 Year | 36.9% | 35.0% | 38.7% | 17.8% | 17.3% | 18.3% |
|  |  |  |  |  |  |  |  |
| **LCT** | 2 Year | 71.6% | 69.8% | 73.4% | 74.2% | 73.6% | 74.9% |
|  | 5 Year | 45.7% | 43.2% | 48.1% | 47.2% | 46.3% | 48.2% |
|  |  |  |  |  |  |  |  |
| **RECRTCT** | 2 Year | 90.6% | 89.8% | 91.4% | 93.0% | 92.7% | 93.4% |
|  | 5 Year | 73.1% | 71.5% | 74.7% | 77.7% | 76.9% | 78.4% |
|  |  |  |  |  |  |  |  |
| **G15RLN** | 2 Year | 65.9% | 64.7% | 67.0% | 67.5% | 66.4% | 68.6% |
|  | 5 Year | 46.0% | 44.5% | 47.5% | 48.9% | 47.4% | 50.3% |

Abbreviations: CI-confidence interval

Table S11. Unadjusted hospital-level survival based on quartile group

|  |  | **2-Year Survival** | | | **5-Year Survival** | | |
| --- | --- | --- | --- | --- | --- | --- | --- |
| **Measure** | **Quartile**  **Group** | **Survival**  **Probability** | **95% CI**  **Lower Limit** | **95% CI**  **Upper Limit** | **Survival**  **Probability** | **95% CI**  **Lower Limit** | **95% CI**  **Upper Limit** |
| **BCSRT** | 1 | 99.1% | 99.0% | 99.2% | 95.3% | 95.0% | 95.5% |
|  | 2 | 99.0% | 98.9% | 99.1% | 94.9% | 94.7% | 95.1% |
|  | 3 | 99.1% | 99.0% | 99.1% | 95.0% | 94.8% | 95.2% |
|  | 4 | 99.1% | 99.0% | 99.2% | 95.4% | 95.2% | 95.6% |
| **HT** | 1 | 98.3% | 98.2% | 98.4% | 91.0% | 90.7% | 91.3% |
|  | 2 | 98.3% | 98.2% | 98.3% | 90.5% | 90.2% | 90.7% |
|  | 3 | 98.3% | 98.2% | 98.4% | 90.6% | 90.3% | 90.8% |
|  | 4 | 98.3% | 98.2% | 98.4% | 90.4% | 90.1% | 90.7% |
| **MASTRT** | 1 | 92.3% | 91.7% | 92.9% | 73.5% | 72.3% | 74.8% |
|  | 2 | 92.4% | 91.9% | 92.9% | 72.1% | 70.9% | 73.2% |
|  | 3 | 93.1% | 92.6% | 93.5% | 74.2% | 73.2% | 75.2% |
|  | 4 | 93.4% | 92.8% | 94.0% | 74.6% | 73.3% | 76.0% |
| **12RLN** | 1 | 81.6% | 81.2% | 82.0% | 62.8% | 62.1% | 63.5% |
|  | 2 | 83.1% | 82.7% | 83.4% | 65.9% | 65.3% | 66.5% |
|  | 3 | 84.1% | 83.8% | 84.4% | 67.3% | 66.7% | 67.8% |
|  | 4 | 85.7% | 85.4% | 86.0% | 69.8% | 69.2% | 70.3% |
| **LNoSurg** | 1 | 42.2% | 41.3% | 43.1% | 21.7% | 20.8% | 22.7% |
|  | 2 | 39.9% | 39.1% | 40.7% | 20.8% | 19.9% | 21.6% |
|  | 3 | 36.9% | 36.1% | 37.8% | 17.6% | 16.8% | 18.5% |
|  | 4 | 35.3% | 33.9% | 36.7% | 16.2% | 14.7% | 17.6% |
| **LCT** | 1 | 74.4% | 73.0% | 75.7% | 48.0% | 46.0% | 50.0% |
|  | 2 | 73.6% | 72.3% | 74.9% | 46.4% | 44.5% | 48.4% |
|  | 3 | 74.5% | 73.5% | 75.4% | 47.7% | 46.2% | 49.1% |
|  | 4 | 72.9% | 71.5% | 74.3% | 45.6% | 43.6% | 47.6% |
| **RECRTCT** | 1 | 91.4% | 90.6% | 92.2% | 74.8% | 73.1% | 76.5% |
|  | 2 | 92.9% | 92.3% | 93.5% | 76.8% | 75.5% | 78.1% |
|  | 3 | 92.8% | 92.3% | 93.3% | 78.0% | 76.9% | 79.0% |
|  | 4 | 92.7% | 91.9% | 93.5% | 75.8% | 74.0% | 77.6% |
| **G15RLN** | 1 | 60.3% | 57.6% | 63.0% | 39.6% | 36.3% | 42.9% |
|  | 2 | 61.7% | 59.9% | 63.4% | 42.1% | 39.9% | 44.3% |
|  | 3 | 66.9% | 65.5% | 68.3% | 47.7% | 45.9% | 49.6% |
|  | 4 | 71.5% | 70.2% | 72.8% | 52.8% | 51.0% | 54.6% |

Table S12. Unadjusted hospital-level survival based on EPR group

|  |  | **Below EPR** | | | **Above EPR** | | |
| --- | --- | --- | --- | --- | --- | --- | --- |
| **Measure** | **Timing** | **Survival**  **Probability** | **95% CI**  **Lower Limit** | **95% CI**  **Upper Limit** | **Survival**  **Probability** | **95% CI**  **Lower Limit** | **95% CI**  **Upper Limit** |
| **BCSRT** | 2 Year | 99.1% | 99.0% | 99.1% | 99.1% | 99.0% | 99.1% |
|  | 5 Year | 95.2% | 94.9% | 95.4% | 95.1% | 95.0% | 95.2% |
|  |  |  |  |  |  |  |  |
| **HT** | 2 Year | 98.3% | 98.2% | 98.3% | 98.3% | 98.3% | 98.4% |
|  | 5 Year | 90.7% | 90.5% | 90.9% | 90.5% | 90.3% | 90.7% |
|  |  |  |  |  |  |  |  |
| **MASTRT** | 2 Year | 92.4% | 92.1% | 92.8% | 93.2% | 92.8% | 93.6% |
|  | 5 Year | 72.9% | 72.1% | 73.7% | 74.4% | 73.5% | 75.2% |
|  |  |  |  |  |  |  |  |
| **12RLN** | 2 Year | 81.8% | 81.5% | 82.2% | 84.5% | 84.3% | 84.6% |
|  | 5 Year | 63.1% | 62.5% | 63.8% | 67.9% | 67.6% | 68.3% |
|  |  |  |  |  |  |  |  |
| **LNoSurg** | 2 Year | 42.1% | 40.9% | 43.3% | 38.6% | 38.1% | 39.1% |
|  | 5 Year | 22.6% | 21.4% | 23.9% | 19.0% | 18.5% | 19.5% |
|  |  |  |  |  |  |  |  |
| **LCT** | 2 Year | 74.1% | 72.9% | 75.2% | 73.9% | 73.2% | 74.6% |
|  | 5 Year | 47.7% | 46.0% | 49.3% | 46.8% | 45.8% | 47.9% |
|  |  |  |  |  |  |  |  |
| **RECRTCT** | 2 Year | 92.5% | 92.1% | 93.0% | 92.6% | 92.2% | 93.1% |
|  | 5 Year | 76.1% | 75.1% | 77.0% | 77.5% | 76.5% | 78.5% |
|  |  |  |  |  |  |  |  |
| **G15RLN** | 2 Year | 65.8% | 65.0% | 66.6% | 75.1% | 72.8% | 77.4% |
|  | 5 Year | 46.2% | 45.1% | 47.3% | 59.5% | 56.2% | 62.8% |

Abbreviations: CI-confidence interval

Supplementary Methods. Variables included in the multivariable Cox proportional hazards models

|  | HT | BCSRT | MASTRT | 12RLN | LNoSurg | LCT | RECRTCT | G15RLN |
| --- | --- | --- | --- | --- | --- | --- | --- | --- |
| Demographic and Clinicopathologic Variables |  |  |  |  |  |  |  |  |
| Age | I, II, III | I, II, III | I, II, III | I, II, III | I, II, III | I, II, III | I, II, III | I, II, III |
| Sex | N/A | N/A | N/A | I, II, III | I, II, III | I, II, III | I, II, III | I, II, III |
| Race | I, II, III | I, II, III | I, II, III | I, II, III | I, II, III | I, II, III | I, II, III | I, II, III |
| Insurance status | I, II, III | I, II, III | I, II, III | I, II, III | I, II, III | I, II, III | I, II, III | I, II, III |
| Education | I, II, III | I, II, III | I, II, III | I, II, III | I, II, III | I, II, III | I, II, III | I, II, III |
| Charlson Deyo comorbidity score | I, II, III | I, II, III | I, II, III | I, II, III | I, II, III | I, II, III | I, II, III | I, II, III |
| Sequence Number | I, II, III | I, II, III | I, II, III | I, II, III | I, II, III | I, II, III | I, II, III | I, II, III |
| Tumor grade | I, II, III | I, II, III | I, II, III | I, II, III | I, II, III | I, II, III | I, II, III | I, II, III |
| Lymph node status | I, II, III | I, II, III | N/A | I, II, III | N/A | I, II, III | I, II, III | I, II, III |
| Tumor size | I, II, III | I, II, III | I, II, III | I, II, III | I, II, III | I, II, III | I, II, III | I, II, III |
| Overall stage | I, II, III | I, II, III | I, II, III | I, II, III | I, II, III | I, II, III | I, II, III | I, II, III­­­­­ |
| Histologic subtype | N/A | N/A | N/A | N/A | E-CP | I, II, III | N/A | N/A |
| Hospital Variables |  |  |  |  |  |  |  |  |
| Facility type | II, III | II, III | II, III | II, III | II, III | II, III | II, III | II, III |
| Facility location | II, III | II, III | II, III | II, III | II, III | II, III | II, III | II, III |
| Number of reporting facilities | II, III | II, III | II, III | II, III | II, III | II, III | II, III | II, III |
| Treatment Variables |  |  |  |  |  |  |  |  |
| Extent of surgery | III | N/A | N/A | III | E-Tr | III | III | III |
| Use of radiation therapy | III | E-Tr | E-Tr | III | III | III | E-Tr | III |
| Use of chemotherapy | III | III | III | III | III | E-Tr | E-Tr | III |

Abbreviations: I-Model 1 (demographic and clinicopathologic variables), II-Model 2 (demographic, clinicopathologic, and hospital variables), III-Model 3 (demographic, clinicopathologic, hospital, and treatment variables); N/A-not applicable (i.e. all patients for breast cancer measures are female, all patients in MASTRT and LNoSurg are lymph node positive); E-CP-excluded because clinicopathologic variable is included in measure eligibility definition or would be unknown at the time established compliance status. E-Tr-excluded because treatment variable is a component of measure definition.
